# Unravelling the Biodiversity and Molecular Phylogeny of Needle Nematodes of the Genus *Longidorus* (Nematoda: Longidoridae) in Olive and a Description of Six New Species

**DOI:** 10.1371/journal.pone.0147689

**Published:** 2016-01-25

**Authors:** Antonio Archidona-Yuste, Juan A. Navas-Cortés, Carolina Cantalapiedra-Navarrete, Juan E. Palomares-Rius, Pablo Castillo

**Affiliations:** Instituto de Agricultura Sostenible (IAS), Consejo Superior de Investigaciones Científicas (CSIC), Avenida Menéndez Pidal s/n, 14004, Córdoba, Spain; Institute of Zoology, CHINA

## Abstract

The genus *Longidorus* includes a remarkable group of invertebrate animals of the phylum Nematoda comprising polyphagous root-ectoparasites of numerous plants including several agricultural crops and trees. Damage is caused by direct feeding on root cells as well as by transmitting nepoviruses that cause disease on those crops. Thus, correct identification of *Longidorus* species is essential to establish appropriate control measures. We provide the first detailed information on the diversity and distribution of *Longidorus* species infesting wild and cultivated olive soils in a wide-region in southern Spain that included 159 locations from which 449 sampling sites were analyzed. The present study doubles the known biodiversity of *Longidorus* species identified in olives by including six new species (***Longidorus indalus* sp. nov., *Longidorus macrodorus* sp. nov., *Longidorus onubensis* sp. nov., *Longidorus silvestris* sp. nov., *Longidorus vallensis* sp. nov., and *Longidorus wicuolea* sp. nov.**), two new records for wild and cultivate olives (*L*. *alvegus* and *L*. *vineacola*), and two additional new records for wild olive (*L*. *intermedius* and *L*. *lusitanicus*). We also found evidence of some geographic species associations to western (*viz*. *L*. *alvegus*, *L*. *intermedius*, *L*. *lusitanicus*, *L*. *onubensis* sp. nov., *L*. *vineacola*, *L*. *vinearum*, *L*. *wicuolea* sp. nov.) and eastern distributions (*viz*. *L*. *indalus* sp. nov.), while only *L*. *magnus* was detected in both areas. We developed a comparative study by considering morphological and morphometrical features together with molecular data from nuclear ribosomal RNA genes (D2–D3 expansion segments of 28S, ITS1, and partial 18S). Results of molecular and phylogenetic analyses confirmed the morphological hypotheses and allowed the delimitation and discrimination of six new species of the genus described herein and four known species. Phylogenetic analyses of *Longidorus* spp. based on three molecular markers resulted in a general consensus of these species groups, since lineages were maintained for the majority of species. This study represents the most complete phylogenetic analysis for *Longidorus* species to date.

## Introduction

The phylum Nematoda comprises the most species-rich metazoans on earth with a global distribution and estimated realistic number of species of *ca*. 10^5^ [1, 2, 3]. Soil nematode gross morphology tends to be highly conserved, making species identification a very difficult task [3, 4]. Accurate diagnostic studies of plant-parasitic nematode (PPN) species are important because of their implications in pest control and soil ecology [5]. With most nematode species likely remaining undescribed, efforts to catalogue and explain biodiversity need to be prioritised [6]. However species concept ranges among typological species (a community of specimens described by characteristic features of its type specimen), biological species (populations which successfully interbreed with each other), and phylogenetic species (phylogenetic lineages). All of these concepts have limitations, including the popular biological species concept which is restricted to sexual, outcrossing populations and excludes parthenogenetic organisms [7, 8]. Species delimitation in nematodes typically uses a phenotypic view of the animal, based in relatively few anatomical and morphological characters, such as lip region and female tail shape, pharyngeal glands, stylet shape and length, type of female reproductive system, etc. Additionally, many nematodes have complex life-cycles and it can be difficult to demonstrate the validity of a species by means of intercrossing of individuals and production of viable progeny. For these reasons the possibility of undescribed or misdescribed species is very high, as demonstrated by several authors [8, 9, 10, 11].

The family Longidoridae Thorne, 1935 [12] includes a wide and diverse group of migratory ectoparasitic nematode species, where the needle nematodes of the genus *Longidorus* Micoletzky, 1922 [13] is one of the most evolved group species of this family [14]. This genus includes a number of long to very long body (2–12 mm) specimens with long stylet (80–260 μm). They are polyphagous species of many plants including various agricultural crops, and cause damage by direct feeding on root cells as well as by transmitting nepoviruses (nepoviruses are spherical, single-stranded RNA of positive-sense) [15, 16, 17]. Some *Longidorus* spp. are cosmopolitan whilst others have a limited geographic distribution [14]. The genus *Longidorus* is a diverse group with about 160 nominal species [18, 19], but only 11 species (6.9%) (*L*. *apulus*, *L*. *arthensis*, *L*. *attenuatus*, *L*. *caespiticola*, *L*. *diadecturus*, *L*. *elongatus*, *L*. *fasciatus*, *L*. *leptocephalus*, *L*. *macrosoma*, *L*. *martini*, and *L*. *profundorum*) have been reported as virus vector, but transmitting seven out of the 38 known nepoviruses [15, 20]. Nepoviruses vectored by *Longidorus* species damage vegetable and fruit crops including: *Artichoke Italian latent virus*, *Cherry rosette disease virus*, *Tomato black ring virus*, *Raspberry ringspot virus*, *Arabis mosaic virus*, *Peach rosette mosaic virus*, and *Mulberry ringspot virus* [15, 20]. Therefore, correct identification of *Longidorus* species is essential to establish appropriate control measures. Species discrimination in *Longidorus* has classically been based mainly on morphology and morphometrics of diagnostic features. However morphologically based species characterization is complicated by a high degree of intraspecific variability within morphometrics, as well as slight interspecific differences that lead to substantial overlapping among *Longidorus* species and increase the risk of species miss-identification [10, 19]. As a result, taxonomic difficulties often arise from under- or over-estimation of intraspecific variability of certain morphological characters currently being used for species diagnosis.

Integrative taxonomy assembles and assimilates all available data and information to frame species limits (phenotypic, genotypic and phylogenetic) [7, 8]. Although this approach is more complex and has a higher cost than traditional taxonomy, its application reduce the degree of subjectivity that is common in traditional alpha taxonomic practices, as has been recently reported in studies showing the potential for these methods in the discovery of new and cryptic species in taxa poorly known or composed of morphologically conserved species [6, 8, 10, 19, 21, 22].

Recently, 68 *Longidorus* species (about 42% of total species) have been characterized molecularly, constituting a useful tool for molecular-based species identification. Molecular approaches using multiple regions of the ribosomal DNA (rDNA) genes sequences including (28S, 18S, and 5.8S genes and internal transcribed spacers (ITS1 and ITS2)), have been investigated to better understand the taxonomic relationships within the genus *Longidorus* [19, 21, 23, 24, 25, 26, 27, 28]. These molecular markers have been shown to be useful diagnostic tools in the characterization and phylogenetic relationships within Longidoridae, particularly in cases where morphological characters may lead to ambiguous interpretation, such as species in the *Xiphinema americanum* group [19, 21, 23, 24, 25, 26, 27, 28]. D2–D3 expansion segments of 28S rRNA and ITS1 rRNA have proven to be a powerful tool for providing accurate and molecular species identification in Longidoridae compared to partial 18S, since both molecular markers showed more species variability (nucleotides and indels) than partial 18S, which in some cases did not show enough resolution to distinguish species [19, 24, 25, 28, 29].

*Longidorus* species identification remains quite challenging when dealing with species that closely resemble one another and which co-occur in a region, as is often the case in the Iberian Peninsula. Furthermore, soil samples often contain mixed populations with more than one species in the same sample. In this study we focus mostly on the *Longidorus* species that occur throughout wild and cultivate olives at southern Spain. Morphological and morphometric evaluation as well as molecular sequencing of each *Longidorus* population were used simultaneously for species delineation and grouping specimens into species.

Olive, the emblematic tree of the Mediterranean Basin, is found in two forms, namely wild (*Olea europaea* subsp. *europaea* var. *sylvestris*) and cultivated (*Olea europaea* subsp. *europaea* var. *europaea*) [30]. Wild olives occur throughout many Mediterranean environments, characterized by semi-arid climatic conditions with different altitudes, plant communities and soils, including those with extreme dry conditions [30]. Cultivated olive is extensively grown in the Mediterranean Basin, as well as the subtropical regions of Australia, southern Africa, and North and South America [31]. Olive is the most cultivated non-tropical fruit trees and is among the most ancient crops in the Mediterranean Basin [31]. Approximately 10.5 million ha of cultivate olive are growing in the world, of which about 85% are in Mediterranean countries, including North Africa, and about 25% of them in Spain [32]. In Andalusia, southern Spain, cultivated olive trees cover more than 1.6 million ha accounting for 19% of the total surface area in an impressive monoculture [33, 34].

Both wild and cultivated olive trees serve as hosts to a large number of plant-parasitic nematodes, of which root-knot nematodes (*Meloidogyne* spp.), root-lesion nematodes (*Pratylenchus* spp.), spiral nematodes (*Helicotylenchus* spp.), and needle and dagger nematodes (*Longidorus* spp., *Xiphinema* spp.) are widely distributed and damage this crop [35, 36]. However, little information is available about needle nematodes associated with olive trees, except for the recent contribution of Palomares-Rius *et al*. [37] reporting *Longidorus magnus* Lamberti, Bleve-Zacheo and Arias 1982 [38] and *Longidorus* sp. According to Gutiérrez-Gutiérrez *et al*. [19] and other authors, 30 species of the genus *Longidorus* have been reported in Spain, mainly associated with fruit, forest, ornamental and vegetable plant species [19, 28, 39, 40].

With the aim of deciphering the biodiversity of *Longidorus* spp. infecting wild and cultivated olives in southern Spain, we sampled a total of 159 nine localities at the eight provinces of Andalusia where both olive types were present. In this survey we detected 40 populations of *Longidorus* species characterized by moderate to large body and stylet length, apparently morphologically related to other known *Longidorus* spp. This prompted us to carry out an integrative taxonomic study to assess the power of this approach for species identification within this complex genus.

The overall objective of this study was to test the congruence between morphological and molecular data within *Longidorus* species, and the specific objectives were: *i*) to identify and morphologically and morphometrically compare the 40 Spanish populations of *Longidorus* spp. detected in recent field samples from wild and cultivate olive-ecosystems; *ii*) to carry out a molecular characterisation of these *Longidorus* populations based on sequences of the D2–D3 expansion segments of the 28S nuclear ribosomal RNA gene, the ITS1 of rRNA, and partial 18S rRNA sequences; and *iii*) to study the phylogenetic relationships of *Longidorus* spp.

## Material and Methods

### Ethics Statement

No specific permits were required for the described fieldwork studies. Permission for sampling the olive orchards was granted by the landowner. The samples from wild olives were obtained in public areas, forests, and other natural areas studied and do not involve any species endangered or protected in Spain. The sites are not protected in any way.

### Soil collection and nematode extraction

**Fig 1:**
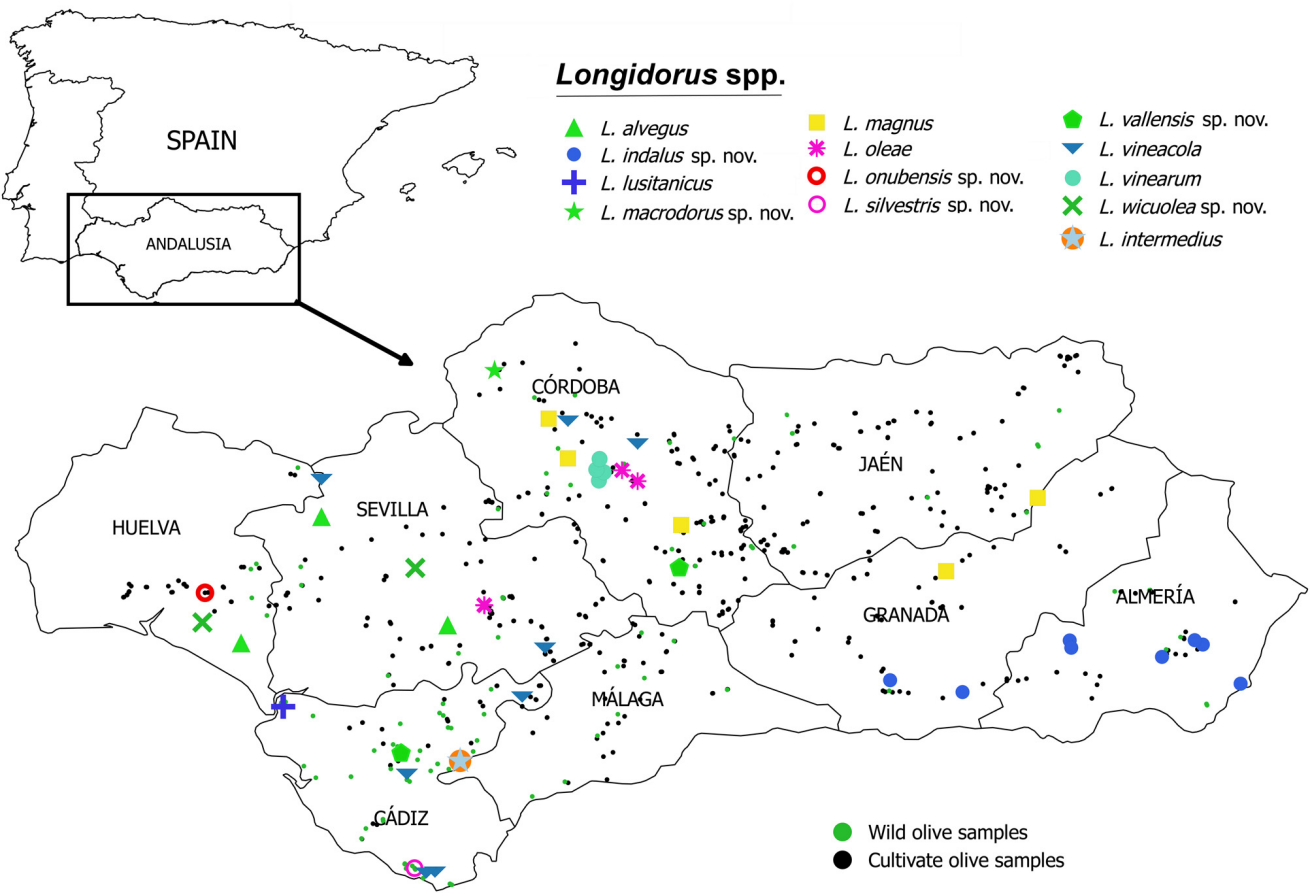
Geographic distribution of needle nematodes of the genus *Longidorus* in the present fieldworks on wild and cultivated olive in southern Spain. This map may be similar but not identical to other published maps of Andalusia and is therefore for illustrative purposes only on the sampling sites.

Nematodes were surveyed from 2012 to 2015 during the spring season in wild and cultivate olives groves in Andalusia, southern Spain (Table 1, Fig 1). Soil samples were collected for nematode analysis with a shovel from four to five trees in each sampling site. A total of 131 and 318 sampling sites from wild and cultivated olives, respectively, were arbitrarily chosen in the eight provinces of Andalusia where both olive types were present. The number of sampling sites was proportional to the area of wild and cultivated olive in each province (Table 1, Fig 1). Soil samples were collected from a 5- to 50-cm depth, in the close vicinity of active plant roots, discarding the upper 5-cm of topsoil to ensure that roots from weeds or other herbaceous plants were not included. All soil samples from each site were thoroughly mixed to obtain a single representative sample before nematode extraction.

**Table 1:** Taxa sampled for *Longidorus* species and sequences used in this study.

Species	Sampling site code	Administrative locality	Host-plant	D2–D3	ITS1	Partial 18S
**1. *Longidorus indalus* sp. nov.**	ST041	Las Tres Villas (Almería, Spain)	cultivated olive	KT308852	KT308878	KT308894
**2. *Longidorus indalus* sp. nov.**	AR046	Agua Amarga (Almería, Spain)	wild olive	KT308853	-	KT308895
**3. *Longidorus indalus* sp. nov.**	ST193	Lecrín (Granada, Spain)	cultivated olive	KT308854	KT308879	-
**4. *Longidorus indalus* sp. nov.**	ST042	Las Tres Villas (Almería, Spain)	cultivated olive	*	-	-
**5. *Longidorus indalus* sp. nov.**	AR044	Sorbas (Almería, Spain)	wild olive	*	-	-
**6. *Longidorus indalus* sp. nov.**	ST045	Sorbas (Almería, Spain)	cultivated olive	*	-	-
**7. *Longidorus indalus* sp. nov.**	JAO66	Lobras (Granada, Spain)	cultivated olive	*	-	-
**8. *Longidorus indalus* sp. nov.**	JAO73	Tabernas (Almería, Spain)	cultivated olive	*	-	-
**9. *Longidorus macrodorus* sp. nov.**	JAO06	La Grajuela (Córdoba, Spain)	cultivated olive	KT308855-KT308856	KT308880-KT308881	KT308896
**10. *Longidorus onubensis* sp. nov.**	ST005	Niebla (Huelva, Spain)	cultivated olive	KT308857-KT308858	KT308882-KT308883	KT308897
**11. *Longidorus silvestris* sp. nov.**	AR027	Tarifa (Cádiz, Spain)	wild olive	KT308859-KT308860	KT308884	KT308898
**12. *Longidorus vallensis* sp. nov.**	AR055	San José del Valle (Cádiz, Spain)	wild olive	KT308861	KT308885	KT308899
**13. *Longidorus vallensis* sp. nov.**	M0012	Cabra (Córdoba, Spain)	cultivated olive	KT308862	KT308886	-
**14. *Longidorus wicuolea* sp. nov.**	JAO95	Carmona (Sevilla, Spain)	cultivated olive	KT308863-KT308864	KT308887	KT308900
**15. *Longidorus wicuolea* sp. nov.**	AR101	Bonares (Huelva, Spain)	wild olive	KT308865-KT308866	KT308888-KT308889	-
**16. *L*. *alvegus* Roca *et al*., 1989**	JAO107	Utrera (Sevilla, Spain)	cultivated olive	KT308867		
**17. *L*. *alvegus* Roca *et al*., 1989**	AR110	Almadén de la Plata (Sevilla, Spain)	wild olive	*	-	-
**18. *L*. *alvegus* Roca *et al*., 1989**	AR099	El Rocío (Huelva, Spain)	wild olive	*	-	-
**19. *L*. *intermedius* Kozlowska & Seinhorst, 1979**	AR131	Jerez de la Frontera (Cádiz, Spain)	wild olive	KT308868	KT308890	-
**20. *L*. *lusitanicus* Macara, 1986**	J212B	Sanlúcar de Barrameda (Cádiz, Spain)	wild olive	KT308869	KT308891	KT308901
**21. *L*. *magnus* Lamberti *et al*., 1982**	ST146	Castril (Granada, Spain)	cultivated olive	KT308870	-	KT308902
**22. *L*. *magnus* Lamberti *et al*., 1982**	ST077	Espiel (Córdoba, Spain)	cultivated olive	*	-	-
**23. *L*. *magnus* Lamberti *et al*., 1982**	ST203	Morelábor (Granada, Spain)	cultivated olive	*	-	-
**24. *L*. *magnus* Lamberti *et al*., 1982**	JAO01	Villaviciosa (Córdoba, Spain)	cultivated olive	*	-	-
**25. *L*. *oleae* Gutiérrez-Gutiérrez et al., 2013**	AR112	Córdoba (Córdoba, Spain)	wild olive	KT308871	-	-
**26. *L*. *oleae* Gutiérrez-Gutiérrez et al., 2013**	AR113	Córdoba (Córdoba, Spain)	wild olive	*	-	-
**27. *L*. *oleae* Gutiérrez-Gutiérrez et al., 2013**	AR024	Marchena (Sevilla, Spain)	wild olive	*	-	-
**28. *L*. *oleae* Gutiérrez-Gutiérrez et al., 2013**	OL057	Marchena (Sevilla, Spain)	cultivated olive	*	-	-
**29. *L*.*vineacola* Sturhan & Weischer, 1954**	ST016	El Saucejo (Sevilla, Spain)	cultivated olive	KT308872	-	-
**30. *L*.*vineacola* Sturhan & Weischer, 1954**	AR031	Tarifa (Cádiz, Spain)	wild olive	KT308873	-	-
**31. *L*.*vineacola* Sturhan & Weischer, 1954**	AR006	Alcalá de los Gazules (Cádiz, Spain)	wild olive	*	-	-
**32. *L*.*vineacola* Sturhan & Weischer, 1954**	AR032	Tarifa (Cádiz, Spain)	wild olive	*	-	-
**33. *L*.*vineacola* Sturhan & Weischer, 1954**	ST117	Setenil de las Bodegas (Cádiz, Spain)	cultivated olive	*	-	-
**34. *L*.*vineacola* Sturhan & Weischer, 1954**	AR110	Almadén de la Plata (Huelva, Spain)	wild olive	*	-	-
**35. *L*.*vineacola* Sturhan & Weischer, 1954**	AR113	Córdoba (Córdoba, Spain)	wild olive	*	-	-
**36. *L*.*vineacola* Sturhan & Weischer, 1954**	JAO01	Villaviciosa (Córdoba, Spain)	cultivated olive	*	-	-
**37. *L*. *vinearum* Bravo & Roca, 1995**	AR059	Santa Mª de Trassierra (Córdoba, Spain)	wild olive	KT308874	KT308892	KT308903
**38. *L*. *vinearum* Bravo & Roca, 1995**	AR066	Santa Mª de Trassierra (Córdoba, Spain)	wild olive	KT308875	KT308893	-
**39. *L*. *vinearum* Bravo & Roca, 1995**	AR097	Santa Mª de Trassierra (Córdoba, Spain)	wild olive	KT308876	-	-
**40. *L*. *vinearum* Bravo & Roca, 1995**	AR111	Santa Mª de Trassierra (Córdoba, Spain)	wild olive	KT308877	-	-

(-) Not obtained or not performed.

(*) Sequenced population but not deposited in GenBank database, since was identical to other sequences of the same species.

Nematodes were extracted from a 500-cm^3^ sub-sample of soil using magnesium sulphate centrifugal-flotation and a modification of Cobb´s decanting and sieving methods [41, 42]. The soil was washed thoroughly with tap water through a 710-μm mesh sieve, and the filtered water was collected in a beaker and thoroughly mixed with 4% kaolin (v/v). This mixture was centrifuged at 1,100×g for 4 min, and the supernatants discarded. Pellets were resuspended in 250 ml MgSO4 (δ = 1.16) and the new suspensions were centrifuged at 1,100×g for 3 min. The supernatants were sieved through a 5 μm mesh, and nematodes collected on the sieve were washed with tap water [42]. The nematode sample was poured into a counting dish (8 cm L × 8 cm W × 1.5 cm H) and the nematodes were identified and counted under a Leica MZ12, stereomicroscope (Leica Microsystems, Wetzler, Germany). PPN from soil samples were identified to genus, and then we focussed on the species delineation of needle nematodes of the genus *Longidorus*. Later on, abundance and prevalence of each *Longidorus* species was estimated. Abundance was calculated as the mean number of *Longidorus* nematodes per 500 cm^3^ of soil for all samples. The prevalence was computed by dividing the number of samples in which the *Longidorus* species was detected by the total number of samples and expressed as a percentage.

### Morphological studies

*Longidorus* specimens for light microscopy were killed by gentle heat, fixed in a solution of 4% formaldehyde + 1% propionic acid and processed to pure glycerine using Seinhorst’s method [43]. Specimens were examined using a Zeiss III compound microscope with Nomarski differential interference contrast at up to 1,000x magnification. The morphometric study of each nematode population included classical diagnostic features in longidoridae (i.e. de Man body ratios, lip region and amphid shape, oral aperture-guiding ring, odontostyle and odontophore length) [44]. All measurements were expressed in micrometers (μm), unless otherwise indicated in text. For line drawing of the new species, light micrographs were imported to CorelDraw software version X6 (Corel Corporation, London, UK) and redrawn. All other abbreviations used are as defined in Jairajpuri & Ahmad [44]. In addition, a comparative morphological and morphometrical study of type specimens of some species were conducted with specimens kindly provided by Dr. A. Troccoli, from the nematode collection at the Istituto per la Protezione Sostenibile delle Piante (IPSP), Consiglio Nazionale delle Ricerche (CNR), Bari, Italy (*viz*. *Longidorus lusitanicus* Macara 1985 [45], *Longidorus vinearum* Bravo & Roca, 1995 [46]), and Dr. A. Navas from the Nematode Collection of the Spanish National Museum of Natural Sciences-CSIC, Madrid, Spain (*viz*. *Longidorus carpetanensis* Arias, Andrés & Navas, 1986 [47] and *Longidorus unedoi* Arias, Andrés & Navas, 1986 [47]).

### DNA extraction, PCR and sequencing

For molecular analyses, in order to avoid mistakes in the case of mixed populations in the same sample, two live nematodes from each sample were temporary mounted in a drop of 1M NaCl containing glass beads (to avoid nematode crushing/damaging specimens) to ensure specimens conformed in form to the unidentified populations of *Longidorus*. Morphometrics and photomicrographs recorded during this initial study were not used as part of the morphological study or analyses. Following morphological confirmation, the specimens were removed from the slides and DNA extracted. Nematode DNA was extracted from single individuals and PCR assays were conducted as described by Castillo *et al*. [48]. One nematode specimen of each sample was transferred to an Eppendorf tube containing 16 μl ddH_2_O, 2 μl 10x PCR buffer and 2 μl proteinase K (600 μg/ml) (Promega, Benelux, The Netherlands) and crushed during 2 min with a micro-homogeniser, Vibro Mixer (Zürich, Switzerland). The tubes were incubated at 65°C (1 h), then at 95°C (15 min), and finally at 80°C (15 min). One μl of extracted DNA was transferred to an Eppendorf tube containing: 2.5 μl 10X NH4 reaction buffer, 0.75 μl MgCl2 (50mM), 0.25 μl dNTPs mixture (10mM each), 0.75 μl of each primer (10mM), 0.2 μl BIOTAQ DNA Polymerase (BIOLINE, UK) and ddH_2_O to a final volume of 25 μl. The D2–D3 expansion segments of 28S rRNA was amplified using the D2A (5’-ACAAGTACCGTGAGGGAAAGTTG-3’) and D3B (5’-TCGGAAGGAACCAGCTACTA-3’) primers [49]. The ITS1 region was amplified using forward primer 18S (5´TTGATTACGTCCCTGCCCTTT-3´) [50] and reverse primer rDNA1 (5´-ACGAGCCGAGTGATCCACCG-3´) [51]. Finally, the portion of the 18S-rRNA was amplified using primers 988F (5´-CTCAAAGATTAAGCCATGC-3´), 1912R (5´TTTACGGTCAGAACTAGGG-3´), 1813F (5´- CTGCGTGAGAGGTGAAAT-3´) and 2646R (5´-GCTACCTTGTTACGACTTTT-3´) [52].

PCR cycle conditions were: one cycle of 94°C for 2 min, followed by 35 cycles of 94°C for 30 s, annealing temperature of 55°C for 45 s, 72°C for 3 min, and finally one cycle of 72°C for 10 min. PCR products were purified after amplification using ExoSAP-IT (Affmetrix, USB products), quantified using a Nanodrop spectrophotometer (Nanodrop Technologies, Wilmington, DE, USA) and used for direct sequencing in both directions using the primers referred to above. The resulting products were purified and run on a DNA multicapillary sequencer (Model 3130XL genetic analyser; Applied Biosystems, Foster City, CA, USA), using the BigDye Terminator Sequencing Kit v.3.1 (Applied Biosystems, Foster City, CA, USA), at the Stab Vida sequencing facilities (Caparica, Portugal). The newly obtained sequences were submitted to the GenBank database under accession numbers indicated on the phylogenetic trees and in Table 1.

### Phylogenetic analysis

D2–D3 expansion segments of 28S rRNA, ITS1, and partial 18S rRNA sequences of different *Longidorus* spp. from GenBank were used for phylogenetic reconstruction. Outgroup taxa for each dataset were chosen according to previous published data [19, 25, 26, 52, 53]. The newly obtained and published sequences for each gene were aligned using MAFFT ver. 7 [54], strategy FFT-NS-1 with default parameters. Sequence alignments were manually edited using BioEdit [55]. Percentage similarity between sequences was calculated using the sequence identity matrix using BioEdit. For that, the score for each pair of sequences was compared directly and all gap or place-holding characters were treated as a gap [55]. When positions of both sequences have a gap they do not contribute [55]. Phylogenetic analyses of the sequence data sets were performed based on Bayesian inference (BI) using MRBAYES 3.1.2 [56]. The best fitted model of DNA evolution was obtained using JMODELTEST v. 2.1.7 [57] with the Akaike Information Criterion (AIC). The Akaike-supported model, the base frequency, the proportion of invariable sites, and the gamma distribution shape parameters and substitution rates in the AIC were then used in phylogenetic analyses. BI analyses were performed under SYM+I+G (namely, symmetrical of invariable sites and a gamma-shaped distribution) model for D2–D3 expansion segments of 28S rRNA, TVM+I+G and TIM3+I+G (namely, transversional and a transitional of invariable sites and a gamma-shaped distribution) models for the two ITS1 region datasets, TVMef+I+G (namely, equal-frequency transversional of invariable sites and gamma-shaped distribution) model for the partial 18 S rDNA. These BI analyses were run separately per dataset using four chains for 2 × 10^6^, 1 and 1 × 10^6^, and 3 × 10^6^ generations, respectively. The Markov chains were sampled at intervals of 100 generations. Two runs were performed for each analysis. After discarding burn-in samples and evaluating convergence, the remaining samples were retained for further analyses. The topologies were used to generate a 50% majority rule consensus tree. Posterior probabilities (PP) are given on appropriate clades. Trees were visualised using TreeView [58].

### Nomenclatural Acts

The electronic edition of this article conforms to the requirements of the amended International Code of Zoological Nomenclature (ICZN), and hence the new names contained herein are available under that Code from the electronic edition. This published work and the nomenclatural acts it contains have been registered in ZooBank, the online registration system for the ICZN. The ZooBank Life Science Identifiers (LSIDs) can be resolved and the associated information viewed through any standard web browser by appending the LSID to the prefix "http://zoobank.org/". The LSID for this publication is: urn: lsid:zoobank.org:pub: C8230A9D‐FD45‐4AA4‐9ABF‐8445E8001CCC. The electronic edition of this work was published in a journal with an ISSN, and has been archived and is available from the following digital repositories: PubMed Central, LOCKSS.

## Results

### Taxon Sampling, abundance and prevalence of *Longidorus* species

All positive *Longidorus* spp.-sampling sites for this study, including specimens used in morphological and/or genetic analyses, are shown in Table 1 and Fig 1. Ten *Longidorus* species were associated with wild olive (*viz*. *Longidorus alvegus* Roca, Pereira and Lamberti 1989 [59], *Longidorus indalus* sp. nov., *Longidorus intermedius* Kozlowska & Seinhorst, 1979 [60], *L*. *lusitanicus*, *Longidorus oleae* Gutiérrez-Gutiérrez, Cantalapiedra-Navarrete, Montes-Borrego, Palomares-Rius & Castillo, 2013 [19], *Longidorus silvestris* sp. nov., *Longidorus vallensis* sp. nov., *Longidorus vineacola* Sturhan & Weischer, 1964 [61], *L*. *vinearum*, and *Longidorus wicuolea* sp. nov.), whereas nine *Longidorus* species (*viz*. *L*. *alvegus*, *Longidorus indalus* sp. nov., *Longidorus macrodorus* sp. nov., *L*. *magnus*, *L*. *oleae*, *Longidorus onubensis* sp. nov., *Longidorus vallensis* sp. nov., *L*. *vineacola*, and *Longidorus wicuolea* sp. nov.) were associated with cultivated olive in Andalusia (Table 1; Fig 1). Except for *L*. *alvegus*, *L*. *indalus* sp. nov. and *L*. *vineacola*, that occurred in both olive types, all the remaining identified species where present only in either wild or cultivated olives.

*Longidorus* spp. were present in low to moderate densities (from 1 to 33 nematodes per 500 cm^3^ of soil), and were moderately distributed in both wild and cultivated olives (Table 2). The overall prevalence of *Longidorus* spp. in wild olives was 16.03% (21 out of 131 samples) whereas in cultivated olives was 5.97% (19 out of 318 samples) (Tables 1 and 2). Although wild and cultivated olives were present in all of the eight provinces of Andalusia, the genus *Longidorus* was not detected in Jaén and Málaga provinces, and in Granada only in cultivated olives (4 samples out of 39) (Table 2, Fig 1). The three most prevalent *Longidorus* species, *L*. *indalus* sp. nov., *L*. *oleae*, and *L*. *vineacola*, were detected in both wild and cultivated olives, as well as *L*. *alvegus*, *L*. *vallensis* sp. nov. and *L*. *wicuolea* sp. nov. but with lower prevalence (Tables 1 and 2). *Longidorus vineacola* was rather moderately distributed among the studied zones having the highest overall prevalence in both wild and cultivated olives (Tables 1 and 2). However, some other *Longidorus* species showed a lower prevalence and were only detected either in wild (*L*. *lusitanicus*, *L*. *silvestris* sp. nov. and *L*. *vinearum*) or in cultivated olive (*L*. *macrodorus* sp. nov., *L*. *magnus*, *L*. *onubensis* sp. nov.) (Tables 1 and 2).

**Table 2:** Soil nematode population density (number of specimens) and prevalence (%) of Longidorus spp. in wild and cultivated olives in provinces of Andalusia, southern Spain.^a^

	Almería province		Cádiz province		Córdoba province		Granada province		Huelva province		Sevilla province	
*Longidorus* species	Wild	cultivated	Wild	cultivated	Wild	cultivated	Wild	cultivated	Wild	cultivated	Wild	cultivated
**number of samples**	10	23	56	19	29	72	1	39	8	20	9	60
***L*. *alvegus* Roca *et al*., 1989**	-	-	-	-	-	-	-	-	3 (12.5)	-	1 (11.1)	12 (1.7)
***L*. *indalus* sp. nov.**	3 (20.0)	18 (21.7)	-	-	-	-	-	1 (5.1)	-	-	-	-
***L*. *intermedius* Kozlowska & Seinhorst, 1979**	-	-	33 (1.8)	-	-	-	-	-	-	-	-	-
***L*. *lusitanicus* Macara, 1986**	-	-	8 (1.8)	-	-	-	-	-	-	-	-	-
***L*. *macrodorus* sp. nov.**	-	-	-	-	-	1 (1.4)	-	-	-	-	-	-
***L*. *magnus* Lamberti *et al*., 1982**	-	-	-	-	-	3 (2.8)	-	3 (5.1)	-	-	-	-
***L*. *oleae* Gutiérrez-Gutiérrez et al., 2013**	-	-	-	-	2 (6.8)	-	-	-	-	-	2 (11.1)	2 (1.7)
***L*. *onubensis* sp. nov.**	-	-	-	-	-	-	-	-		3 (5.0)	-	-
***L*. *silvestris* sp. nov.**	-	-	11 (1.8)	-	-	-	-	-	-	-	-	-
***L*. *vallensis* sp. nov.**	-	-	3 (1.8)	-	-	3 (1.4)	-	-	-	-	-	-
***L*.*vineacola* Sturhan & Weischer, 1954**	-	-	4 (5.5)	1 (5.3)	1 (3.4)	1 (1.4)	-	-	1 (12.5)	-	-	4 (1.7)
***L*. *vinearum* Bravo & Roca, 1995**	-	-	-	-	14 (13.8)	-	-	-	-	-	-	-
***L*. *wicuolea* sp. nov.**	-	-	-	-	-	-	-	-	2 (12.5)	-	-	5 (1.7)

^a^ Population density was calculated as the mean of *Longidorus* nematodes per 500 cm^3^ of soil. The prevalence was computed by dividing the numbers of samples in which the *Longidorus* species was observed by the total number of samples and expressed as a percentage. Since no *Longidorus* spp. were detected in wild and cultivated olives in Jaén (9 wild olive and 58 cultivated olive samples) and Málaga (9 wild olive and 27 cultivated olive samples) provinces, data were not indicated in this table.

(-) not found

### Taxonomic treatment

Nematoda Linnaeus, 1758 [62]

Dorylaimida Pearse, 1942 [63]

Longidoridae Thorne, 1935 [12]

Longidorinae Thorne, 1935 [12]

*Longidorus* Micoletzky 1922 [13]

**Fig 2:**
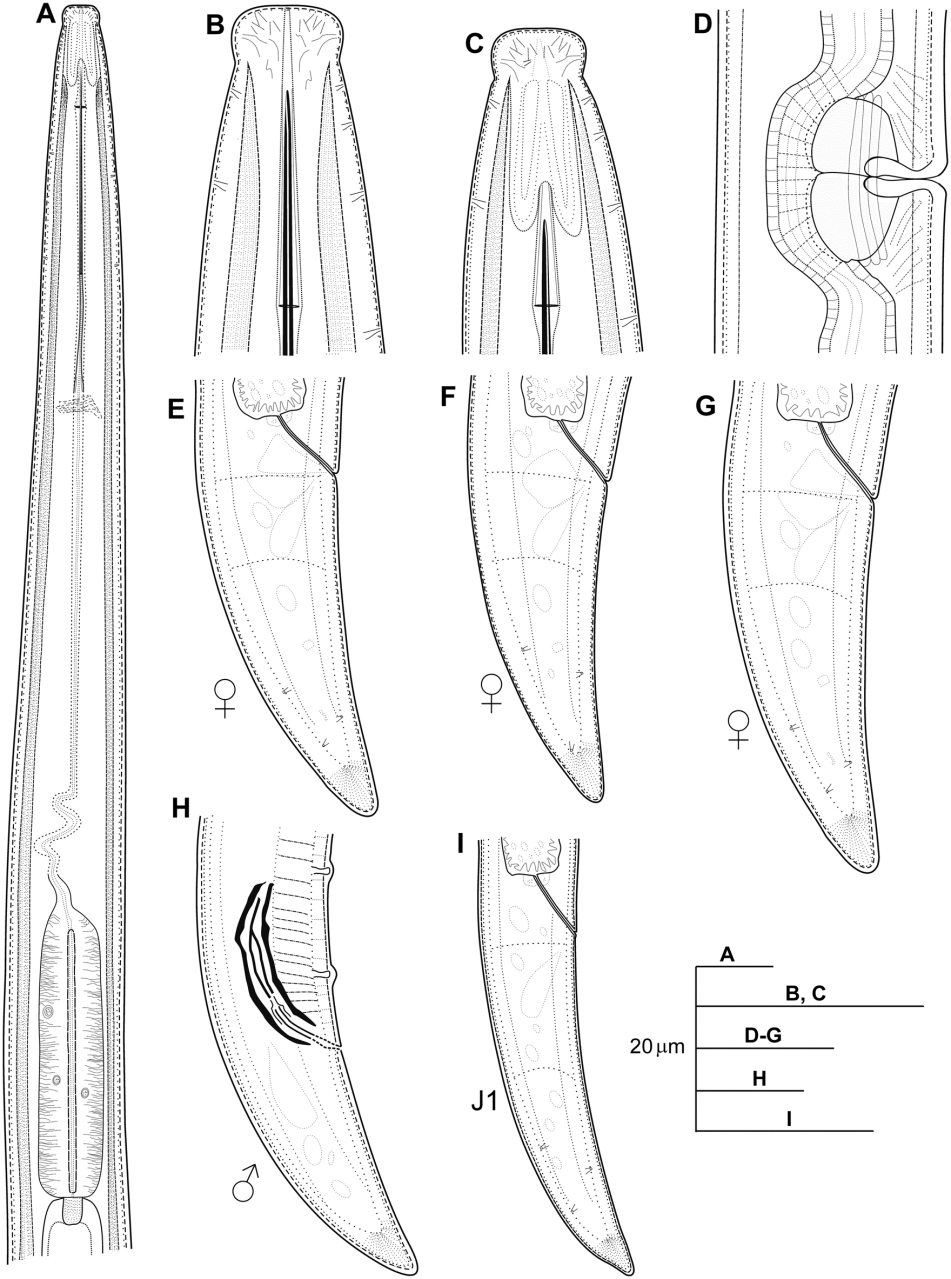
Line drawings of *Longidorus indalus* sp. nov. A) Pharyngeal region. B, C) Details of lip region. D) Vulval region. E-G) Female tails. H) Male tail. I) First-stage juvenile tail (J1).

**Fig 3:**
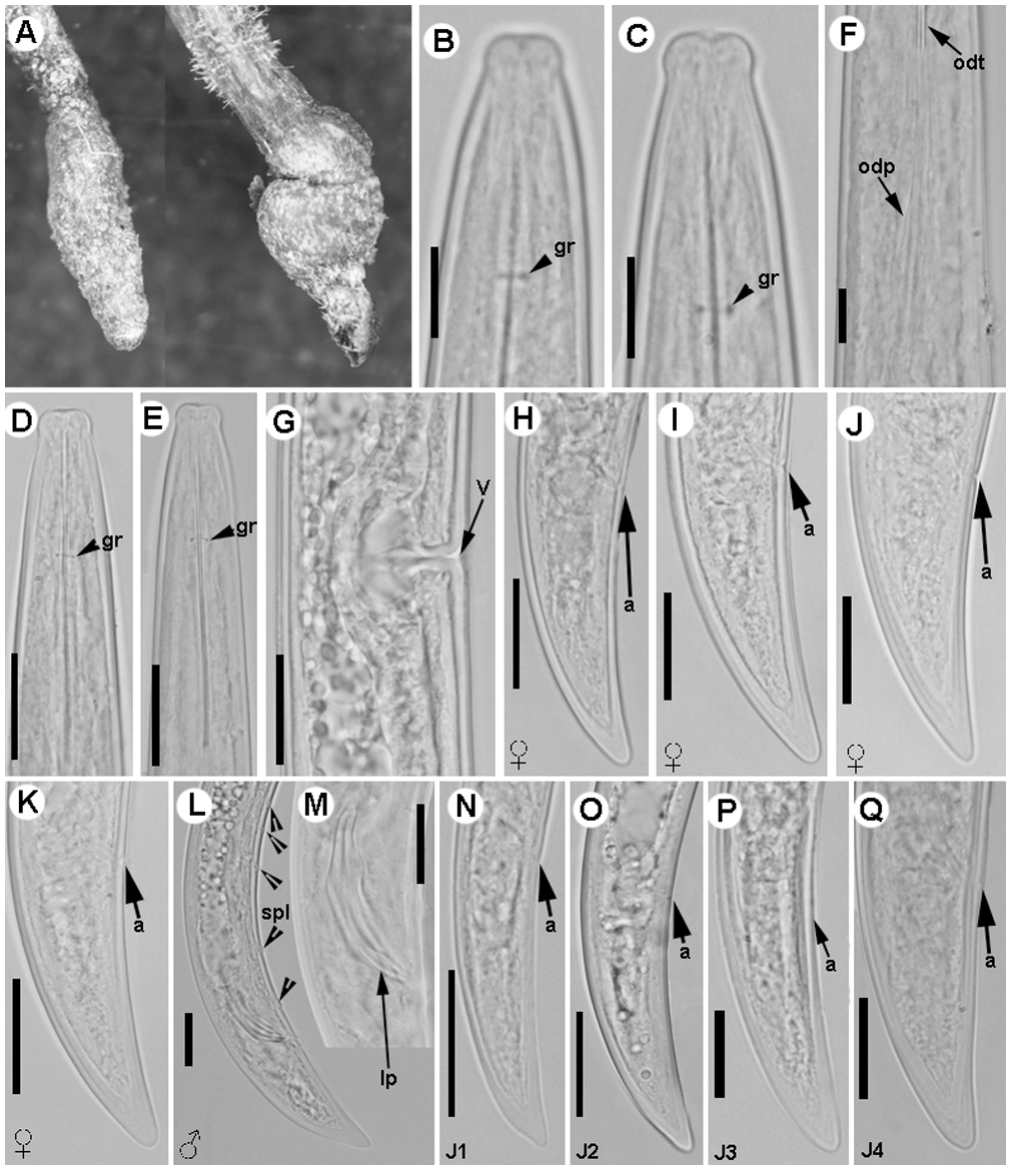
Light micrographs of *Longidorus indalus* sp. nov., female paratypes, male and juvenile stages. A) Olive apical galled roots infected by the nematode. B–E) Female anterior regions. F) Detail of odontostyle and odontophore. G) Vulval region. H-K) Female tails. L, M) Male tail with detail of spicules. N-Q) First-, second-, third-, and fourth-stage juvenile (J1–J4) tails, respectively. Abbreviations: a = anus; gr = guiding-ring; odt = odontostyle; odp = odontophore; lp = lateral accessory piece; spl = ventromedian supplements; v = vulva. Scale bars B, C, F = 10 μm; D, E, G-Q = 20 μm.

**Fig 4:**
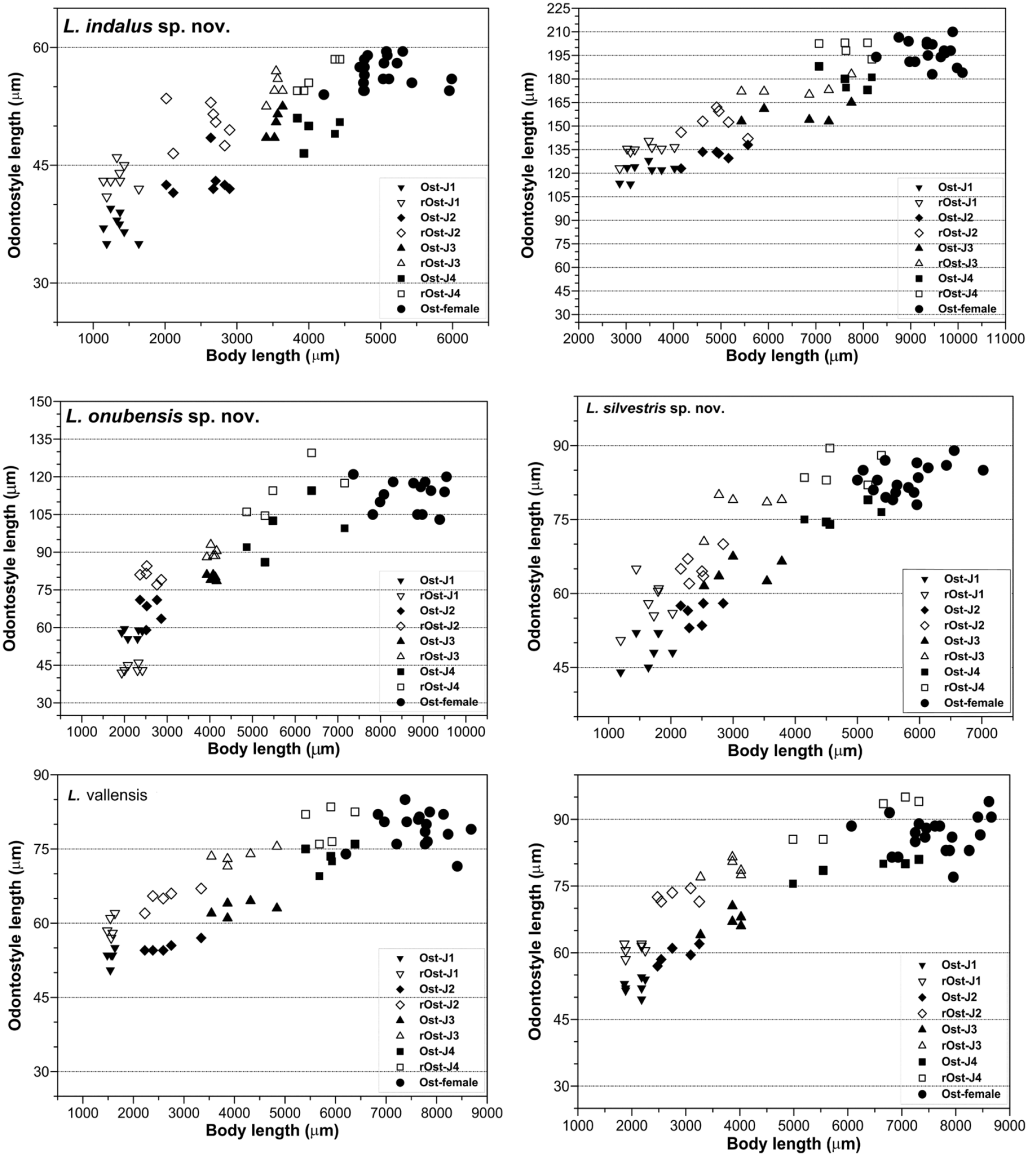
Relationship between body length and functional and replacement odontostyle (Ost and rOst, respectively) length in all developmental stages from first-stage juveniles (J1) to mature females of. A) *Longidorus indalus* sp. nov. B) *Longidorus macrodorus* sp. nov. C) *Longidorus onubensis* sp. nov. D) *Longidorus silvestris* sp. nov. E) *Longidorus vallensis* sp. nov. F) *Longidorus wicuolea* sp. nov.

***Longidorus indalus* Archidona-Yuste, Navas-Cortés, Cantalapiedra-Navarrete, Palomares-Rius & Castillo, sp. nov.** urn:lsid:zoobank.org:act:CE07DF59-E705-43D1-9CFF-1A8D793FA58D Figs 2–4.

#### Holotype

Adult female, collected from the rhizosphere of cultivated olive (*Olea europaea* subsp. *europaea* L.) (37°08'47.5"N, 002°43'31.7"W), at Las Tres Villas, Almería province, Spain; collected by G. Leon Ropero, April 11, 2013; mounted in pure glycerine and deposited in the nematode collection at Institute for Sustainable Agriculture (IAS) of Spanish National Research Council (CSIC), Córdoba, Spain (collection number ST41-21).

#### Paratypes

Female, male and juvenile paratypes extracted from soil samples collected from the same locality as the holotype; mounted in pure glycerine and deposited in the following nematode collections: Institute for Sustainable Agriculture (IAS) of Spanish National Research Council (CSIC), Córdoba, Spain (collection numbers ST41-01-ST41-17); two females at Istituto per la Protezione Sostenibile delle Piante (IPSP), Consiglio Nazionale delle Ricerche (CNR), Bari, Italy (ST41-20); two females at Royal Belgian Institute of Natural Sciences, Brussels, Belgium (RIT837); and four females at USDA Nematode Collection, Beltsville, MD, USA (T-6629p); collected by G. Leon Ropero, April 11, 2013.

#### Diagnosis

*Longidorus indalus* sp. nov. is characterized by a moderate long body (4.1–6.0 mm), assuming an open C-shaped when heat relaxed; lip region expanded distinctly set off from body contour, 8.5–10.0 μm wide and 3.0–4.5 μm high; guiding-ring located 19.0–27.5 μm from anterior end; relatively short odontostyle (53.5–60.5 μm); amphidial fovea pocket-shaped, slightly asymmetrically bilobed; vulva almost equatorial; female tail long, conoid, and bearing three pairs of caudal pores; c’ ratio (1.8–2.9); males extremely rare, only one male was found, with very short spicules (34.5 μm) and 5 ventromedian supplements; and specific D2–D3, ITS1 rRNA and partial 18S rRNA sequences (GenBank accession numbers KT308852-KT308854, KT308878-KT308879, and KT308894-KT308895, respectively). According to the polytomous key Chen *et al*. [64] and the supplement by Loof and Chen [65], the new species has the following code (codes in parentheses are exceptions): A1-B1-C2-D4-E2-F23-G3-H56-I12.

#### Etymology

The species name is derived from the name ‘*indalo*’ a prehistoric symbol found in a cave of Almería, the province of the locality where the type specimens were collected.

#### Description of taxa

**Female**. Body somewhat helicoid to arcuate, cylindrical, relatively long and thin, slightly tapering towards at both ends. When heat relaxed, body ventrally curved in open C-shaped. Cuticle thin appearing smooth under low magnifications, 1.9 ± 0.4 (1.5–2.5) μm thick at mid body, but slightly thicker (3.1 ± 0.8 (2.0–4.5) μm) and marked by very fine superficial transverse striate mainly in tail region, as shown by higher magnifications. Lip region expanded distinctly set off from body contour, anteriorly flattened, 9.2 ± 0.5 (8.5–10.0) μm wide and 3.9 ± 0.4 (3.0–4.5) μm high. Amphidial fovea pocket-shaped, slightly asymmetrically bilobed with lobes occupying about 1/3 part of distance between oral aperture and guiding-ring. Stylet guiding-ring single, located 2.8 ± 0.2 (2.5–3.2) times lip region diam. from anterior end. Odontostyle typical of genus, 1.5 ± 0.2 (1.1–1.9) times as long as odontophore, straight or slightly arcuate; odontophore weakly developed, with rather weak basal swellings. Nerve ring surrounding odontophore base at 94.3 ± 4.9 (85.5–107.0) μm from anterior end. Anterior slender part of pharynx usually coiled in its posterior region. Basal bulb short and cylindrical, 92.3 ± 9.6 (72.0–103.5) μm long and 15.6 ± 1.8 (12.5–19.5) μm in diam. Glandularium 83.3 ± 8.7 (63.5–96.0) μm long. Dorsal pharyngeal gland nucleus (DN) and ventrosublateral nuclei (SVN) located at 33.5 ± 4.0 (27.3–39.5)% and 57.0 ± 4.4 (48.9–63.7)% of distance from anterior end of pharyngeal bulb, respectively. Nucleolus of DN larger than nucleoli of two SVN (4.0–4.5 vs 3.0–3.5 μm). Cardia conoid-rounded, 8.2 ± 0.2 (5.5–10.5) μm long. Lateral chord ca 9.6 μm wide at mid-body or ca 28% of corresponding body diam. Reproductive system with both genital branches equally developed, each branch 314–800 μm long, with reflexed ovaries very variable in length (85.5–161 μm long). Vulva in form of a transverse slit, located slightly anterior of the middle of the body, vagina perpendicular to body axis, 13.7 ± 3.2 (8.5–16.5) μm long or 24–47% of corresponding body width, surrounded by well-developed muscles. Genital branches equally developed, 9.7 ± 2.4 (6.8–13.9), 9.9 ± 2.2 (6.7–13.9)% of body length, respectively. Uteri highly variable in length (250–594 μm long), without sperm cells in all female specimens examined; sphincter well-developed, between uterus and oviduct. Eggs mature observed in some gravid female specimens along uterus from one gonoduct, 228.3 ± 8.0 (220.0–236.0) μm long and 32.2 ± 2.0 (30.0–34.0) μm wide. Anterior and posterior oviduct of similar size. Prerectum very variable in length, 673.1 ± 120.7 (489.0–861.0) μm long, and rectum 17.9 ± 3.2 (8.5–16.5) μm long ending in anus as a small rounded slit. Tail long, bluntly conoid, with rounded terminus, bearing three pairs of caudal pores.

#### Male

Extremely rare, only one male specimen was found but not in type locality. Morphologically similar to female except for genital system, but with posterior region slightly curved ventrally. Male genital tract diorchic with testes opposed, containing multiple rows of different stages of spermatogonia. Tail conoid, dorsally conoid and ventrally concave with rounded terminus and thickened outer cuticular layer. Spicules very short, moderately developed and slightly curved ventrally; lateral guiding pieces more or less straight or with curved proximal end. Low number of supplements, one pair of adanal and 4 mid-ventral supplements.

#### Juveniles

Morphologically similar to adults, but smaller. All four juvenile stages were found, being distinguishable by relative lengths of body and functional and replacement odontostyle (Table 3, Figs 3 and 4; [66, 67]). J1s were characterised by a bluntly conoid tail with a c’ ratio ≥ 3.2, well curved dorsally with a dorsal depression at hyaline region level (Fig 3) odontostyle length *ca* 37 μm, and shorter distance from anterior end to stylet guiding-ring than that in adult stages. However, morphology in second-, third- and fourth-stages (except for undeveloped genital structures) similar to that of female, including conoid tail shape, becoming progressively shorter and stouter in each moult and shorter distance from anterior end to guiding-ring in each moult.

**Table 3:** Morphometrics of females, males and juvenile stages of *Longidorus indalus* sp. nov. from the rhizosphere of cultivated and wild olives at several localities (Almería province) southern Spain^a^.

Host/locality, sample code	cultivated olive, Las Tres Villas (Almería province), ST041						Wild olive Agua Amarga (Almería province), AR46
Characters/ratios^b^	Holotype	Paratype Females	J1	J2	J3	J4	Females
**n**		21	9	7	5	6	6
**L (mm)**	5.3	5.0 ± 0.42 (4.2–6.0)	1.34 ± 0.16 (1.14–1.64)	2.56 ± 0.35 (2.16–2.84)	3.54 ± 0.08 (3.41–3.64)	4.10 ± 0.24 (3.84–4.43)	4.6 ± 0.36 (4.1–5.1)
**a**	134.1	143.5 ± 14.4 (115.0–178.2)	78.2 ± 6.2 (71.5–89.5)	100.0 ± 14.2 (85.8–128.6)	124.4 ± 10.5 (115.4–140.9)	124.7 ± 9.1 (118.3–142.7)	129.7 ± 10.6 (116.9–145.6)
**b**	14.7	15.3 ± 2.5 (10.2–19.1)	7.7 ± 2.0 (5.1–11.3)	10.0 ± 0.9 (9.0–11.7)	12.0 ± 2.9 (9.7–16.9)	14.9 ± 2.5 (10.8–17.1)	18.7 ± 3.4 (15.6–24.2)
**c**	95.4	98.2 ± 10.4 (81.0–122.8)	33.2 ± 2.2 (30.9–37.6)	50.3 ± 6.6 (40.3–58.0)	66.2 ± 4.5 (62.2–73.6)	77.8 ± 7.2 (71.2–91.5)	103.8 ± 10.1 (89.1–113.7)
**c´**	2.5	2.3 ± 0.2 (1.9–2.9)	3.4 ± 0.2 (3.2–3.8)	3.0 ± 0.3 (2.5–3.4)	2.8 ± 0.2 (2.6–3.1)	2.4 ± 0.2 (2.0–2.6)	1.9 ± 0.1 (1.8–2.1)
**V**	48.5	47.6 ± 1.2 (45.5–50.0)	-	-	-	-	49.8 ± 1.8 (47.0–52.0)
**Odontostyle**	56.5	56.8 ± 1.8 (54.0–59.5)	37.2 ± 1.7 (35.0–39.5)	43.1 ± 2.4 (41.5–48.5)	50.3 ± 1.8 (48.5–52.5)	49.9 ± 2.0 (46.5–52.5)	55.2 ± 1.4 (53.5–57.5)
**Odontophore**	35.0	37.5 ± 4.7 (30.0–51.0)	22.5 ± 1.6 (20.0–24.0)	26.9 ± 3.8 (21.5–30.5)	34.9 ± 2.0 (32.5–36.5)	30.2 ± 7.0 (25.0–42.5)	41.3 ± 3.7 (37.0–46.0)
**Replacement odontostyle**	-	-	43.4 ± 1.6 (41.0–46.0)	50.3 ± 2.6 (46.5–53.5)	54.9 ± 1.7 (52.5–57.0)	55.9 ± 2.1 (54.0–58.5)	-
**Lip region diam.**	8.5	9.2 ± 0.5 (8.5–10.0)	6.6 ± 0.4 (6.0–7.5)	7.6 ± 0.3 (7.0–8.0)	8.2 ± 0.8 (7.5–9.5)	8.6 ± 0.4 (8.0–9.0)	8.9 ± 0.4 (8.5–9.5)
**Oral aperture-guiding ring**	26.5	25.7 ± 1.1 (23.5–27.5)	16.6 ± 1.0 (15.5–18.0)	20.2 ± 1.3 (18.0–21.5)	22.4 ± 1.1 (21.5–24.0)	22.8 ± 0.9 (22.0–24.0)	22.9 ± 2.0 (19.0–24.0)
**Tail length**	55.5	51.7 ± 4.9 (45.5–59.5)	40.3 ± 2.6 (37.0–43.5)	50.7 ± 2.1 (47.5–54.0)	53.6 ± 3.7 (48.5–58.5)	53.8 ± 1.9 (51.0–56.5)	44.6 ± 3.7 (41.0–50.5)
**J**	11.5	10.0 ± 1.3 (7.5–12.0)	5.3 ± 0.7 (4.5–6.0)	6.2 ± 0.3 (6.0–6.5)	7.4 ± 1.2 (6.5–9.0)	7.8 ± 1.3 (6.5–9.0)	9.0 ± 0.8 (8.0–10.0)

^a^ Measurements are in μm (except for L) and in the form: mean ± standard deviation (range).

^b^ Abbreviations as defined in Jairajpuri & Ahmad [44]. a, body length/maximum body width; b, body length/pharyngeal length; c, body length/tail length; c', tail length/body width at anus; V (distance from anterior end to vulva/body length) x 100; T (distance from cloacal aperture to anterior end of testis/body length) x 100; J (hyaline tail region length).

#### Measurements, morphology and distribution

Morphometric variability is described in Tables 3 and 4 and morphological traits in Figs 2, 3 and 4. In addition to the type locality, *Longidorus indalus* sp. nov. was extracted from five cultivated olive samples causing enlarged swellings of root tips (Fig 3), and two wild olive samples of several localities distributed in Almería and Granada province, being one of the two species (together with *L*. *magnus*) located on Eastern Andalusia (Table 1, Fig 1).

**Table 4:** Morphometrics of females, males and juvenile stages of *Longidorus indalus* sp. nov. from the rhizosphere of cultivated and wild olives at several localities (Almería and Granada provinces) southern Spain^a^.

Host-plant	Cultivated olive					Wild olive
Locality, sample code	Tabernas (Almería province), JAO73		Las Tres Villas (Almería province), ST042	Lecrín (Granada province), ST193	Sorbas (Almería province), ST045	Sorbas (Almería province), AR044
Characters/ratios^b^	Females	Males	Females	Female	Females	Female
**n**	3	1	3	1	3	1
**L (mm)**	4.7 ± 0.27 (4.5–5.0)	4.3	5.2 ± 0.42 (4.8–5.7)	4.3	5.0 ± 0.23 (4.8–5.3)	5.7
**a**	137.6 ± 17.0 (119.9–153.8)	155.4	136.2 ± 19.1 (119.2–156.9)	152.3	140.5 ± 14.5 (126.2–155.2)	128.2
**b**	15.7 ± 2.1 (13.6–17.7)	20.0	14.5 ± 0.4 (14.2–14.9)	18.6	14.9 ± 1.2 (14.1–16.3)	13.8
**c**	98.8 ± 7.3 (93.3–107.1)	97.1	104.9 ± 0.4 (98.6–112.6)	92.4	98.0 ± 1.6 (97.0–99.9)	109.7
**c´**	2.1 ± 0.3 (1.8–2.4)	1.9	2.1 ± 0.1 (2.0–2.3)	2.4	2.4 ± 0.2 (2.2–2.6)	2.0
**V or T**	48.3 ± 2.1 (46.0–50.0)	27.3	47.5 ± 0.5 (47.0–48.0)	48.0	46.5 ± 1.5 (45.0–48.0)	50.5
**Odontostyle**	58.8 ± 2.5 (56.0–60.5)	57.5	57.8 ± 1.1 (57.0–58.5)	60.0	55.8 ± 1.3 (54.5–57.0)	59.5
**Odontophore**	35.3 ± 4.0 (31.0–39.0)	39.0	37.0 ± 1.4 (36.0–38.0)	40.0	36.8 ± 2.9 (33.5–39.0)	41.5
**Lip region diam.**	8.8 ± 0.6 (8.5–9.5)	8.5	9.0 ± 0.7 (8.5–9.5)	9.5	8.8 ± 0.6 (8.5–9.5)	8.5
**Oral aperture-guiding ring**	24.8 ± 0.8 (24.0–25.5)	25.5	25.0 ± 0.0 (25.0–25.0)	24.0	25.0 ± 1.3 (23.5–26.0)	23.5
**Tail length**	47.8 ± 0.8 (47.0–48.5)	44.0	49.5 ± 4.8 (45.0–54.5)	47.0	51.3 ± 3.1 (48.0–54.0)	52.0
**Spicules**	-	34.5	-	-	-	-
**Lateral accessory piece**	-	13.5	-	-	-	-
**Supplements**	-	5	-	-	-	-
**J**	9.5 ± 0.5 (9.0–10.0)	9.5	10.3 ± 0.8 (9.5–11.0)	9.5	9.2 ± 1.2 (8.5–10.5)	10.0

^a^ Measurements are in μm (except for L) and in the form: mean ± standard deviation (range).

^b^ Abbreviations as defined in Jairajpuri & Ahmad [44]. a, body length/maximum body width; b, body length/pharyngeal length; c, body length/tail length; c', tail length/body width at anus; V (distance from anterior end to vulva/body length) x 100; T (distance from cloacal aperture to anterior end of testis/body length) x 100; J (hyaline tail region length).

#### Relationships

According to the polytomous key by Chen *et al*. [64] and the supplement by Loof and Chen [65], and on the basis of sorting on matrix codes A (odontostyle length), B (lip region width), C (distance of guiding-ring from anterior body end), D (lip region shape), and E (shape of amphidial pouch), *L*. *indalus* sp. nov. is closely related to *L*. *carpetanensis* and *L*. *unedoi* from which it can be differentiated by a combination of these characters discussed below, but particularly in female and male tail shape (bluntly conoid *vs* conical, dorsally convex) (Fig 3, S1 Fig). *Longidorus indalus* sp. nov. differs from *L*. *carpetanensis* by a longer body length (4.1–5.7 *vs* 3.5–4.4 mm), higher a ratio (115.0–178.2 *vs* 96.0–118.0), slightly higher c and c´ ratio (81.0–122.8 *vs* 77.0–96.0, 1.8–2.9 *vs* 1.6–2.2, respectively), and a lower frequency of males (extremely rare vs frequent) [47]. On the other hand, *L*. *indalus* sp. nov. differs from *L*. *unedoi* in shaving lower c and V ratio (81.0–122.8 *vs* 122.0–156.0, 42.0–52.0 *vs* 52.0–58.0; respectively), and slightly higher c´ ratio (1.4–2.0 *vs* 1.8–2.9) [47]. Finally, *L*. *indalus* sp. nov. is molecularly related to *L*. *rubi* [68] from which it can be mainly differentiated morphologically in having a smaller odontostyle and spicules length (53.5–60.5 *vs* 72.0–90.0, 35.0 *vs* 40.0–45.0 μm; respectively), and lower number of ventromedian supplements in male tail (5 *vs* 11–12) [19, 68].

#### Molecular divergence of the new species

D2–D3 region of *L*. *indalus* sp. nov. (KT308852-KT308854) was 91% similar to several *Longidorus* species such as *L*. *closelongatus* (KJ808866), *L*. *pseudoelongatus* (KJ802873) and *L*. *rubi* (JX4455116) (Table 5). *Longidorus indalus* sp. nov. showed a high homogeneity for the D2–D3 region (99% similarity, 3 nucleotides) in the eight sampled populations. However, this homogeneity was lower for the ITS1 sequences (KT308878-KT308879) (98% similar, 23 nucleotides and 17 gaps). Some di- and tri-nucleotides microsatellites, (TA)n and (TGG)n, were found in the population from Lecrín, Granada province (KT308854) contributing to sequence variation. Low homologies in the GenBank were found for ITS1 sequence, the closest species in relation to this marker were *L*. *crassus* (AF511414) and *L*. *grandis* (AF511419), with a similarity of 70% only. The partial 18S of *L*. *indalus* sp. nov. (KT308894-KT308895) closely matched (99% similarity) those for *L*. *closelongatus* (KJ802897), L. *crassus* (AY283158) and *L*. *grandis* (AY283165).

**Table 5:** Identity matrix, percentage (%) of identical residues between (indels included) rDNA sequences amongst *Longidorus* species. Above diagonal D2–D3 expansion segments of 28S rRNA and below diagonal internal transcribed spacer 1 (ITS1) region*.

	*Longidorus* spp.																									
*Longidorus* spp.	1	2	3	4	5	6	7	8	9	10	11	12	13	14	15	16	17	18	19	20	21	22	23	24	25	26
1. *L*. *indalus* sp. nov.*		85	87	88	**91**	87	89	83	85	88	-	87	87	88	-	87	87	88	88	86	84	89	**92**	**90**	87	87
**2. *L*. *macrodorus* sp. nov.**	-		86	88	87	88	86	85	**91**	86	-	87	84	87	-	83	**91**	87	87	88	86	83	88	83	**91**	87
**3. *L*. *onubensis* sp. nov.**	-	62		**91**	88	**91**	87	86	88	85	-	**94**	84	**95**	-	85	89	**93**	**94**	**90**	88	85	88	85	**92**	**95**
**4 *L*. *silvestris* sp. nov.**	-	46	50		89	**98**	88	87	88	87	-	**91**	86	**92**	-	86	**90**	**92**	**92**	**90**	88	84	89	86	**92**	**92**
**5. *L*. *vallensis* sp. nov.**	-	51	51	42		89	**91**	84	87	88	-	88	88	89	-	86	89	89	89	87	85	89	**96**	89	89	88
**6. *L*. *wicuolea* sp. nov.**	-	46	52	88	41		87	86	88	86	-	**91**	86	**91**	-	85	89	**91**	**92**	89	87	84	89	86	**91**	**92**
**7. *L*. *alvegus***	-	46	47	46	39	48		84	86	90	-	87	86	87	-	86	87	88	88	87	85	87	91	88	88	87
**8. *L*. *andalusicus***	-	-	-	-	-	-	-		84	83	-	86	85	87	-	87	87	86	87	89	**94**	84	84	82	87	86
**9. *L*. *baeticus***	-	69	62	46	49	47	44	-		85	-	88	84	89	-	83	89	88	89	88	86	83	87	83	89	88
**10. *L*. *breviannulatus***	53	-	-	-	-	-	-	-	-		-	85	85	86	-	87	88	86	86	86	84	87	**90**	88	87	86
**11. *L*. *crassus***	57	-	-	-	-	-	-	-	-	52		-	-	-	-	-	-	-	-	-	-	-	**-**	-	-	-
**12. *L*. *crataegi***	-	-	-	-	-	-	-	-	-	-	-		84	**96**	-	84	89	**94**	**95**	89	87	84	88	85	**92**	**95**
**13. *L*. *elongatus***	-	56	55	40	55	40	43	-	53	-	-	-		84	-	**95**	85	85	84	85	87	88	89	87	86	85
**14. *L*. *goodeyi***	-	-	-	-	-	-	-	-	-	-	-	-	-		-	85	89	**95**	**96**	**90**	89	85	89	86	**93**	**96**
**15. *L*. *grandis***	57	-	-	-	-	-	-	-	-	52	84	-	-	-		-	-	-	-	-	-	-	-	-	-	-
**16. *L*. *intermedius***	-	55	55	43	53	43	44	-	55	-	-	-	-	-	-		84	84	84	85	87	87	88	86	86	84
**17. *L*. *iuglandis***	-	69	63	46	49	45	45	-	65	-	-	-	-	-	-	55		**90**	**90**	89	88	85	88	86	**91**	**90**
**18. *L*. *lusitanicus***	-	63	79	50	50	50	46	-	62	-	-	-	-	-	-	55	62		**95**	89	87	85	89	85	**92**	**94**
**19. *L*. *magnus***	-	64	85	50	51	51	48	-	64	-	-	-	-	-	-	56	65	80		89	88	85	89	85	**93**	**97**
**20. *L*. *oleae***	-	60	58	42	47	43	41	-	58	-	-	-	-	-	-	52	58	57	60		**91**	85	88	84	**91**	89
**21. *L*. *orientalis***	-	66	62	44	48	45	42	-	62	-	-	-	-	-	-	53	68	61	63	64		87	86	84	**90**	88
**22. *L*. *profundorum***	51	-	-	-	-	-	-	-	-	53	53	-	-	-	53	-	-	-	-	-	-		**90**	88	86	85
**23. *L*. *rubi***	-	51	50	38	66	37	39	-	51	-	-	-	-	-	-	48	49	49	50	46	48	-		**90**	89	88
**24. *L*. *sturhani***	56		-	-	-	-	-	-	-	57	60	-	-	-	60	-	-	-	-	-	-	58	-		87	85
**25. *L*. *vineacola***	-	63	69	51	51	51	49	-	62	-	-	-	-	-	-	54	65	69	70	58	64	-	50	-		**92**
**26. *L*. *vinearum***		64	84	51	51	52	47		64	-	-	-	-	-	-	55	64	78	**91**	58	62	-	50	-	70	

* Similarity between sequences ≥ 90% are in bold letters.

(-) Sequences not available or comparison not carried out because of low homology between sequences.

**Fig 5:**
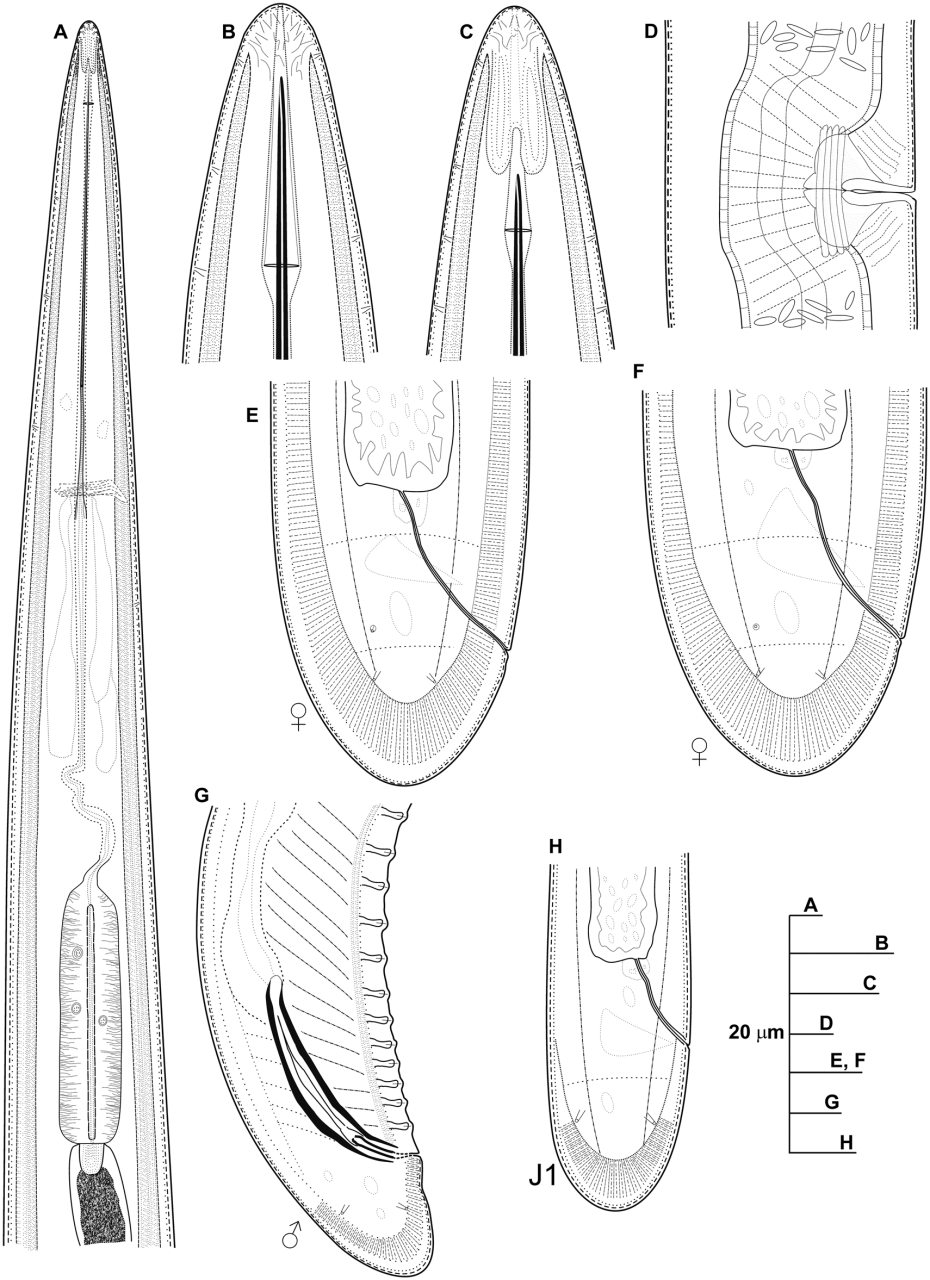
Line drawings of *Longidorus macrodorus* sp. nov., female paratypes, male and first-stage juvenile. A) Pharyngeal region. B, C) Details of lip region. D) Vulval region. E-F) Female tails. G) Male tail. H) First-stage juvenile tail (J1).

**Fig 6:**
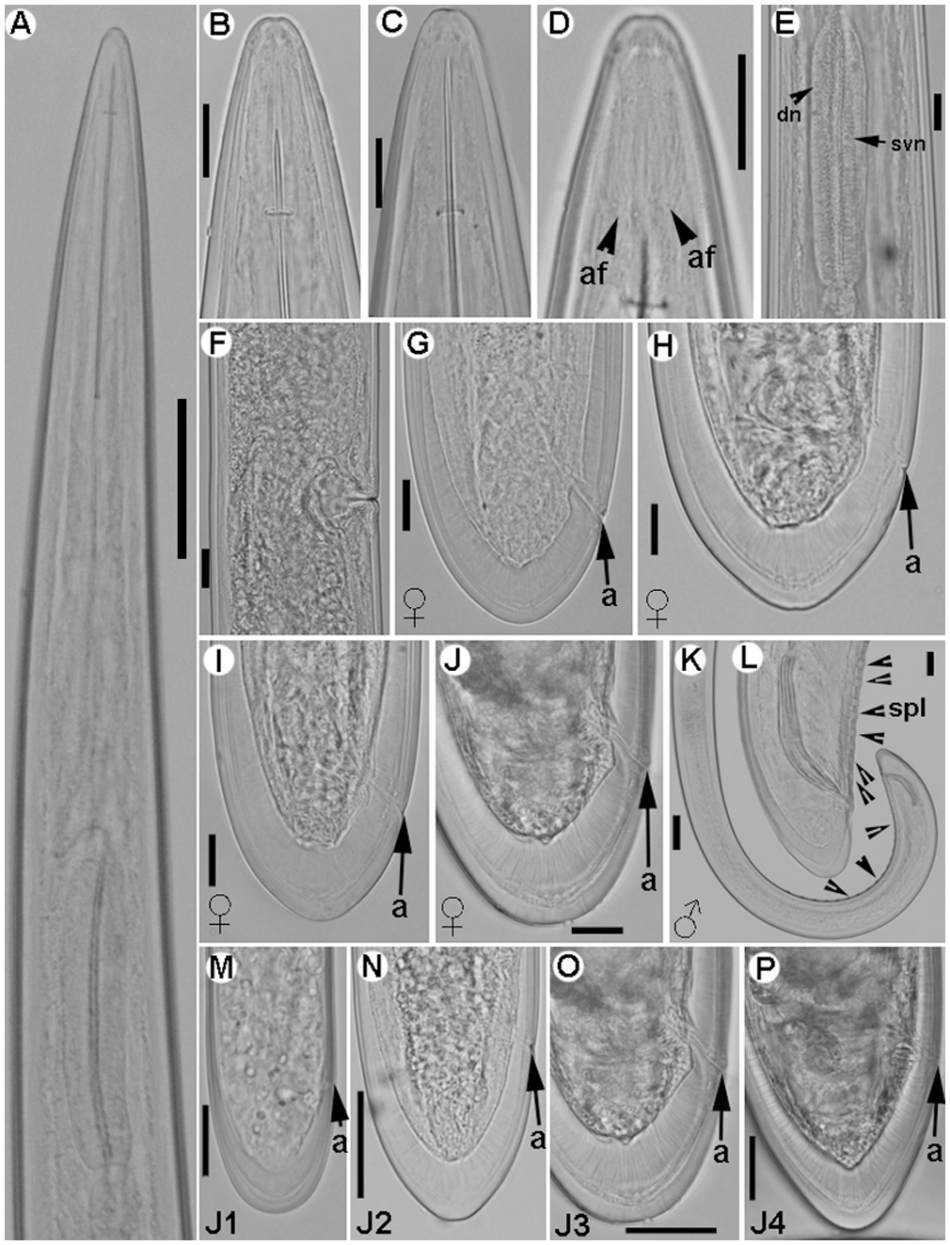
Light micrographs of *Longidorus macrodorus* sp. nov., female paratypes, male and juvenile stages. A) Pharyngeal region. B–D) Female anterior regions. E) Detail of basal bulb. F) Vulval region. H-J) Female tails. K, L) Male tail with detail of spicules. M-P) First-, second-, third-, and fourth-stage juvenile (J1–J4) tails, respectively. Abbreviations: a = anus; af = amphidial fovea; dn = dorsal nucleus; spl = ventromedian supplements; svn = subventral nucleus. Scale bars A, K = 100 μm; B-J, L-P = 20 μm.

***Longidorus macrodorus* Archidona-Yuste, Navas-Cortés, Cantalapiedra-Navarrete, Palomares-Rius & Castillo, sp. nov.** urn:lsid:zoobank.org:act:9A8C0479-3145-4781-B749-027654C7B8E2 Figs 4–6.

#### Holotype

Adult female, collected from the rhizosphere of cultivated olive (*Olea europaea* subsp. *europaea* L.) (38°22'33.9"N, 005°20'46.9"W), at La Grajuela, Córdoba province, Spain; collected by J. Martin Barbarroja, February 19, 2015; mounted in pure glycerine and deposited in the nematode collection at Institute for Sustainable Agriculture (IAS) of Spanish National Research Council (CSIC), Córdoba, Spain (collection number JAO6-01).

#### Paratypes

Female, male and juvenile paratypes extracted from soil samples collected from the same locality as the holotype; mounted in pure glycerine and deposited in the following nematode collections: Institute for Sustainable Agriculture (IAS) of Spanish National Research Council (CSIC), Córdoba, Spain (collection numbers JAO6-05-JAO6-20); one female and one male at Istituto per la Protezione Sostenibile delle Piante (IPSP), Consiglio Nazionale delle Ricerche (CNR), Bari, Italy (JAO6-02); one female and one male at Royal Belgian Institute of Natural Sciences, Brussels, Belgium (RIT839); and one female and one male at USDA Nematode Collection, Beltsville, MD, USA (T-6630p); collected by J. Martin Barbarroja, February 19, 2015.

#### Diagnosis

*Longidorus macrodorus* sp. nov. is a gonochoristic species characterized by a very long body (9.3–10.1 mm), assuming a straight to nearly straight body when heat relaxed; lip region conoid-narrowed continuous with body contour, 8.5–12.0 μm wide; guiding-ring located 45.5–55.0 μm from anterior end; very long odontostyle (183.0–210.0 μm); amphidial fovea pocket-shaped, symmetrically bilobed; vulva almost equatorial; female tail short, bluntly conoid, and bearing between three and four pairs of caudal pores; c’ ratio (0.5–1.0); males as frequently as females with long spicules (90.0–112.0 μm) and 17–25 ventromedian supplements; and specific D2–D3, ITS1 rRNA and partial 18S rRNA sequences (GenBank accession numbers KT308855-KT308856, KT308880-KT308881, and KT308896, respectively). According to the polytomous key Chen *et al*. [64] and the supplement by Loof and Chen [65], the new species has the following code: A7-B1-C45-D1-E2-F54-G12-H1-I2.

#### Etymology

The species name refers to the primarily distinguishing character of the long odontostyle (from Greek *macros* = long, and *dorus* = stylet).

#### Description of taxa. Female

Body very long and rather robust, sharply tapering towards anterior end, usually assuming a body straight or nearly so shape when heat relaxed. Cuticle very finely striated generally but mainly at the posterior extremity, 5.5 ± 0.7 (4.0–7.0) μm thick at mid-body but more thickened at tail tip where it is 13.5 ± 3.0 (8.5–17.0) μm thick, immediately anterior to anus. Lip region conoid-narrowed, anteriorly rounded, and continuous with body contour. Amphidial fovea pocket-shaped slightly symmetrically bilobed with lobes about equal length and extending about 2/3 part of distance between oral aperture and guiding-ring, openings obscure appearing as minute pores, not slit-like. Stylet guiding-ring single, located 5.3 ± 0.6 (4.1–6.0) times lip region diam. from anterior end. Lateral chord 26.2 ± (24.0–30.0) μm wide at mid-body or 20–27% of corresponding body diam. Odontostyle very long and robust straight or slightly arcuate, 3.9 ± 0.3 (3.4–4.0) times as long as distance between anterior end to guiding-ring, odontophore about 2/3 part of the odontostyle length, weakly developed with slightly enlarged at the base. Nerve ring encircling cylindrical part of pharynx at odontophore base, located 271.5 ± 10.3 (252.5–288.0) μm from anterior end. Anterior slender part of pharynx usually coiled in its posterior region. Basal bulb long and cylindrical, 182.2 ± 9.3 (166.0–197.0) μm long or *ca* one-fourth of neck length, and 36.5 ± 3.7 (28.0–45.0) μm in diam. Glandularium 156.9 ± 8.8 (144.5–172.0) μm long. Normal arrangement of pharyngeal glands [64, 65]: nuclei of the dorsal (DN) and subventral (SVN) glands situated at 26.2 ± 4.0 (21.0–33.0)% and 51.1 ± 3.1 (45.7–55.0)% of the distance from anterior end of pharyngeal bulb, respectively. Dorsal gland nucleus (DN) slightly larger than nuclei of two SVN (4.0–6.0 *vs* 3.5–5.0 μm in diam.). Cardia hemispherical, 18.7 ± 3.9 (14.5–25.0) μm long. Reproductive system with both genital branches equally developed, relatively short compared to body length, ranging between 622–1318 μm long, with reflexed ovaries very variable in length. Vulva in form of a transverse slit, located about mid-body, vagina perpendicular to body axis, extending to *ca* 2/3 corresponding body width, surrounded by well-developed muscles. Genital branches equally developed, 8.8 ± 1.6 (6.6–13.0), 8.8 ± 2.0 (6.5–14.0)% of body length, respectively. Uterus short, thick-walled, filled with sperm cells in most female specimens observed; well-developed sphincter between uterus and *pars dilatata oviductus*, usually containing numerous sperm cells too. Ovaries equally developed and very variable in length, 192–545 μm long, both of them with a single row of oocytes. Prerectum variable in length, 2170 ± 559.7 (1427–3045) μm long, and rectum 46.6 ± 7.9 (36.0–56.0) μm long, anus a small rounded slit. Tail short, bluntly conoid, rounded to almost hemispherical, bearing between three and four pairs of caudal pores.

#### Male

Common, as frequently as female. Morphologically similar to female except for genital system, posterior region being more strongly coiled with slightly longer tail. Male genital tract diorchic with testes opposed, containing multiple rows of different stages of spermatogonia. Spicules massive, robust, and curved ventrally; lateral guiding pieces more or less straight or with curved proximal end. Tail convex-conoid, dorsally conoid, ventrally being almost straight with broad blunt terminus and thickened outer cuticular layer. One pair of adanal supplements and 17–25 mid-ventral supplements.

#### Juveniles

Morphometrics obtained from juvenile specimens, and of the relative lengths of body, tail, and functional and replacement odontostyle, confirmed the presence of four juvenile stages (Table 6, Figs 4 and 6; [66, 67]). J1s were characterised by a bluntly rounded to cylindrical tail with a c’ ratio ≥ 1.2 (Table 6), an odontostyle very long, *ca* 120 μm, and shorter distance from anterior end to stylet guiding-ring than that in adult stages.

**Table 6:** Morphometrics of females, males and juvenile stages of *Longidorus macrodorus* sp. nov. from the rhizosphere of cultivated olive at La Grajuela (Córdoba province) southern Spain^a^.

		Paratype		juvenile-stages			
Characters/ratios^b^	Holotype	Females	Males	J1	J2	J3	J4
**n**		19	20	8	6	5	5
**L (mm)**	9.3	9.3 ± 0.50 (8.3–10.1)	9.5 ± 0.77 (8.2–10.6)	3.37 ± 0.40 (2.86–4.02)	4.76 ± 0.39 (4.16–5.16)	6.65 ± 0.96 (5.43–7.75)	7.71 ± 0.45 (70.7–8.18)
**a**	92.0	83.1 ± 5.6 (73.6–92.0)	86.5 ± 9.6 (66.7–101.5)	85.4 ± 4.4 (76.4–89.4	86.0 ± 5.5 (81.2–94.4)	78.3 ± 1.6 (76.4–80.4)	82.2 ± 5.9 (75.6–90.9)
**b**	10.8	13.0 ± 1.5 (10.8–17.0)	13.4 ± 2.4 (9.4–19.0)	8.5 ± 1.5 (6.4–10.5)	10.0 ± 1.0 (8.7–11.0)	10.5 ± 2.3 (7.6–13.4)	12.1 ± 0.7 (11.1–12.8)
**c**	186.8	224.2 ± 40.2 (169.9–323.0)	200.4 ± 21.7 (155.9–246.0)	69.5 ± 17.3 (52.2–96.6)	89.7 ± 4.0 (85.4–94.6)	128.5 ± 26.4 (104.8–172.4)	167.2 ± 11.6 (157.1–184.0)
**c´**	0.7	0.7 ± 0.1 (0.5–1.0)	0.8 ± 0.1 (0.7–1.0)	1.6 ± 0.3 (1.2–2.0)	1.2 ± 0.1 (1.1–1.3)	1.0 ± 0.1 (0.8–1.1)	0.8 ± 0.1 (0.7–0.8)
**V or T**	46.0	48.9 ± 1.8 (46.0–52.0)	33.3 ± 5.4 (24.3–41.6)	-	-	-	-
**Odontostyle**	202.0	196.4 ± 7.7 (183.0–210.0)	197.2 ± 10.0 (181.5–220.0)	121.1 ± 5.2 (113.0–128.0)	130.4 ± 4.4 (123.0–133.5)	157.2 ± 5.5 (153.0–165.0)	179.3 ± 6.0 (173.0–188.0)
**Odontophore**	85.5	75.1 ± 8.7 (60.0–87.0)	73.1 ± 6.4 (59.0–86.5)	55.3 ± 7.3 (46.0–63.5)	51.4 ± 3.7 (48.0–57.5)	62.0 ± 7.4 (54.0–68.5)	66.7 ± 2.4 (63.0–69.5)
**Replacement odontostyle**	-	-	-	134.5 ± 5.1 (123.0–140.5)	154.6 ± 6.3 (146.0–162.0)	174.0 ± 5.1 (170.0–183.0)	199.8 ± 4.6 (192.5–203.0)
**Lip region diam.**	8.5	9.7 ± 1.1 (8.5–12.0)	9.7 ± 1.1 (8.5–12.0)	6.0 ± 0.6 (5.0–6.5)	6.4 ± 0.4 (5.5–7.0)	8.1 ± 1.2 (7.0–10.0)	8.9 ± 1.2 (7.0–10.0)
**Oral aperture-guiding ring**	49.0	50.7 ± 2.8 (45.5–55.0)	51.2 ± 3.4 (45.0–60.0)	32.6 ± 1.9 (30.0–35.5)	37.3 ± 2.0 (34.0–39.5)	43.4 ± 2.7 (39.5–46.5)	47.8 ± 2.8 (45.0–50.5)
**Tail length**	50.0	42.9 ± 6.6 (30.0–54.0)	47.7 ± 4.4 (40.0–55.5)	50.4 ± 9.9 (36.0–64.0)	53.0 ± 2.5 (48.5–54.5)	53.8 ± 14.1 (31.5–66.0)	46.3 ± 3.8 (41.5–52.0)
**Spicules**	-	-	103.0 ± 5.3 (90.0–112.0)	-	-	-	-
**Lateral accessory piece**	-	-	25.7 ± 2.3 (22.5–29.5)	-	-	-	-
**J**	25.0	23.7 ± 2.3 (20.0–28.0)	18.4 ± 1.4 (16.0–22.0)	12.4 ± 1.5 (9.5–13.5)	14.4 ± 1.1 (12.5–15.0)	19.3 ± 0.9 (18.5–20.0)	19.2 ± 2.2 (17.5–23.0)

^a^ Measurements are in μm (except for L) and in the form: mean ± standard deviation (range).

^b^ Abbreviations as defined in Jairajpuri & Ahmad [44]. a, body length/maximum body width; b, body length/pharyngeal length; c, body length/tail length; c', tail length/body width at anus; V (distance from anterior end to vulva/body length) x 100; T (distance from cloacal aperture to anterior end of testis/body length) x 100; J (hyaline tail region length).

#### Measurements, morphology and distribution

**Fig 7:**
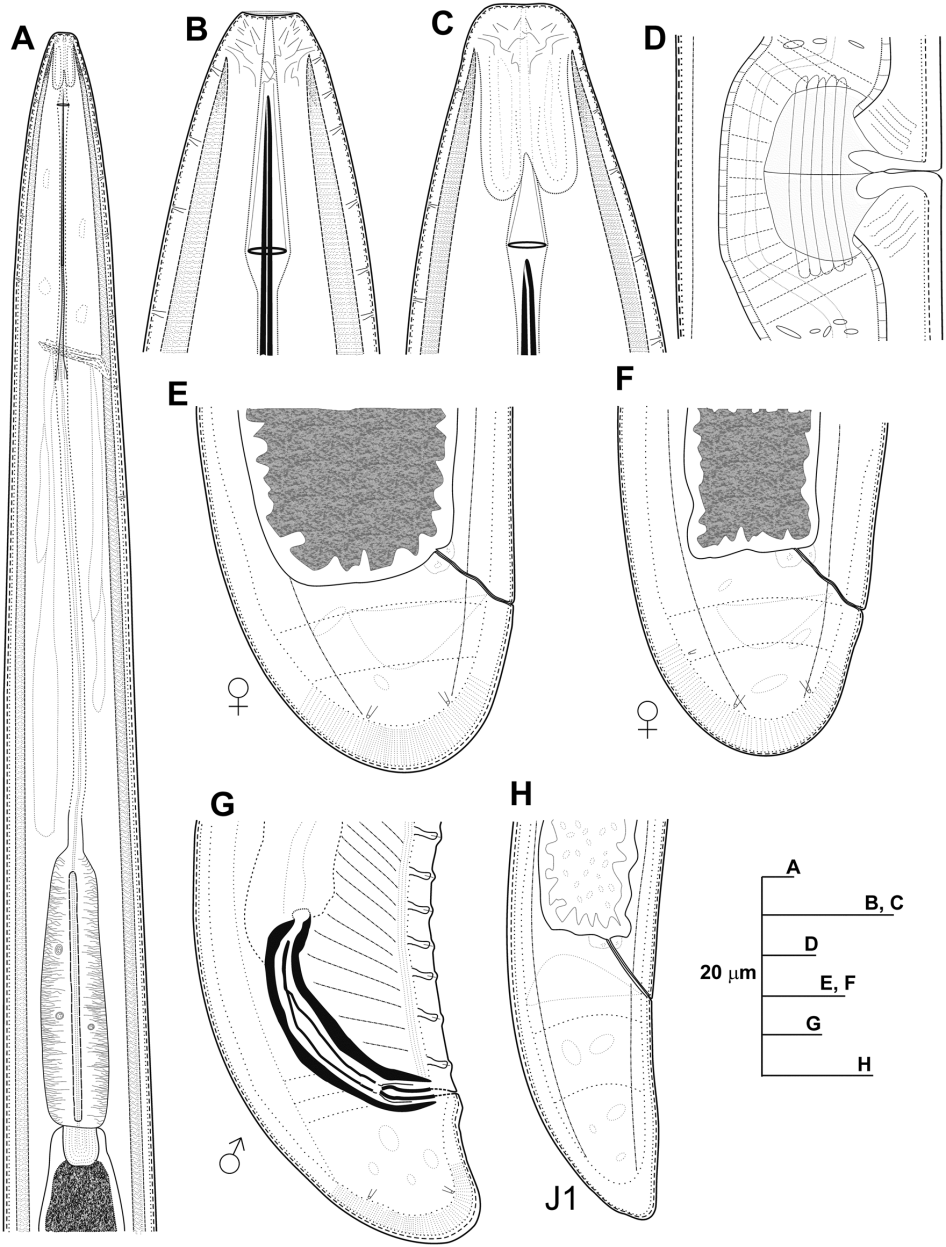
Line drawings of *Longidorus onubensis* sp. nov., female paratypes, male and first-stage juvenile. A) Pharyngeal region. B, C) Details of lip region. D) Vulval region. E, F) Female tail. G) Male tail. H) First-stage juvenile tail (J1).

Morphometric variability is described in Table 6, and morphological traits in Figs 4–7. *Longidorus macrodorus* sp. nov. was only found in the type locality in the rhizosphere of cultivated olive (Table 1, Fig 1).

#### Relationships

*L*. *macrodorus* sp. nov. can be differentiated from all known species of the genus by a combination of characters, but particularly by its stylet and odontostyle length (252–288, 183–210 μm, respectively), the longest in the genus. Nonetheless, according to this morphometric character, included on matrix code A (odontostyle length) [64, 65], *L*. *macrodorus* sp. nov. groups with *L*. *ishigakiensis* Hirata, 2002 [69] and *L*. *tarjani* Siddiqi, 1962 [70]. From *L*. *ishigakiensis* it differs mainly in having a longer body and odontostyle length (8.3–10.1 *vs* 5.3–6.9 mm, 183–210 *vs* 158–181 μm; respectively), lower a and c´ ratios (73.6–92.0 *vs* 106.0–130.0, 0.5–1.0 *vs* 1.0–1.2; respectively), higher c ratio (169.9–323.0 *vs* 133.0–169.0), amphidial pouch shape (symmetrically bilobed *vs* not bilobed, matrix code E2 *vs* E1), and presence *vs* absence of males. From *L*. *tarjani* the new species differs mainly by having a longer body and odontostyle length (8.3–10.1 *vs* 6.0–6.8 mm, 183–210 *vs* 178–182 μm; respectively), higher c ratio (169.9–323 vs 113–130), and lip region shape (rounded continuous *vs* set off from body contour, matrix code D1 *vs* D2). In addition, *L*. *macrodorus* sp. nov. is molecularly related to *L*. *baeticus* Gutiérrez-Gutiérrez, Cantalapiedra-Navarrete, Montes-Borrego, Palomares-Rius & Castillo, 2013 [19] from which it can be mainly differentiated by a slightly longer body length (8.3–10.1 *vs* 6.5–9.4 μm), a longer odontostyle length (183.0–210.0 *vs* 111.0–133.0 μm), and slightly higher c ratio (169.9–323.0 *vs* 180.0–286.2) [19].

#### Molecular divergence of the new species

The sequence divergences between *L*. *macrodorus* sp. nov. and other congeneric species were significant, D2–D3 sequences (KT308855-KT308856) were 91% similar to *L*. *baeticus* (JX445106-JX445107), *L*. *iuglandis* (JX445105) and *L*. *fasciatus* (JX445108) (Table 5). No intraspecific variation for the D2–D3 segments was detected between the two studied samples. ITS1 sequences (KT308880-KT308881) region also agree with results obtained from D2–D3, these sequences were 75% similar to *L*. *baeticus* (JX445093), *L*. *fasciatus* (JX445097) and *L*. *iuglandis* (JX445099). Similarity values for the partial 18S of *L*. *macrodorus* sp. nov. sequence (KT308896) with those deposited in GenBank were high and matched closely with several sequences such, as *L*.*vineacola* (AY283169) and *L*. *elongatus* (EU503141).

**Fig 8:**
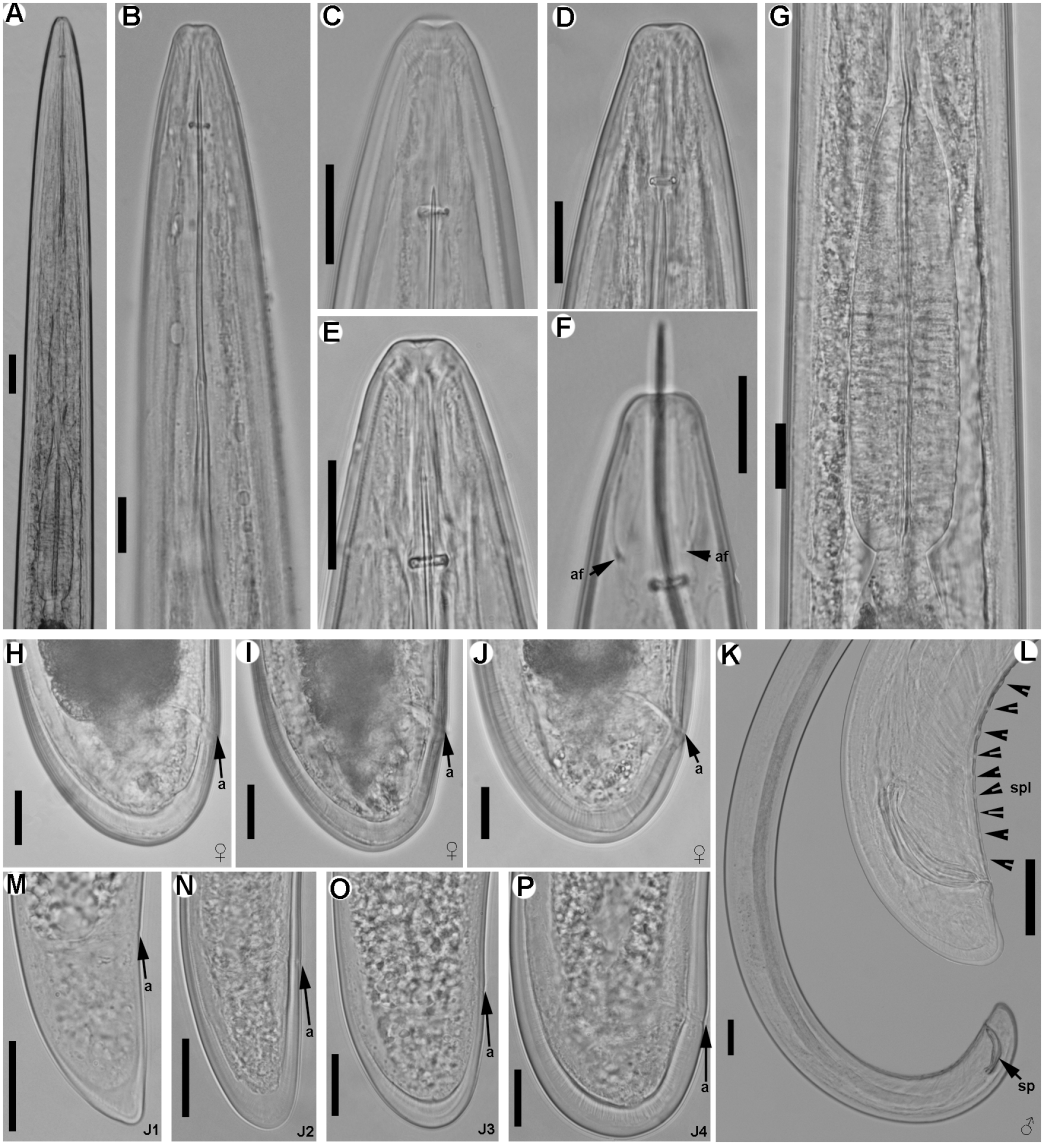
Light micrographs of *Longidorus onubensis* sp. nov., female paratypes, male and juvenile stages. A) Pharyngeal region. B) Female anterior region. C-F) Female lip regions. G) Detail of basal bulb. H-J) Female tails. K, L) Male tail with detail of spicules. M-P) First-, second-, third-, and fourth-stage juvenile (J1–J4) tails, respectively. Abbreviations: a = anus; af = amphidial fovea; spl = ventromedian supplements. Scale bars A-J, M-P = 20 μm; K, L = 10 μm.

***Longidorus onubensis* Archidona-Yuste, Navas-Cortés, Cantalapiedra-Navarrete, Palomares-Rius & Castillo, sp. nov.** urn:lsid:zoobank.org:act:A9BE98FF-58A2-4BA4-8DFF-309D31C7D64F Figs 4, 7 and 8.

#### Holotype

Adult female, collected from the rhizosphere of cultivated olive (*Olea europaea* subsp. *europaea* L.) (37°21'49.3"N, 006°39'56.8"W), Niebla, Huelva province, Spain; collected by J. Martin Barbarroja, January 21, 2012; mounted in pure glycerine and deposited in the nematode collection at Institute for Sustainable Agriculture (IAS) of Spanish National Research Council (CSIC), Córdoba, Spain (collection number ST5-13).

#### Paratypes

Female, male and juvenile paratypes extracted from soil samples collected from the same locality as the holotype; mounted in pure glycerine and deposited in the following nematode collections: Institute for Sustainable Agriculture (IAS) of Spanish National Research Council (CSIC), Córdoba, Spain (collection numbers ST5-02-ST5-12); one female and one male at Royal Belgian Institute of Natural Sciences, Brussels, Belgium (RIT842); and one female and male at USDA Nematode Collection, Beltsville, MD, USA (T-6631p); collected by J. Martin Barbarroja, January 21, 2012.

#### Diagnosis

*Longidorus onubensis* sp. nov. is a gonochoristic species characterized by a long and rather body (7.4–9.5mm), assuming an open C-shaped when heat relaxed; lip region broadly rounded to truncate, continuous or separated from body contour by slight depression, 14.0–16.5 μm wide; guiding-ring located 31–44μm from anterior end; long odontostyle (103–121 μm); amphidial fovea pocket-shaped with lobes of about equal length; vulva almost equatorial; female tail very short, broadly conoid to hemispherical, and bearing two or three pairs of caudal pores; c’ ratio (0.6–0.8); males frequent with long spicules (92–98 μm) and 14–16 ventromedian supplements; and specific D2–D3, ITS1 rRNA and partial 18S rRNA sequences (GenBank accession numbers KT308857-KT308858, KT308882-KT308883, and KT308897, respectively). According to the polytomous key Chen *et al*. [64] and the supplement by Loof and Chen [65], the new species has the following code (codes in parentheses are exceptions): A4-B2(3)-C34-D23-E2-F45-G2-H1-I2.

#### Etymology

The species epithet refers to ‘*Onuba*’, the Roman name of the province of Huelva, where the type specimens were collected.

#### Description of taxa. Female

Body long and rather robust, slightly tapering towards anterior end, usually assuming an open C-shaped when heat relaxed, almost straight anteriorly and more curved behind the vulva to single spirals. Cuticle appearing smooth, 4.5 ± 0.8 (3.5–6.0) μm thick, 11.1 ± 2.3 (8.5–13.5) μm thick at tail tip, and marked by very fine superficial transverse striate mainly in tail region. Lip region broadly rounded frontally and more so laterally, separated from body contour by slight depression. However, lip region truncate, slightly concave anteriorly and continuous with body contour shape, observed in some female specimens. Amphidial fovea pocket-shaped symmetrically bilobed, with lobes of about equal length, occupying more of 2/3 part of distance between oral aperture and guiding-ring. Labial papillae prominent. Stylet guiding-ring single, located 2.5 ± 0.3 (2.1–3.0) times lip region diam. from anterior end. Lateral chord *ca* 25 μm wide at mid-body or one-fourth of corresponding body diam. Odontostyle moderate long and robust, usually straight, 1.9 ± 0.1 (1.6–2.29) times as long as odontophore; odontophore weakly developed, posterior slightly enlarged with rather weak basal swellings. Nerve ring encircling cylindrical part of pharynx, 11.3 ± 0.6 (10.2–12.3) times body width at lip region far from anterior end. Anterior slender part of pharynx usually coiled in its posterior region. Basal bulb long and cylindrical, 149.7 ± 11.2 (135.0–173.0) μm long or *ca* one-third of neck length, 32.0 ± 3.7 (27.0–38.5) μm diam. Dorsal pharyngeal gland nucleus (DN) and ventro-sublateral pair of nuclei (SN) situated slightly posterior to normal arrangement of pharyngeal glands [64, 65], 34.8 ± 4.2 (30.3–39.5)%, and 56.7 ± 7.1 (52.0–69.0)% of distance from anterior end of pharyngeal bulb, respectively. Dorsal gland nucleus (DN) slightly larger than nuclei of two SVN (4.0–4.5 *vs* 3.5–4.0 μm in diam.). Glandularium 129.6 ± 11.8 (115.0–153.0) μm long. Cardia conoid-rounded, 12.3 ± 1.0 (11.5–13.5) μm long. Reproductive system with both genital branches equally developed, ranging between 456–989 μm long, with reflexed ovaries variable in length. *Pars dilatata oviductus* and uterus of about equal length, separated by a very strong and muscularised sphincter, on the external wall of which very cell body protrusions are present. Genital branches about equally developed, 7.4 ± 1.2 (6.1–9.4), 8.2 ± 1.5 (5.8–10.4)% of body length, respectively. Uterus wide and thick-walled, filled with little sperm cells in most female specimens observed. Ovaries equally developed 147–233 μm long, both of them with a single row of oocytes. Vulva in form of a transverse slit, approximately equatorial; vagina perpendicular to body axis, 42.0 ± 6.7 (30.0–50.5) μm long, or *ca* 42% of corresponding body width, surrounded by well-developed muscles. Prerectum very variable in length, 887.0 ± 331.1 (467.0–1155.0) μm long, and rectum 39.6 ± 4.4 (34.0–43.5) μm long, anus a small rounded slit. Tail very short, broadly conoid to hemispherical, with rounded terminus, bearing two or three pairs of caudal pores.

#### Male

Common, but less frequent (40%) than female. Morphologically similar to female except for genital system, but with posterior region slightly curved ventrally and longer tail. Male genital tract diorchic with testes opposed, containing multiple rows of different stages of spermatogonia. Tail rounded, dorsally convex conoid, ventrally slightly concave with broad blunt terminus and thickened outer cuticular layer. Spicules arcuate, robust, *ca* 2 times longer than tail length, lateral guiding pieces more or less straight or with curved proximal end. One pair of adanal supplements and 14–16 midventral supplements.

#### Juveniles

Morphologically similar to adults, but smaller. All four juvenile stages were found, being distinguishable by relative lengths of body and functional and replacement odontostyle (Table 7, Figs 4, 7 and 8; [66, 67]). J1s were characterised by a conoid-rounded tail, curved dorsally and slightly concave ventrally with a dorsal-ventral depression at hyaline region level, c’ ratio ≥ 1.5 (Table 7), an odontostyle length *ca* 58 μm, and shorter distance from anterior end to stylet guiding-ring than that in adult stages.

**Table 7:** Morphometrics of females, males and juvenile stages of *Longidorus onubensis* sp. nov. from the rhizosphere of cultivated olive at Niebla (Huelva province) southern Spain^a^.

		Paratype		juvenile-stages			
Characters/ratios^b^	Holotype	Females	Males	J1	J2	J3	J4
**n**		14	6	6	5	5	5
**L (mm)**	7.4	8.7 ± 0.68 (7.4–9.6)	8.3 ± 0.80 (7.0–9.3)	2.18 ± 0.20 (1.94–2.42)	2.60 ± 0.20 (2.36–2.86)	4.07 ± 0.90 (3.92–4.16)	5.84 ± 0.92 (4.86–7.16)
**a**	75.9	88.6 ± 8.4 (75.9–107.5)	96.3 ± 16.8 (69.8–118.9)	63.8 ± 2.6 (60.5–67.9)	62.5 ± 3.6 (58.1–65.8)	64.5 ± 8.0 (50.9–71.1)	77.3 ± 11.9 (62.0–91.9)
**b**	14.3	17.3 ± 2.0 (14.2–20.9)	17.2 ± 2.6 (14.6–21.2)	9.5 ± 2.2 (6.1–12.1)	9.4 ± 1.8 (7.3–11.6)	12.1 ± 0.9 (10.7–13.0)	12.5 ± 2.5 (11.0–16.7)
**c**	230.1	211.7 ± 22.9 (184.4–272.7)	178.5 ± 19.5 (150.0–206.7)	56.1 ± 9.8 (47.6–75.2)	67.0 ± 7.4 (60.9–76.7)	95.2 ± 15.6 (78.9–121.0)	139.2 ± 24.2 (105.7–172.5)
**c´**	0.6	0.7 ± 0.1 (0.6–0.8)	0.8 ± 0.0 (0.7–0.8)	1.6 ± 0.1 (1.5–1.8)	1.3 ± 0.1 (1.1–1.5)	1.0 ± 0.1 (0.8–1.1)	0.8 ± 0.1 (0.7–0.8)
**V**	51.0	49.7 ± 1.8 (46.0–52.0)	-	-	-	-	-
**Odontostyle**	121.0	112.9 ± 6.2 (103.0–121.0)	114.7 ± 8.2 (105.0–123.5)	57.7 ± 1.8 (55.5–59.5)	66.6 ± 5.2 (59.0–71.0)	79.9 ± 1.1 (78.5–81.0)	98.8 ± 11.0 (85.5–114.5)
**Odontophore**	58.0	59.4 ± 5.5 (54.5–72.0)	56.6 ± 7.1 (47.0–65.0)	34.6 ± 4.8 (30.0–41.0)	47.5 ± 8.0 (38.5–54.0)	42.1 ± 7.5 (34.0–53.0)	48.6 ± 6.0 (40.5–54.5)
**Replacement odontostyle**	-	-	-	68.4 ± 5.1 (63.5–77.5)	80.6 ± 2.8 (77.0–84.5)	89.7 ± 2.1 (88.0–93.0)	114.4 ± 10.1 (104.5–129.5)
**Lip region diam.**	15.5	15.4 ± 0.8 (14.0–16.5)	15.3 ± 0.7 (14.5–16.5)	8.0 ± 0.5 (7.5–8.5)	9.3 ± 1.0 (8.5–10.5)	10.6 ± 0.8 (10.0–12.0)	12.4 ± 1.2 (11.5–14.0)
**Oral aperture-guiding ring**	40.5	33.7 ± 3.1 (28.5–38.5)	40.0 ± 3.1 (35.5–43.5)	21.1 ± 1.5 (19.5–23.0)	23.0 ± 0.8 (22.0–24.0)	27.0 ± 1.3 (25.5–29.0)	33.2 ± 1.6 (31.0–35.5)
**Tail length**	32.0	41.4 ± 4.7 (32.0–48.5)	46.7 ± 3.0 (42.0–51.0)	39.5 ± 5.9 (31.0–45.5)	39.2 ± 4.9 (34.5–47.0)	43.6 ± 6.8 (32.5–51.0)	42.1 ± 2.7 (39.5–46.0)
**Spicules**	-	-	94.3 ± 2.1 (92.0–98.0)	-	-	-	-
**Lateral accessory piece**	-	-	23.6 ± 2.1 (21.0–26.5)	-	-	-	-
**J**	12.5	13.8 ± 1.3 (12.0–16.0)	14.9 ± 0.9 (14.0–16.5)	9.3 ± 1.4 (7.0–10.5)	7.0 ± 1.0 (6.5–8.5)	8.8 ± 1.0 (7.5–10.0)	10.6 ± 0.7 (10.0–11.5)

^a^ Measurements are in μm (except for L) and in the form: mean ± standard deviation (range).

^b^ Abbreviations as defined in Jairajpuri & Ahmad [44]. a, body length/maximum body width; b, body length/pharyngeal length; c, body length/tail length; c', tail length/body width at anus; V (distance from anterior end to vulva/body length) x 100; T (distance from cloacal aperture to anterior end of testis/body length) x 100; J (hyaline tail region length).

#### Measurements, morphology and distribution

Morphometric variability is described in Table 7 and morphological traits in Figs 4, 7 and 8. *Longidorus onubensis* sp. nov. was only found in the type locality from the rhizosphere of cultivated olive (Table 1, Fig 1).

#### Relationships

According to the polytomous key by Chen *et al*. [64] and the supplement by Loof and Chen [65], and on the basis of sorting on matrix codes A (odontostyle length), B (lip region width), D (lip region shape), F (body length), and H (tail shape), *L*. *onubensis* sp. nov. is closed to *L*. *goodeyi* Hooper, 1961 [71], *L*. *iuglandis* Roca, Lamberti & Agostinelli, 1984 [72], *L*. *oleae* and *L*. *vinearum*. From *L*. *goodeyi* it differs mainly in having a longer body and odontostyle length (7.4–9.6 *vs* 5.6–7.7 mm, 103–121 *vs* 96–109 μm; respectively), higher c ratio (184.4–272.7 vs 99.0–188.0), and presence vs absence of males) [38, 71]. On the other hand, from *L*. *iuglandis* it differs mainly by a slightly longer body length (7.4–9.6 vs 5.4–8.3 mm) [19, 72]. From *L*. *oleae* it differs mainly by a smaller distance between guiding-ring from anterior end (28.5–38.5 *vs* 36.0–46.0 μm), a slightly narrower lip region width (14.0–16.5 vs 14.5–21.0 μm), and amphidial fovea shape (symmetrically *vs* asymmetrically bilobed) (S1 Table; [19]). Finally, *L*. *onubensis* sp. nov. differs mainly from *L*. *vinearum* in having a slightly smaller distance between guiding-ring from anterior end and spicules length (28.5–38.5 *vs* 32.5–47.0 μm, 92.0–98.0 vs 100.0–136.5 μm; respectively), and narrower lip region width (14.0–16.5 *vs* 18.0–28.0 μm) (S2 Table; [46, 73]).

#### Molecular divergence of the new species

D2–D3 region of *L*. *onubensis* sp. nov. (KT308857-KT308858) was 95 and 94% similar to *L*. *goodeyi* (AY601581) and *L*. *vinearum* (KT308874-KT308877), respectively (Table 5). Intraspecific variation of D2–D3 segments detected amongst the studied individuals, consisted of one nucleotide and no indels (99% similarity). Similarly, intraspecific variation of the ITS1 for these sequences (KT308882-KT308883) was low, 99% similarity with 0 nucleotides differences and 3 gaps. ITS1 also showed some similarity (85%) with *L*. *vinearum* (KT308892-KT308893). Finally, the partial 18S of *L*. *onubensis* sp. nov. (KT308897) showed a high level of similarity (99%) with *L*. *oleae* (JX445119), *L*.*vineacola* (JX445123), and *L*. *andalusicus* (JX445118).

**Fig 9:**
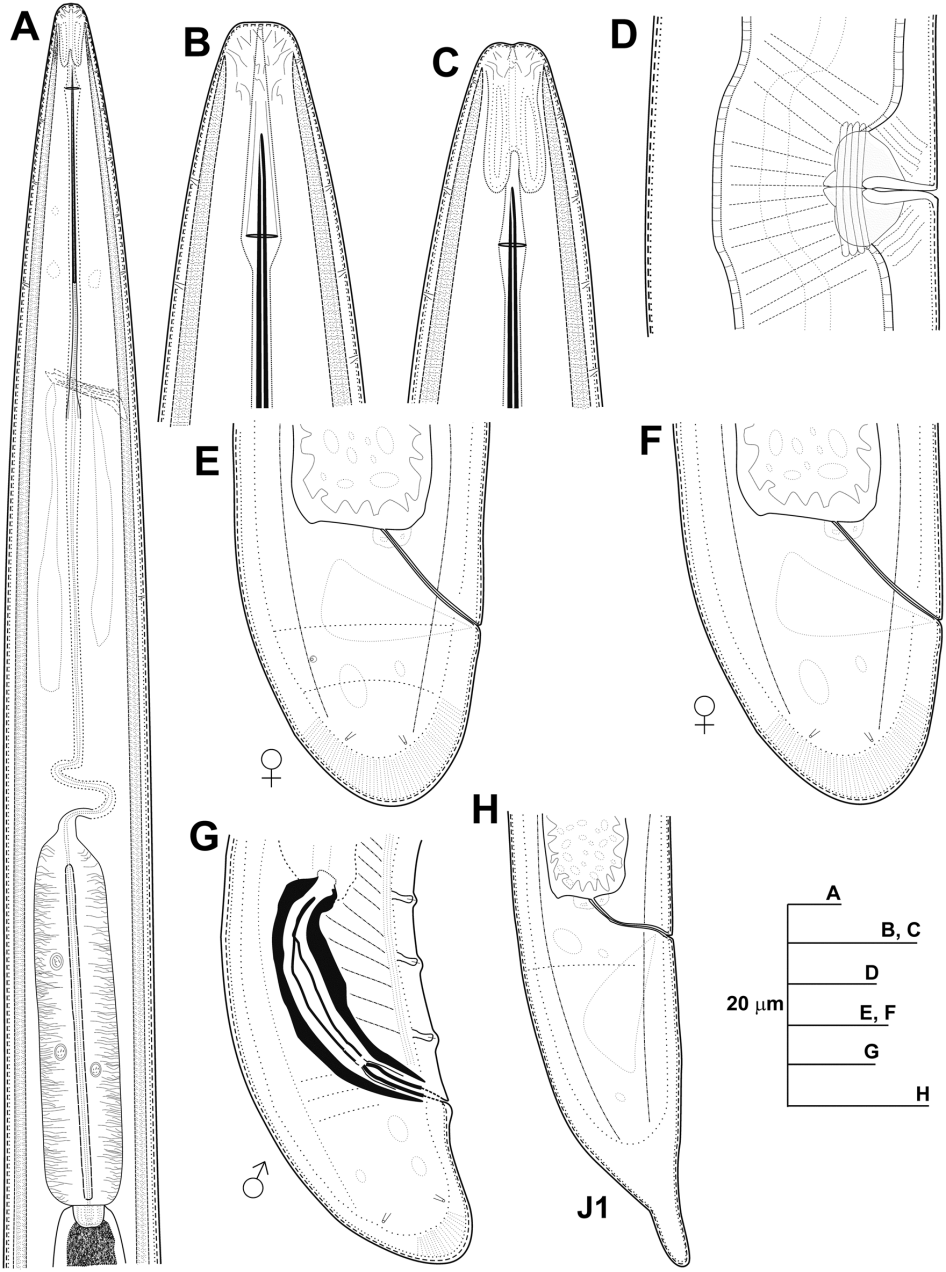
Line drawings of *Longidorus silvestris* sp. nov., female paratypes, male and juvenile stages. A) Pharyngeal region. B, C) Details of lip region. D) Vulval region. E, F) Female tails. G) Male tail. H) First-stage juvenile tail (J1).

**Fig 10:**
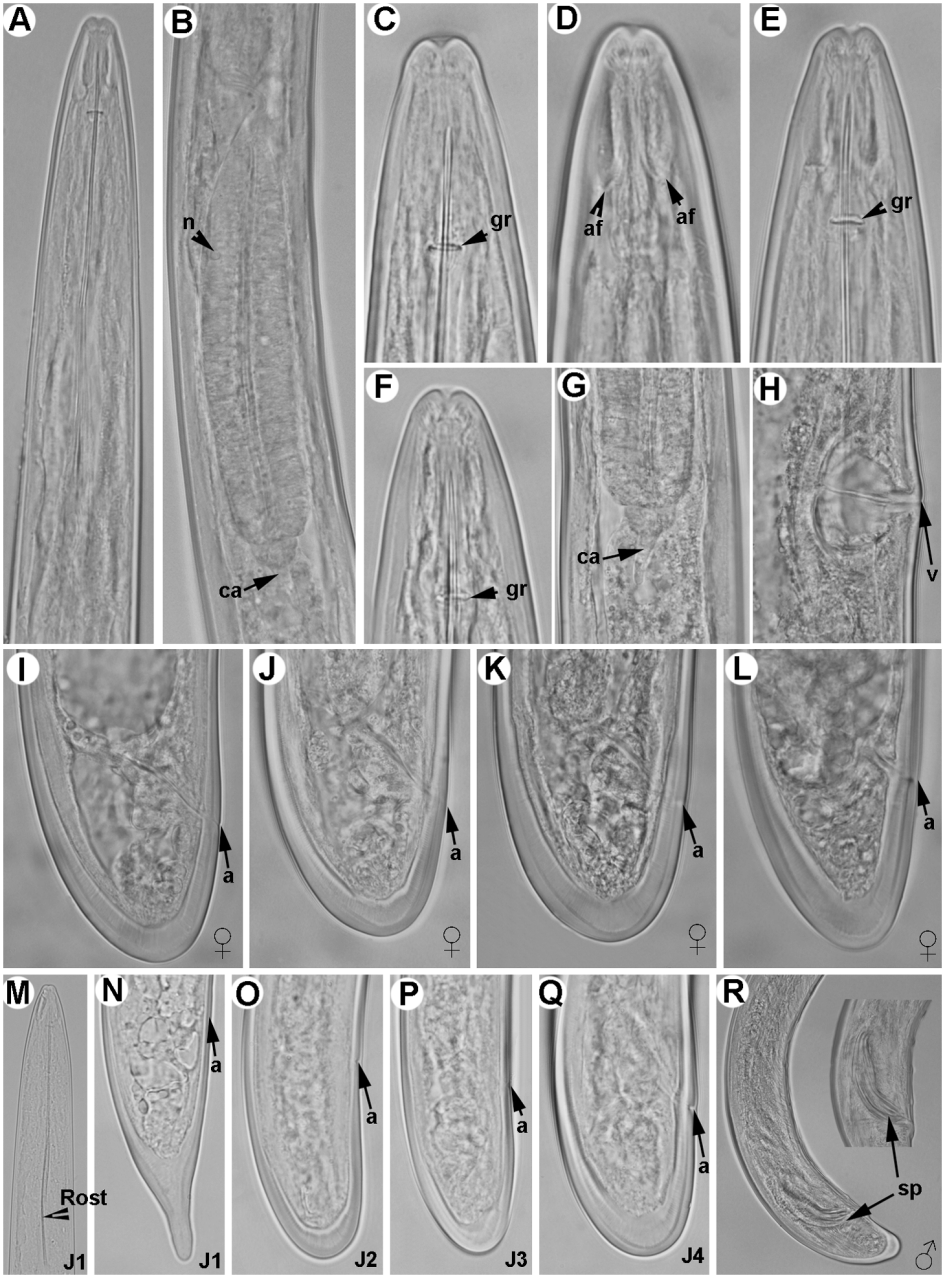
Light micrographs of *Longidorus silvestris* sp. nov., female paratypes, male and juvenile stages. A) Female anterior region. B) Detail of basal bulb. C-F) Female lip regions. G) Detail of pharyngeal-intestinal junction. H) Vulval region. I-L) Female tails. M) First-stage juvenile lip region showing replacement odontostyle inside odontophore. N-Q) First-, second-, third-, and fourth-stage juvenile (J1–J4) tails, respectively. R, S) Male tail and detail of spicules. Abbreviations: a = anus; af = amphidial fovea; ca = cardias; gr = guiding-ring; n = nucleus; Rost = replacement odontostyle; sp = spicules. Scale bars = 20 μm.

***Longidorus silvestris* Archidona-Yuste, Navas-Cortés, Cantalapiedra-Navarrete, Palomares-Rius & Castillo, sp. nov.** urn:lsid:zoobank.org:pub:C8230A9D-FD45-4AA4-9ABF-8445E8001CCC Figs 4, 9 and 10

#### Holotype

Adult female, collected from the rhizosphere of wild olive (*Olea europaea* subsp. *silvestris* (Miller) Lehr) (36°06'34.4"N latitude, 5°42'39.5"W longitude), Tarifa, Cádiz province, Spain; collected by P. Castillo, May 1, 2012; mounted in pure glycerine and deposited in the nematode collection at Institute for Sustainable Agriculture (IAS) of Spanish National Research Council (CSIC), Córdoba, Spain (collection number AR27-19).

#### Paratypes

Female, male and juvenile paratypes extracted from soil samples collected from the same locality as the holotype; mounted in pure glycerine and deposited in the following nematode collections: Institute for Sustainable Agriculture (IAS) of Spanish National Research Council (CSIC), Córdoba, Spain (collection numbers AR27-01-AR27-15); one female at Istituto per la Protezione Sostenibile delle Piante (IPSP), Consiglio Nazionale delle Ricerche (CNR), Bari, Italy (AR27-16); one female at Royal Belgian Institute of Natural Sciences, Brussels, Belgium (RIT838); and two females at USDA Nematode Collection, Beltsville, MD, USA (T-6632p); collected by P. Castillo, May 1, 2012.

#### Diagnosis

*Longidorus silvestris* sp. nov. is a gonochoristic species characterized by a long and robust body (5.0–7.0 mm), assuming an open C-shaped when heat relaxed; lip region narrow, conoid-rounded, continuous with body contour, 9.5–11.5 μm wide; guiding-ring located 30.5–35.5 μm from anterior end; odontostyle 76.0–89.0 μm long; amphidial fovea pocket-shaped symmetrically bilobed; vulva equatorial; female tail short, hemispherical to blunty-conoid, bearing two or three pairs of caudal pores and c’ ratio (0.7–1.0); male extremely rare, only one male found, with spicules 69 μm long and 11 ventromedian supplements; and specific D2–D3, ITS1 rRNA and partial 18S rRNA sequences (GenBank accession numbers KT308859-KT308860, KT308884, and KT308898, respectively). According to the polytomous key Chen *et al*. [64] and the supplement by Loof and Chen [65], the new species has the following code (codes in parentheses are exceptions): A3(2)-B1-C3-D1-E2-F3-G2(1)-H1-I12.

#### Etymology

The species name refers to the habitat (*silvestris*, *silvestre* = sylvan, living in the wild forest), where the type specimens were collected.

#### Description of taxa. Female

Body robust, slightly tapering towards anterior end, usually assuming an open C-shaped when heat relaxed. Cuticle appears smooth, 3.2 ± 0.2 (3.0–3.5) μm thick, 13.6 ± 4.3 (8.0–19.0) μm thick at tail tip, and marked by very fine superficial transverse striae mainly in tail region. Lip region narrow, conoid-rounded, continuous with body contour. Amphidial fovea pocket-shaped symmetrically bilobed, extending about 3/4 part of anterior end-guiding ring distance. Labial papillae prominent. Guiding system with well-developed compensation sacs. Stylet guiding-ring single, located at 32.3 ± 1.6 (30.0–35.5) μm from anterior end. Odontostyle moderately long and narrow, 1.6 ± 0.2 (1.4–2.0) times as long as odontophore, straight or slightly arcuate; odontophore weakly developed, with rather weak basal swellings. Nerve ring encircling narrower part of pharynx. Pharynx consisting of an anterior slender narrow part 307–572 μm long, extending to a cylindrical, terminal pharyngeal bulb, well demarcated anteriorly, 103–155 μm long and occupying *ca* 22–40% of total pharyngeal length. Glandularium 110.6 ± 13.2 (92.0–136.55) μm long. Dorsal pharyngeal gland nucleus (DN) located at 35.3 ± 4.4 (28.4–42.2)%, nucleolus being slightly larger (2.0–4.5 *vs* 2.5–3.5 μm) than nucleoli of two ventrosublateral nuclei (SVN) situated at 57.8 ± 4.0 (53.2–64.4)% of distance from anterior end of pharyngeal bulb, respectively. Cardia well-developed, hemispherical, 17.3 ± 2.6 (15.0–21.0) μm long. Reproductive system with both genital branches equally developed, 7.9 ± 0.8 (6.1–9.3), 7.9 ± 0.9 (6.4–9.9)% of body length, respectively. Ovaries reflexed, variable in length, *ca* 72–110 μm long. Vulva in form of a transverse slit, located about mid-body, vagina perpendicular to body axis, 24.5 ± 2.6 (18.5–32.0) μm long, or 28–48% of corresponding max body width, surrounded by well-developed muscles. Uteri 456 ± 52.7 (372–578) μm long, without sperm cells in the female specimens examined and well-developed sphincter between uterus and oviduct. Prerectum short and variable in length, 414.3 ± 79.9 (266.0–489.0) μm long or *ca* 5–9% of body length. Rectum 31.4 ± 4.1 (26.5–37.0) μm long. Tail short, hemispherical to blunty-conoid shape, bearing two or three pairs of caudal pores.

#### Male

Extremely rare, only one male specimen was found. Morphologically similar to female except for genital system and posterior region slightly curved ventrally Tail convex-conoid, ventrally slightly concave with broad blunt terminus and the thickened outer cuticular layer. Male genital tract diorchic with test opposed, containing multiple rows of different stages of spermatogonia. Spicules arcuate, robust, about 2 times longer than tail length, lateral guiding pieces more or less straight. One pair of adanal supplements preceded by a row of 10 ventromedian supplements.

#### Juveniles

Morphologically similar to adults in most respects except for size and development reproductive system. All juvenile developmental stages were detected and distinguished by relative lengths of body and functional and replacement odontostyle (Table 8, Figs 4 and 10; [66, 67]), and the genital primordium. J1s characterised by a conoid-rounded tail, slightly curved dorsally and dorsal-ventral depression at hyaline region level, subdigitate (Fig 10), with a c´ ratio *ca* 2.5, odontostyle length *ca* 49 μm, and shorter distance from anterior end to stylet guiding-ring than that in adult stages. However, morphology in second-, third- and fourth-stages (except for undeveloped genital structures) similar to that of female, including broadly conoid to hemispherical tail shape with rounded terminus, which becoming progressively shorter and stouter in each moult and shorter distance from anterior end to guiding-ring in each moult (Fig 10).

**Table 8:** Morphometrics of females, males and juvenile stages of *Longidorus silvestris* sp. nov. from the rhizosphere of wild olive at Tarifa (Cádiz province) southern Spain^a^.

		Paratype		juvenile-stages			
Characters/ratios^b^	Holotype	Females	Male	J1	J2	J3	J4
**n**		19	1	7	6	5	5
**L (mm)**	6.0	5.8 ± 0.51 (5.0–7.0)	5.8	1.66 ± 0.27 (1.19–2.03)	2.43 ± 0.24 (2.16–2.84)	3.13 ± 0.52 (2.53–3.78)	4.75 ± 0.51 (4.15–5.39)
**a**	93.5	86.9 ± 7.5 (73.0–101.4)	96.6	62.4 ± 5.4 (56.9–70.4)	73.6 ± 4.8 (67.5–80.0)	76.9 ± 6.1 (69.3–85.4)	91.7 ± 5.6 (86.2–99.4)
**b**	15.0	14.2 ± 1.5 (11.2–16.3)	12.8	7.9 ± 1.5 (5.8–10.2)	8.4 ± 1.2 (6.5–10.0)	9.4 ± 1.8 (7.8–11.8)	11.9 ± 2.1 (10.2–15.3)
**c**	145.2	153.9 ± 12.1 (136.9–182.4)	143.1	35.3 ± 6.6 (50.6–54.8)	57.3 ± 4.2 (50.5–61.0)	82.0 ± 13.5 (67.6–99.6)	120.4 ± 8.2 (110.6–133.0)
**c´**	0.9	0.8 ± 0.1 (0.7–1.0)	0.9	2.5 ± 0.3 (2.0–2.8)	1.7 ± 0.1 (1.6–1.9)	1.3 ± 0.1 (1.2–1.5)	1.0 ± 0.1 (0.9–1.1)
**V**	50	50.1 ± 0.6 (49.0–51.5)	-	-	-	-	-
**Odontostyle**	86.5	82.7 ± 3.4 (76.0–89.0)	83.5	48.7 ± 3.4 (44.0–52.0)	56.1 ± 2.3 (53.0–58.0)	64.3 ± 2.6 (61.5–67.5)	75.8 ± 2.0 (74.0–79.0)
**Odontophore**	49.0	50.9 ± 5.0 (42.0–62.0)	42.0	33.3 ± 2.1 (30.0–36.0)	34.3 ± 1.3 (32.0–36.0)	37.9 ± 3.8 (33.5–43.0)	43.5 ± 3.0 (40.0–48.0)
**Replacement odontostyle**	-	-	-	58.1 ± 4.7 (50.5–65.0)	65.7 ± 2.7 (62.0–70.0)	77.4 ± 3.9 (70.5–80.0)	85.2 ± 3.3 (82.0–89.5)
**Lip region diam.**	10.0	10.2 ± 0.6 (9.5–11.5)	10.5	7.1 ± 0.3 (6.5–7.5)	7.3 ± 0.3 (7.0–7.5)	7.4 ± 0.4 (7.0–8.0)	9.5 ± 0.4 (9.0–10.0)
**Oral aperture-guiding ring**	31.0	32.3 ± 1.6 (30.0–35.5)	33.0	18.6 ± 1.1 (16.5–20.0)	23.0 ± 1.0 (21.5–24.0)	24.5 ± 1.4 (23.0–26.5)	30.4 ± 1.7 (29.5–33.5)
**Tail length**	41.0	37.9 ± 2.3 (33.0–42.0)	40.5	47.5 ± 6.7 (42.0–61.0)	42.6 ± 4.7 (35.5–46.5)	38.1 ± 0.4 (37.5–38.5)	39.4 ± 2.1 (37.5–42.5)
**Spicules**	-	-	69.0	-	-	-	-
**Lateral accessory piece**	-	-	17.0	-	-	-	-

^a^ Measurements are in μm (except for L) and in the form: mean ± standard deviation (range).

^b^ Abbreviations as defined in Jairajpuri & Ahmad [44]. a, body length/maximum body width; b, body length/pharyngeal length; c, body length/tail length; c', tail length/body width at anus; V (distance from anterior end to vulva/body length) x 100; T (distance from cloacal aperture to anterior end of testis/body length) x 100; J (hyaline tail region length).

#### Measurements, morphology and distribution

Morphometric variability is described in Table 8 and morphological traits in Figs 9 and 10. *Longidorus silvestris* sp. nov. was only found in type locality from the rhizosphere of wild olive (Table 1, Fig 1).

#### Relationships

On the basis of body and odontostyle length, distance between guiding-ring from anterior body end, a, c and c´ ratios, amphidial fovea, or female tail shape, *L*. *silvestris* sp. nov. is very closely related to *L*. *wicuolea* sp. nov. from which it can be differentiated by a combination of these characters, but particularly in lip region shape (continuous *vs* separated from body contour by slight depression), and J1 tail shape (conoid-subdigitate *vs* conoid) (Figs 9 and 10). In addition, according to the polytomous key Chen *et al*. [64] and the supplement by Loof and Chen [65], and on the basis of sorting on matrix codes A (odontostyle length), B (lip region width), C (distance of guiding-ring from anterior body end), F (body length), and H (tail shape), *L*. *silvestris* sp. can be related with *L*. *belloi* Andrés & Arias, 1988 [74], *L*. *igoris* Krnjaić, Lamberti, Krnjaić, Agostinelli & Radicci, 2000 [75], and *L*. *moesicus* Lamberti, Choleva & Agostinelli, 1983 [76]. From *L*. *belloi* it differs mainly in having a slightly shorter odontostyle length (76.0–89.0 *vs* 74.8–101.7 μm), slightly higher c´ ratio (0.7–1.0 *vs* 0.5–1.1), frequency of males (extremely rare *vs* common), amphidial fovea shape (symmetrically *vs* asymmetrically bilobed) and J1 tail shape (conoid-subdigitate vs conoid) [73, 74]. On the other hand, *L*. *silvestris* sp. nov. differs mainly from *L*. *igoris* by lower a ratio (73.0–101.4 *vs* 103.0–131.7) and J1 tail shape (conoid-subdigitate *vs* cylindrical) [75]. Finally, the new species differs mainly from *L*. *moesicus* in having lower a ratio (73.0–101.4 *vs* 96.0–147.0) and a shorter odontostyle length (76.0–89.0 *vs* 97.0–124.0 μm) [76, 77]

#### Molecular divergence of the new species

*Longidorus silvestris* sp. nov. was closely related in D2–D3 (KT308859-KT308860) to *L*. *wicuolea* sp. nov. (KT308863-KT308866) with 98% similarity (Table 5). Intraspecific variation of D2–D3 detected between the two studied populations was low, 6 nucleotides and no indels. ITS1 also agree with the results obtained for D2–D3, this sequence (KT308884) was 90% similar to *L*. *wicuolea* sp. nov. (KT308887-KT308889). Finally, the partial 18S (KT308898) showed high homology with several sequences deposited in the GenBank, such as *L*. *magnus* (HM92921345, KT308902), *L*. *vinearum* (KT308903), *L*. *lusitanicus* (KT308901) and *L*. *wicuolea* sp. nov. (KT308900).

**Fig 11:**
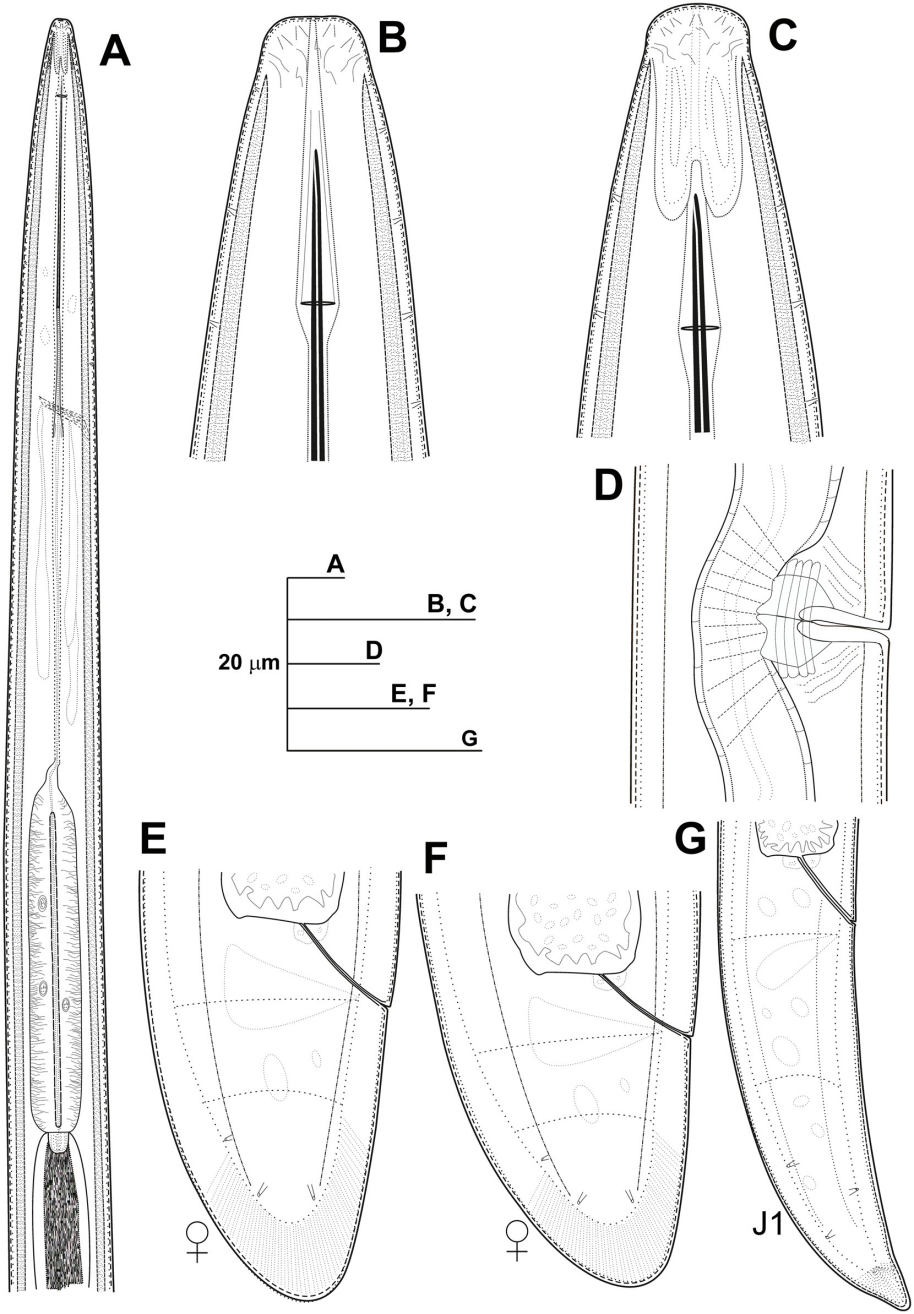
Line drawings of *Longidorus vallensis* sp. nov., female paratypes, male and juvenile stages. A) Pharyngeal region. B, C) Details of lip region. D) Vulval region. E, F) Female tails. G) First-stage juvenile tail (J1).

**Fig 12:**
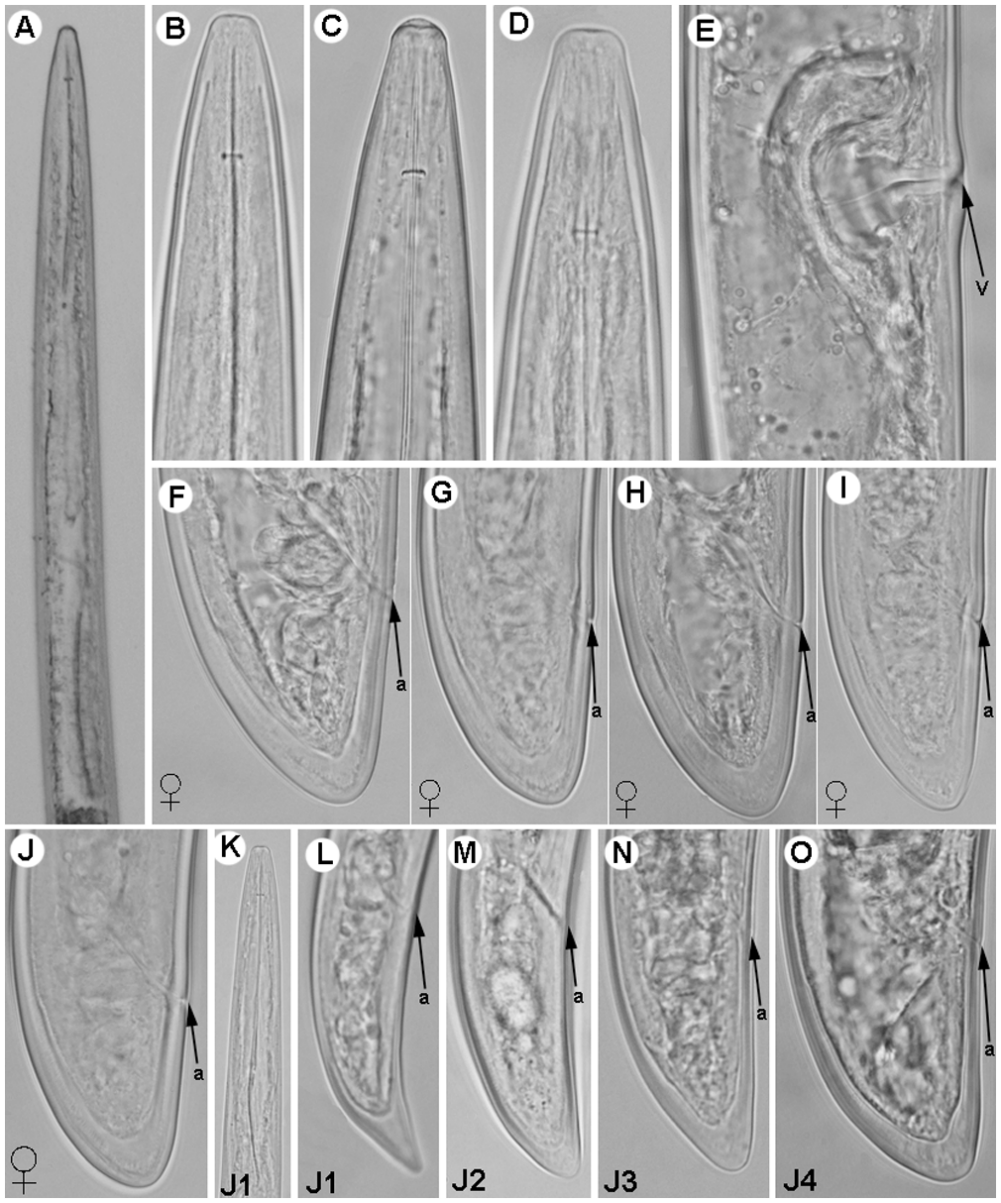
Light micrographs of *Longidorus vallensis* sp. nov., female paratypes, male and juvenile stages. A) Pharyngeal region. B-D) Female lip regions. E) Vulval region. F-J) Female tails. K) First-stage juvenile lip region showing replacement odontostyle inside odontophore. L-O) First-, second-, third-, and fourth-stage juvenile (J1–J4) tails, respectively. R) Male tail with detail of spicules. Abbreviations: a = anus; af = amphidial fovea; ca = cardias; gr = guiding-ring; n = nucleus; Rost = replacement odontostyle; sp = spicules. Scale bars = 20 μm.

***Longidorus vallensis* Archidona-Yuste, Navas-Cortés, Cantalapiedra-Navarrete, Palomares-Rius & Castillo, sp. nov.** urn:lsid:zoobank.org:act:9C1B1CB3-F8CE-422B-BAE1-984B1BFE2173 Figs 4, 11 and 12.

#### Holotype

Adult female, collected from the rhizosphere of wild olive (*Olea europaea* subsp. *silvestris* (Miller) Lehr) (36°37'57.3"N, 005°46'20.0"W), at San José del Valle, Cádiz province, Spain; collected by A. Archidona-Yuste, March 17, 2013; mounted in pure glycerine and deposited in the nematode collection at Institute for Sustainable Agriculture (IAS) of Spanish National Research Council (CSIC), Córdoba, Spain (collection number AR55-16).

#### Paratypes

Female, male and juvenile paratypes extracted from soil samples collected from the same locality as the holotype; mounted in pure glycerine and deposited in the following nematode collections: Institute for Sustainable Agriculture (IAS) of Spanish National Research Council (CSIC), Córdoba, Spain (collection numbers AR55-01-AR55-13); two females at Istituto per la Protezione Sostenibile delle Piante (IPSP), Consiglio Nazionale delle Ricerche (CNR), Bari, Italy (AR55-14); two females at Royal Belgian Institute of Natural Sciences, Brussels, Belgium (RIT8340); and two females at USDA Nematode Collection, Beltsville, MD, USA (T-6633p); collected by A. Archidona-Yuste, March 17, 2013.

#### Diagnosis

*Longidorus vallensis* sp. nov. is characterized by a long and thin body (6.2–8.7 mm), assuming an open C-shaped when heat relaxed; lip region anteriorly rounded separated from body contour by slight depression, 9.0–10.0 μm wide; guiding-ring located 25–30 μm from anterior end; odontostyle moderately long and narrow (71.5–85.0 μm); amphidial fovea pocket-shaped slightly symmetrically bilobed; vulva almost equatorial; female relatively tail short, convex-conoid to bluntly conoid, and bearing three pairs of caudal pores; c’ ratio (1.0–1.4); males not found; and specific D2–D3, ITS1 rRNA and partial 18S rRNA sequences (GenBank accession numbers KT308861-KT308862, KT308885-KT308886, and KT308899, respectively). According to the polytomous key Chen *et al*. [64] and the supplement by Loof and Chen [65], the new species has the following code (codes in parentheses are exceptions): A2(3)-B1-C2-D2-E2-F4(3)-G3-H2-I1.

#### Etymology

The species epithet refers to San José del Valle, the name of the type locality, Cádiz province, where the type specimens were collected.

#### Description of taxa. Female

Body long and thin, almost cylindrical, tapering in both extremities, especially in the anterior end. When heat relaxed, body usually assuming a spiral to an open C-shaped. Cuticle appearing smooth under low magnifications, 2.5 ± 0.8 (1.5–4.5) μm thick at mid body, but thicker (5.7 ± 1.4 (3.5–7.5) μm) and marked by very fine superficial transverse striate mainly in tail region, as shown by higher magnifications. Lip region anteriorly rounded, separated from body contour by slight depression. Amphidial fovea pocket-shaped slightly symmetrically bilobed. Labial papillae prominent. Stylet guiding-ring single, located 2.8 ± 0.1 (2.6–3.0) times lip region diam. from anterior end. Lateral chord 13.9 ± 1.9 (12.0–16.0) μm wide at mid-body or 20–30% of corresponding body diam. Odontostyle moderately long and narrow, straight or slightly arcuate, 1.8 ± 1.9 (1.5–2.0) times as long as odontophore, *ca* 3.0–3.5 μm wide towards its base; odontophore weakly developed, with rather weak basal swellings. Nerve ring encircling cylindrical part of pharynx, 2.2 ± 0.2 (1.9–2.7) times body width at neck base far from anterior end. Anterior slender part of pharynx usually coiled in its posterior region. Basal bulb relatively long and cylindrical, 118.5 ± 8.0 (106.5–135.0) μm long or *ca* one-third of neck length, 18.4 ± 2.0 (16.0–22.5) μm diam. Dorsal pharyngeal gland nucleus (DN) and ventro-sublateral pair of nuclei (SN) situated slightly posterior to normal arrangement of pharyngeal glands [64, 65], 34.1 ± 4.8 (27.8–40.7)%, 57.8 ± 5.0 (52.1–69.7)% of distance from anterior end of pharyngeal bulb, respectively. Dorsal gland nucleus (DN) slightly larger than nuclei of two SVN (2.5–3.5 *vs* 1.5–2.5 μm in diam.). Glandularium 102.3 ± 4.9 (95.0–113.0) μm long. Cardia conoid-rounded, 9.2 ± 2.6 (7.0–12.0) μm long. Reproductive system with both genital branches equally developed, very short compared with body length, ranging between 335–597 μm long, with reflexed ovaries variable in length. Vulva in form of a transverse slit, located about mid-body, vagina perpendicular to body axis, 23.0 ± 4.6 (16.0–30.0) μm long, or 30–50% of corresponding body width, surrounded by well-developed muscles. Genital branches equally developed, 5.6 ± 1.3 (4.3–8.2), 6.0 ± 1.3 (4.8–8.3)% of body length, respectively. Uteri short, without sperm cells in the female specimens examined. Anterior and posterior oviduct of similar size. Ovaries equally developed, 106–147 μm long, both of them with a single row of oocytes. Prerectum variable in length, 984.1 ± 133.2 (800–1194) μm long, and rectum 27.2 ± 2.6 (23.5–32.0) μm long, anus a small rounded slit. Tail relatively short, convex-conoid to bluntly conoid, with rounded terminus, bearing three pairs of caudal pores.

#### Male

Not found.

#### Juveniles

Morphometrics obtained from juvenile specimens, and of the relative lengths of body, tail, and functional and replacement odontostyle, confirmed the presence of four juvenile stages (Table 9, Figs 4 and 12; [66, 67]). J1s were characterised by a conoid tail, dorso-ventrally curved with rounded terminus, and slightly depression at hyaline region level, c’ ratio ≥ 2.3 (Table 9); an odontostyle length *ca* 53 μm, and shorter distance from anterior end to stylet guiding-ring than that in adult stages.

**Table 9:** Morphometrics of females, males and juvenile stages of *Longidorus vallensis* sp. nov. from the rhizosphere of cultivated and wild olives at several localities (Cádiz and Córdoba provinces) southern Spain^a^.

Host/locality, sample code	wild olive, San José del Valle (Cádiz province), AR55						cultivated olive Cabra (Córdoba province), M0012
Characters/ratios^b^	Holotype	Paratype Females	J1	J2	J3	J4	Female
**n**		17	5	5	5	5	1
**L (mm)**	7.0	7.6 ± 0.61 (6.2–8.7)	1.56 ± 0.57 (1.48–1.64)	2.66 ± 0.43 (2.23–3.34)	4.09 ± 0.50 (3.55–4.84)	5.86 ± 0.36 (5.41–6.39)	7.94
**a**	145.1	135.5 ± 6.2 (125.1–145.3)	74.5 ± 2.9 (71.0–77.6)	85.6 ± 5.0 (79.7–92.8)	108.0 ± 7.7 (100.4–118.9)	132.9 ± 8.5 (122.3–142.4)	149.8
**b**	16.1	18.6 ± 2.9 (12.6–23.6)	6.8 ± 1.4 (4.5–7.9)	10.1 ± 2.8 (6.1–13.9)	14.1 ± 3.5 (10.5–19.3)	17.6 ± 2.1 (14.5–19.8)	22.5
**c**	189.7	181.1 ± 18.6 (126.6–208.5)	38.4 ± 3.1 (34.9–43.4)	63.0 ± 4.0 (59.7–69.6)	87.4 ± 8.5 (81.3–99.8)	130.0 ± 11.7 (113.9–146.8)	198.5
**c´**	1.1	1.2 ± 0.1 (1.0–1.4)	2.6 ± 0.2 (2.3–2.7)	2.0 ± 0.1 (1.8–2.0)	1.7 ± 0.1 (1.6–1.8)	1.4 ± 0.1 (1.3–1.5)	1.1
**V**	53.5	51.4 ± 1.4 (49.5–53.5)	-	-	-	-	51.0
**Odontostyle**	80.5	79.1 ± 3.5 (71.5–85.0)	53.2 ± 1.6 (50.5–55.0)	55.2 ± 1.1 (54.5–57.0)	62.9 ± 1.4 (61.0–64.5)	73.3 ± 2.5 (69.5–76.0)	84.0
**Odontophore**	47.0	45.7 ± 3.2 (40.5–54.0)	26.8 ± 2.5 (24.5–29.5)	32.6 ± 6.9 (20.5–37.0)	45.4 ± 4.5 (40.5–51.0)	41.9 ± 6.8 (31.5–47.5)	56.0
**Replacement odontostyle**	-	-	59.3 ± 2.1 (57.0–62.0)	65.1 ± 1.9 (62.0–67.0)	73.5 ± 1.5 (71.5–75.5)	80.1 ± 3.6 (76.0–83.5)	-
**Lip region diam.**	10.0	9.7 ± 0.3 (9.0–10.0)	7.0 ± 0.7 (6.0–7.5)	7.9 ± 0.4 (7.5–8.5)	8.4 ± 0.2 (8.0–8.5)	9.1 ± 0.7 (8.5–10.0)	10.5
**Oral aperture-guiding ring**	28.0	27.3 ± 1.3 (25.0–30.0)	18.7 ± 1.5 (17.0–21.0)	22.0 ± 0.8 (21.0–23.0)	23.2 ± 1.3 (22.0–25.0)	26.5 ± 1.4 (25.0–28.5)	27.0
**Tail length**	42.5	42.0 ± 2.9 (37.5–49.0)	40.9 ± 2.8 (36.0–42.5)	42.1 ± 5.0 (35.0–48.0)	46.7 ± 1.9 (43.5–48.5)	45.2 ± 1.7 (43.5–47.5)	40
**J**	11.0	10.3 ± 1.1 (8.0–12.0)	11.6 ± 1.2 (9.5–12.5)	8.2 ± 0.6 (7.5–8.5)	8.5 ± 1.6 (6.5–10.5)	9.8 ± 1.6 (8.0–12.0)	10

^a^ Measurements are in μm (except for L) and in the form: mean ± standard deviation (range).

^b^ Abbreviations as defined in Jairajpuri & Ahmad [44]. a, body length/maximum body width; b, body length/pharyngeal length; c, body length/tail length; c', tail length/body width at anus; V (distance from anterior end to vulva/body length) x 100; J (hyaline tail region length).

#### Measurements, morphology and distribution

Morphometric variability is described in Table 9 and morphological traits in Figs 4, 11 and 12. In addition to the type locality, *L*. *vallensis* sp. nov. was found from one cultivated olive sample located in Córdoba province (Table 1, Fig 1).

#### Relationships

**Fig 13:**
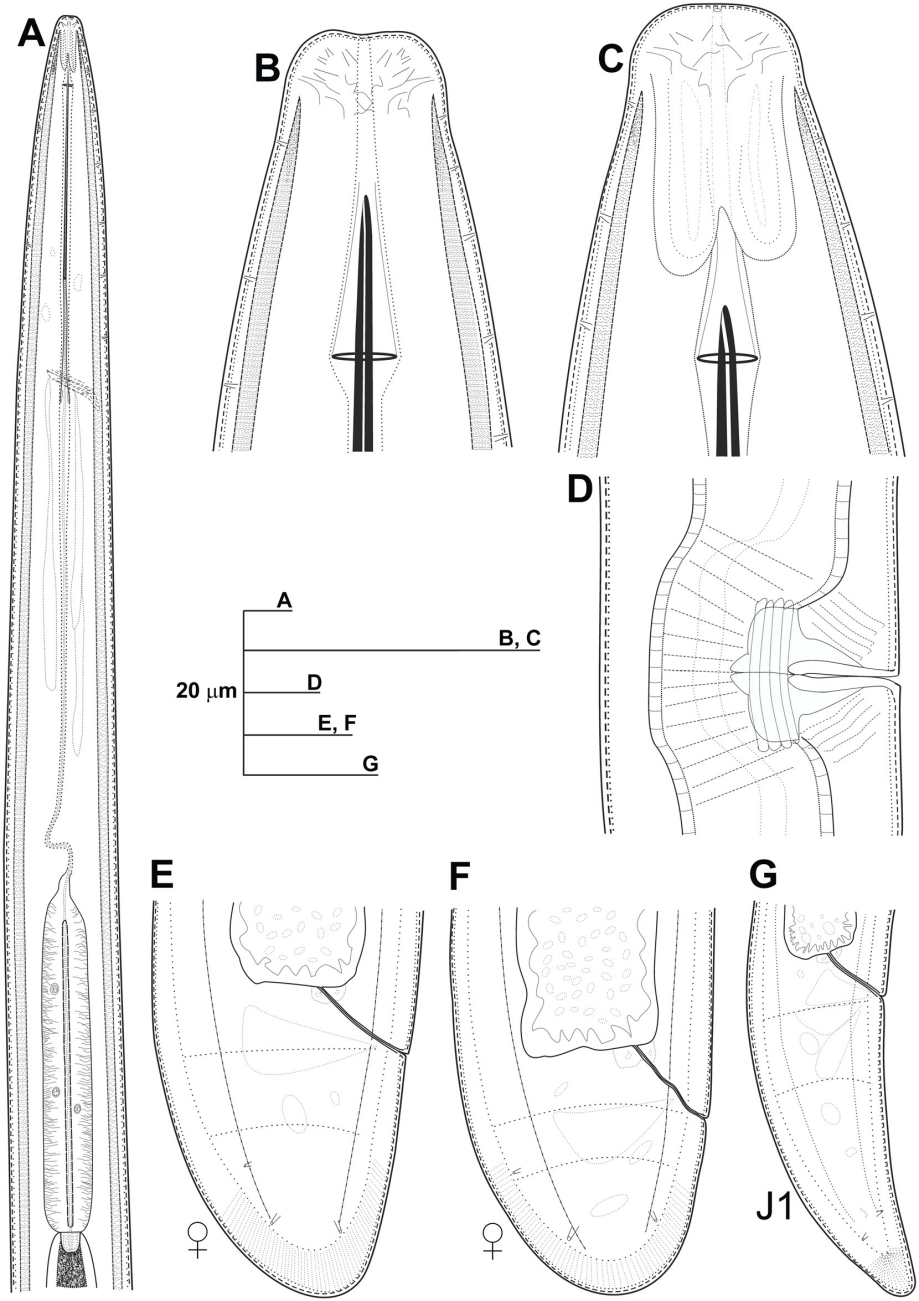
Line drawings of *Longidorus wicuolea* sp. nov., female paratypes, male and juvenile stages. A) Pharyngeal region. B, C) Details of lip region. D) Vulval region. E, F) Female tails. G) First-stage juvenile tail (J1).

**Fig 14:**
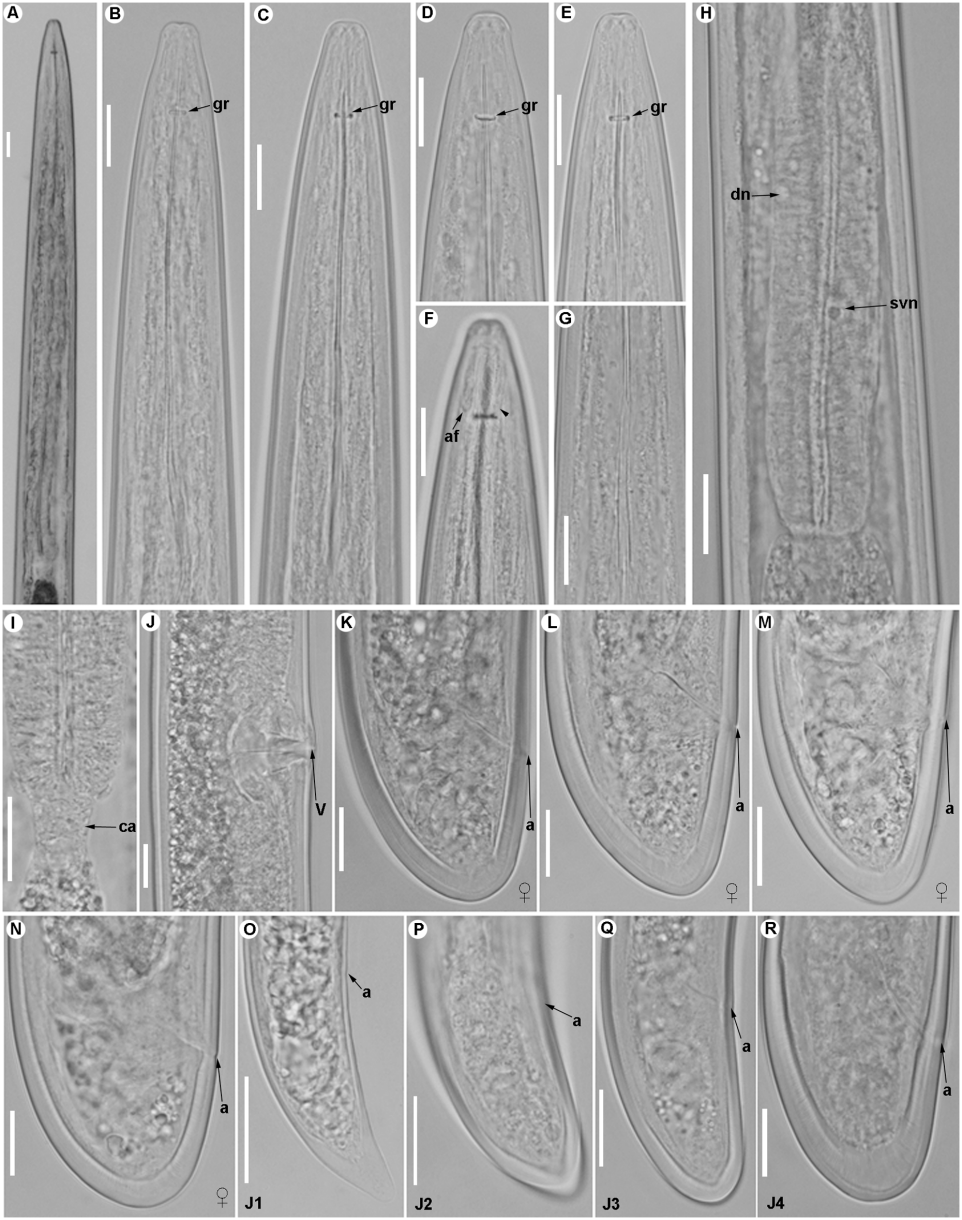
Light micrographs of *Longidorus wicuolea* sp. nov., female paratypes and juvenile stages. A) Pharyngeal region. B-C) Female neck regions. D-F) Female lip regions. G) Detail of odontophore. H) Detail of pharyngeal bulb. I) Detail of cardias (pharyngeal-intestinal junction). J) Vulval region. K-N) Female tails. O-R) First-, second-, third-, and fourth-stage juvenile (J1–J4) tails, respectively. Abbreviations: a = anus; af = amphidial fovea; ca = cardias; gr = guiding-ring; dn = dorsal nucleus; svn = subventral nucleus; V = vulva. Scale bars = 20 μm.

According to the polytomous key by Chen *et al*. [64] and the supplement by Loof and Chen [65], and on the basis of sorting on matrix codes A (odontostyle length), B (lip region width), C (distance of guiding-ring from anterior body end), D (lip region shape), F (body length), and H (tail shape), *L*. *vallensis* sp. nov. groups with *L*. *belloi*, *L*. *tabrizicus* Niknam *et al*., 2010 [78] and *L*. *wicuolea* sp. nov. From *L*. *belloi* it differs mainly in having higher a and c´ ratio (125.1–149.8 *vs* 73.0–132.0, 1.0–1.4 *vs* 0.5–1.1; respectively), and the absence vs presence of males [73, 74]. On the other hand, *L*. *vallensis* sp. nov. differs from *L*. *tabrizicus* mainly by a longer body and odontostyle length (6.2–8.7 *vs* 4.1–6.1 mm, 71.5–85.0 *vs* 61.5–70.0 μm; respectively), higher a and c ratio (125.1–149.8 *vs* 81.5–135.0, 126.6–208.5 *vs* 91.0–155.0; respectively), and the absence *vs* presence of males [78]. Finally, from *L*. *wicuolea* sp. nov. differs mainly in having higher a ratio (125.1–149.8 *vs* 79.3–115.6) and slightly higher c´ ratio (1.0–1.4 *vs* 0.8–1.2) (Table 10, Figs 13 and 14). In addition, *L*. *vallensis* sp. nov. is molecularly related to *L*. *rubi* from which it can be mainly differentiated by a longer body length (6.2–8.7 *vs* 4.0–6.0 mm), higher c ratio (126.6–208.5 *vs* 70.0–126.9) and lower c´ ratio (1.0–1.4 *vs* 1.7–2.1) [19, 68].

**Table 10:** Morphometrics of females and juvenile stages of *Longidorus wicuolea* sp. nov. from the rhizosphere of cultivated and wild olives at several localities (Sevilla and Huelva provinces) southern Spain^a^.

Host/locality, sample code	cultivated olive, Carmona (Sevilla province), JAO95						wild olive Bonares (Huelva, province), AR101
Characters/ratios^b^	Holotype	Paratype Females	J1	J2	J3	J4	Females
**n**		20	7	5	5	5	3
**L (mm)**	7.9	7.6 ± 0.68 (6.1–8.7)	2.06 ± 0.17 (1.86–2.25)	2.82 ± 0.34 (2.48–3.25)	3.81 ± 0.31 (3.27–4.02)	6.32 ± 1.00 (4.99–7.32)	7.5 ± 0.15 (7.4–7.7)
**a**	97.3	97.5 ± 9.1 (79.3–115.6)	62.8 ± 3.9 (58.0–69.0)	68.5 ± 5.3 (61.3–76.2)	82.2 ± 7.0 (71.2–88.8)	93.2 ± 4.9 (89.8–101.0)	102.6 ± 6.2 (95.6–107.0)
**b**	13.7	15.9 ± 2.5 (11.5–22.8)	9.1 ± 1.0 (7.8–10.4)	9.1 ± 2.2 (7.1–11.7)	11.5 ± 1.8 (9.8–14.4)	13.9 ± 2.8 (9.8–16.8)	16.3 ± 0.3 (15.9–16.6)
**c**	158.6	167.8 ± 14.9 (146.4–205.1)	47.5 ± 6.2 (39.7–54.6)	61.4 ± 5.8 (54.7–70.3)	79.2 ± 7.6 (71.6–91.4)	132.6 ± 35.9 (108.4–195.1)	177.3 ± 1.7 (175.9–179.1)
**c´**	1.0	0.9 ± 0.1 (0.8–1.2)	2.0 ± 0.2 (1.7–2.3)	1.6 ± 0.1 (1.5–1.8)	1.4 ± 0.1 (1.2–1.5)	1.0 ± 0.1 (0.8–1.1)	0.8 ± 0.1 (0.8–0.9)
**V**	51.0	50.6 ± 1.2 (48.0–52.5)	-	-	-	-	-
**Odontostyle**	86.0	86.4 ± 4.1 (77.0–94.0)	52.4 ± 1.7 (49.5–54.5)	59.6 ± 2.0 (57.0–62.0)	67.1 ± 2.4 (64.0–70.5)	79.0 ± 2.2 (75.5–81.0)	90.5 ± 6.1 (83.5–95.0)
**Odontophore**	50.0	47.8 ± 4.4 (39.5–59.0)	29.9 ± 4.4 (23.5–35.5)	38.6 ± 3.6 (34.5–44.0)	37.1 ± 3.2 (33.5–42.0)	45.0 ± 4.0 (40.5–49.0)	43.7 ± 3.8 (40.0–47.5)
**Replacement odontostyle**	-	-	61.0 ± 1.3 (58.5–62.0)	72.7 ± 1.3 (71.5–74.5)	79.0 ± 1.9 (77.0–81.5)	90.7 ± 4.8 (85.5–95.0)	-
**Lip region diam.**	10.5	10.8 ± 0.6 (9.5–12.0)	6.1 ± 0.5 (5.5–7.0)	7.3 ± 0.9 (6.5–8.5)	7.7 ± 0.7 (7.0–8.5)	9.1 ± 0.8 (8.5–10.0)	12.5 ± 0.9 (12.0–13.5)
**Oral aperture-guiding ring**	29.5	29.7 ± 1.6 (27.0–33.0)	20.7 ± 0.7 (19.5–21.5)	21.1 ± 1.7 (19.0–23.5)	23.9 ± 1.9 (22.0–27.0)	27.7 ± 2.0 (25.0–30.0)	26.8 ± 1.9 (25.5–29.0)
**Tail length**	50.0	45.6 ± 4.2 (37.5–56.0)	43.7 ± 2.8 (40.0–47.5)	46.1 ± 5.3 (41.5–55.0)	48.3 ± 4.5 (43.5–54.0)	48.8 ± 7.4 (37.5–55.5)	42.5 ± 0.9 (42.0–43.5)
**J**	14.5	9.3 ± 1.8 (6.0–14.5)	9.5 ± 0.9 (9.0–11.0)	6.6 ± 1.9 (5.0–8.5)	5.9 ± 1.8 (5.0–8.5)	7.1 ± 1.1 (6.0–8.5)	13.2 ± 2.5 (10.5–15.5)

^a^ Measurements are in μm (except for L) and in the form: mean ± standard deviation (range).

^b^ Abbreviations as defined in Jairajpuri & Ahmad [44]. a, body length/maximum body width; b, body length/pharyngeal length; c, body length/tail length; c', tail length/body width at anus; V (distance from anterior end to vulva/body length) x 100; J (hyaline tail region length).

#### Molecular divergence of the new species

The sequence divergence between *L*. *vallensis* sp. nov. (KT308861-KT308862) and other congeneric species were significant. The closet species in relation to D2–D3 region were *L*. *rubi* (JX445116, 96% similarity) and *L*. *indalus* sp. nov. (KT308852-KT308854, 91% similarity) (Table 5). Low intraspecific variation was detected in the two studied populations, differing in 3 nucleotides and 0 gaps. ITS1 (KT308885-KT308886) also showed some similarity with *L*. *rubi* (JX445098, 81%). No more similarity values above 80% were found in GenBank. Intraspecific variations for ITS1 sequences were 22 nucleotides, and 4 indels. The partial 18S of *L*. *vallensis* sp. nov. (KT308899) matched closely, 99%, with several *Longidorus* species, such as *L*. *rubi* (JX445125), *L*. *tabrizicus* (FJ009678), *L*. *closelongatus* (KJ802897) and *L*. *cretensis* (KJ802898).

***Longidorus wicuolea* Archidona-Yuste, Navas-Cortés, Cantalapiedra-Navarrete, Palomares-Rius & Castillo, sp. nov.** urn:lsid:zoobank.org:act:53950FE4-AA33-4301-AFE7-D143C0FC24AE Figs 4, 13 and 14

#### Holotype

Adult female, collected from the rhizosphere of cultivated olive (*Olea europaea* subsp. *europaea* L.) (37°28'37.4"N, 005°42'26.7"W), at Carmona, Sevilla province, Spain; collected by A. Archidona-Yuste, May 13, 2015; mounted in pure glycerine and deposited in the nematode collection at Institute for Sustainable Agriculture (IAS) of Spanish National Research Council (CSIC), Córdoba, Spain (collection number JAO95-17).

#### Paratypes

Female, male and juvenile paratypes extracted from soil samples collected from the same locality as the holotype; mounted in pure glycerine and deposited in the following nematode collections: Institute for Sustainable Agriculture (IAS) of Spanish National Research Council (CSIC), Córdoba, Spain (collection numbers JAO95-01-JAO95-16); one female at Istituto per la Protezione Sostenibile delle Piante (IPSP), Consiglio Nazionale delle Ricerche (CNR), Bari, Italy (JAO95-18); one female at Royal Belgian Institute of Natural Sciences, Brussels, Belgium (RIT841); and two females at USDA Nematode Collection, Beltsville, MD, USA (T-6634p); collected by A. Archidona-Yuste, March 17, 2013.

#### Diagnosis

*Longidorus wicuolea* sp. nov. is characterized by a long and robust body (6.1–8.7 mm), assuming an open C-shaped when heat relaxed; lip region anteriorly rounded, separated from body contour by a slight depression, 9.5–12.0 μm wide; guiding-ring located 27–33 μm from anterior end; odontostyle moderately long (77–94 μm); amphidial fovea pocket-shaped symmetrically bilobed; vulva almost equatorial; female relatively tail short, convex-conoid to bluntly conoid, and bearing two or three pairs of caudal pores; c’ ratio (0.8–1.2); males not detected; and specific D2–D3, ITS1 rRNA and partial 18S rRNA sequences (GenBank accession numbers KT308863-KT308866, KT308887-KT308889, and KT308900, respectively). According to the polytomous key Chen *et al*. [64] and the supplement by Loof and Chen [65], the new species has the following code (codes in parentheses are exceptions): A3(2)-B1(2)-C32-D2-E2-F4(3)-G2(1)-H12-I1.

#### Etymology

The species epithet refers to the first letters of its host plants name, wild (*wi*) and cultivated (*cu*) olive (*olea*), where the type specimens were collected.

#### Description of taxa. Female

Body long and robust, slightly tapering towards anterior end, usually assuming an open C-shaped when heat relaxed. Cuticle appears smooth, 3.8 ± 0.7 (2.5–5.0) μm thick, 6.5 ± 1.0 (5.5–8.0) μm thick at tail tip, and marked by very fine superficial transverse striae mainly in tail region. Lip region anteriorly rounded, separated from body contour by slight depression. Amphidial fovea pocket-shaped symmetrically bilobed, with lobes of about equal length, and extending about 3/4 part of anterior end-guiding ring distance. Labial papillae prominent. Guiding system with well-developed compensation sacs. Stylet guiding-ring single, located at 29.7 ± 1.6 (27.0–33.0) μm from anterior end. Odontostyle moderately long and narrow, 1.8 ± 0.2 (1.4–2.2) times as long as odontophore, straight or slightly arcuate; odontophore weakly developed, with rather weak basal swellings. Lateral chord *ca* 19% of corresponding body diam. Nerve ring encircling cylindrical part of pharynx, 1.7 ± 0.2 (1.4–2.0) times body width at neck base far from anterior end. Pharynx consisting of an anterior slender narrow part, extending to a terminal pharyngeal bulb, well demarcated anteriorly and cylindrical, 136.6 ± 9.8 (117.0–150.0) μm long, occupying *ca* 30% of total pharyngeal length, and 28.9 ± 3.0 (23.5–34.5) μm wide. Glandularium 11 ±0.6 ± 13.2 (92.0–136.55) μm long. Normal arrangement of pharyngeal glands [64, 65]: nuclei of the dorsal (DN) and subventral (SVN) pharyngeal gland located at 28.1 ± 2.8 (23.1–31.5), 56.8 ± 3.1 (50.9–61.6)% of distance from anterior end of pharyngeal bulb, respectively. Dorsal gland nucleus (DN) slightly larger than nuclei of two SVN (3.5–5.0 *vs* 3.0–4.5 μm in diam.). Cardia well developed, hemispherical to conoid, 14.1 ± 1.0 (12.5–15.5) μm long. Reproductive system with both genital branches equally developed, 7.4 ± 1.0 (5.7–9.8), 7.4 ± 0.8 (6.0–9.0)% of body length, respectively. Ovaries reflexed, very variable in length, *ca* 100–205 μm long. Vulva in form of a transverse slit, located about mid-body, vagina perpendicular to body axis, 30.9 ± 4.1 (20.0–36.0) μm long, or 30–50% of corresponding body width, surrounded by well-developed muscles. Uterus and oviduct of about equal length, without sperm cells in the female specimens examined. Ovaries equally developed ca 100–205 μm long, both of them with a single row of oocytes. Prerectum very variable in length, 11.1 ± 3.8 (6.4–16.3) times anal body diam., and rectum 1.6 ± 0.3 (1.3–2.0) times as long as anal body diam., anus a small rounded slit. Tail relatively short, convex-conoid to bluntly conoid, with rounded terminus, bearing two or three pairs of caudal pores.

#### Male

Not detected.

#### Juveniles

Morphologically similar to adults, but smaller. All four juvenile stages were found, being distinguishable by relative lengths of body and functional and replacement odontostyle (Table 10, Figs 4 and 14; [66, 67]). J1s were characterised by a conoid tail, dorso-ventrally curved with rounded terminus, and slightly depression at tip tail level, c’ ratio ≥ 1.7 (Table 10); an odontostyle length *ca* 52 μm, and shorter distance from anterior end to stylet guiding-ring than that in adult stages.

#### Measurements, morphology and distribution

Morphometric variability is described in Table 10 and morphological traits in Figs 4, 13 and 14. In addition to the type locality, *L*. *wicuolea* sp. nov. was extracted from one wild olive sample located in Huelva province, being distributed only in Western Andalusia (Table 1, Fig 1).

#### Relationships

On the basis of body and odontostyle length, distance between guiding-ring from anterior body end, a, c and c´ ratios, amphidial fovea, or female tail shape, *L*. *wicuolea* sp. nov. is very closely related to *L*. *silvestris* sp. nov. from which it can be differentiated by a combination of these characters, but particularly in lip region shape (separated from body contour by slight depression *vs* anteriorly rounded continuous), and J1 tail shape (conoid *vs* conoid-subdigitate) (Figs 9, 10, 13 and 14). In addition, according to the polytomous key Chen *et al*. [64] and the supplement by Loof and Chen [65], and on the basis of sorting on matrix codes A (odontostyle length), B (lip region width), C (distance of guiding-ring from anterior body end), D (shape of anterior region), F (body length), H (tail shape) and I (presence/absence of males), *L*. *wicuolea* sp. nov. can be related with *L*. *henanus* Xu & Cheng, 1992 [79] and *L*. *vallensis* sp. nov. From *L*. *henanus* it differs mainly in having a longer body and tail length (6.1–8.7 vs 3.8–7.0 mm, 37.5–56.0 vs 24.6–42.0 μm; respectively), a shorter odontostyle length (77.0–95.0 vs 90.5–104.0 μm) and a narrower lip region width (9.5–13.5 vs 13.2–18.0 μm) [79, 80, 81]. Finally, *L*. *wicuolea* sp. nov. differs basically from *L*. *vallensis* sp. nov. by lower a and c´ ratio (79.3–115.6 *vs* 125.1–149.8, 0.8–1.2 *vs* 1.0–1.4; respectively) (Table 9, Figs 11 and 12).

#### Molecular divergence of the new species

D2–D3 sequences from *L*. *wicuolea* sp. nov. (KT308863-KT308866) differed with the closest related species, *L*. *silvestris* sp. nov. (KT308859-KT308860) by 13 nucleotides (98% similarity) and from *L*. *magnus* (JX445112) and *L*. *vineacola* (JX445110) by 60 nucleotides (92% similarity) [Table 5]. Intraspecific variation of D2–D3 segments detected between the two studied populations of *L*. *wicuolea* sp. nov. consisted of 6 nucleotides (99% similarity), and no indels. Similarly, the ITS1 (KT308887-KT308889) also showed a low intraspecific variability between the two studied populations with only 4 nucleotides (99% similarity). The closet ITS1 to that of *L*. *wicuolea* sp. nov. was *L*. *silvestris* sp. nov. (KT308884) consisting in 73 nucleotides and 37 gaps (90% similarity). The partial 18S of *L*. *wicuolea* sp. nov. (KT308900) closely matched with several species of *Longidorus*, some of them were *L*. *magnus* (HM92921345, KT308902), *L*. *vinearum* (KT308903), *L*. *lusitanicus* (KT308901) and *L*. *silvestris* sp. nov. (KT308898).

### Morphology and morphometrics of known *Longidorus* species

Morphological and morphometrical data as well as molecular delineation (rDNA) of *L*. *alvegus*, *L*. *intermedius*, *L*. *magnus*, *L*. *oleae* and *L*. *vineacola* have been previously recorded within studies of dagger and needle nematodes infesting vineyards in southern Spain [19, 28]. The new records of these species from wild and cultivated olive in Granada and Sevilla provinces presented here extend the geographical distribution of these species (S1 and S2 Tables) in southern Spain [19]. Consequently, only D2–D3 sequences had been reported here for these samples. For other known species studied, representing the first molecular characterization and new records for olive or for Spain (*viz*. *L*. *lusitanicus* and *L*. *vinearum*), a brief description and a morphometric comparison with previous records and paratypes is provided below (S1–S3 Figs, S1 and S2 Tables).

#### *Longidorus lusitanicus* Macara 1985

The gonochoristic population of *Longidorus* from wild olive at Sanlúcar de Barrameda (Cádiz province) agrees fairly well with studied paratypes and original description of *L*. *lusitanicus*. This population was characterised by a lip region expanded or distinctly offset by constriction, rounded laterally and almost flattened frontally; amphidial fovea pouch-shaped, distinctly asymmetrically bilobed; female tail conoid-rounded; and the same proportion of male specimens found (S1 Table, S2 Fig). Morphometrics were coincident with those provided in the original description, except for only minor differences in oral aperture-guiding ring distance, which may be due to few specimens originally studied, or geographical intraspecific variability [45]. This is the first report for Spain and confirms a wider distribution in the Iberian Peninsula, apart from original description in Portugal. According to the polytomous key Chen *et al*. [64] and the supplement by Loof and Chen [65], this species has the following code: A3 B34 C23 D4 E3 F234 G2 H1 I2.

D2–D3 segments of *L*. *lusitanicus* (KT308869) was 95% similar to *L*. *vinearum* (KT308874-KT308877), *L*. *goodeyi* (AY601581), *L*. *magnus* (JX445112) and *L*. *onubensis* sp. nov. (KT308857-KT308858). The ITS1 of *L*. *lusitanicus* (KT308891) showed some homology with *L*. *onubensis* sp. nov. (81% similarity) and scarce homology with other ITS1 sequences from *Longidorus* species available in GenBank. The partial 18S region of *L*. *lusitanicus* (KT308901), was very similar to several sequences of *Longidorus* spp., including *L*. *vineacola* (JX445153, AY283169), *L*. *magnus* (HM921345) and *L*. *onubensis* sp. nov. (KT308897).

#### *Longidorus vinearum* Bravo & Roca, 1995

The four gonochoristic populations of *L*. *vinearum* from wild olive at Santa María de Trassierra (Córdoba province) agree fairly well with studied paratypes and original description of *L*. *vinearum*. The four studied populations were characterised by a robust and long body, lip region anteriorly rounded and separated from body contour by a very slight depression; amphidial fovea pouch-shaped, distinctly asymmetrically bilobed; female tail short, bluntly rounded to hemispherical with rounded terminus; and the common presence of male specimens (S2 Table, S3 Fig). Morphometrics of female, male and J1 specimens were coincident with those provided in the original description and rather similar to data reported subsequently for other populations of Portugal, except for minor differences in a ratio and length of spicules, which may be due to few specimens originally studied or geographical intraspecific variability [46, 73]. This is the first report for Spain and confirms a wider distribution in the Iberian Peninsula, apart from original description and other populations in Portugal. According to the polytomous key Chen *et al*. [64] and the supplement by Loof and Chen [65], this species has the following code: A45 B345 C34 D2 E3 F345 G12 H1 I2.

The closet species regarding D2–D3 segments of *L*. *vinearum* (KT308874-KT308877) were *L*. *magnus* (HM921361, JX445112, 96% similarity) and *L*. *goodeyi* (AY601581, 94%). ITS1 (KT308892-KT308893) region also showed some similarity with *L*. *magnus* (HM921340, 90% similarity), but no more similarity values above 80% were found in GenBank. The partial 18S of *L*. *vinearum* (KT308903) matched closely (99%) with several *Longidorus* spp., such as *L*. *vineacola* (JX445153, AY283169) and *L*. *magnus* (HM921345).

### Phylogenetic relationships of the *Longidorus* spp.

The amplification of D2–D3 expansion segments of 28S rRNA, ITS1 rRNA, and partial 18S rRNA yielded a single fragment of approximately 800 bp, 1000 bp, and 1500 bp, respectively, based on gel electrophoresis. Sequences from other species of *Longidorus* spp. obtained from National Center for Biotechnology Information (http://www.ncbi.nlm.nih.gov/) were used for further phylogenetic studies. Sequences for *L*. *indalus* sp. nov., *L*. *lusitanicus*, *L*. *macrodorus* sp. nov., *L*. *onubensis* sp. nov., *L*. *silvestris* sp. nov., *L*. *vallensis* sp. nov., *L*. *vinearum*, and *L*. *wicuolea* sp. nov., were obtained for these species in this study. On the other hand, sequences for *L*. *alvegus* (KT308867), *L*. *intermedius* (KT308868, KT308890), *L*. *magnus* (KT308870), *L*. *oleae* (KT308871) and *L*. *vineacola* (KT308872, KT308873) matched well with former sequences deposited in GenBank, extending the molecular diversity of these species to the newly studied areas.

**Fig 15:**
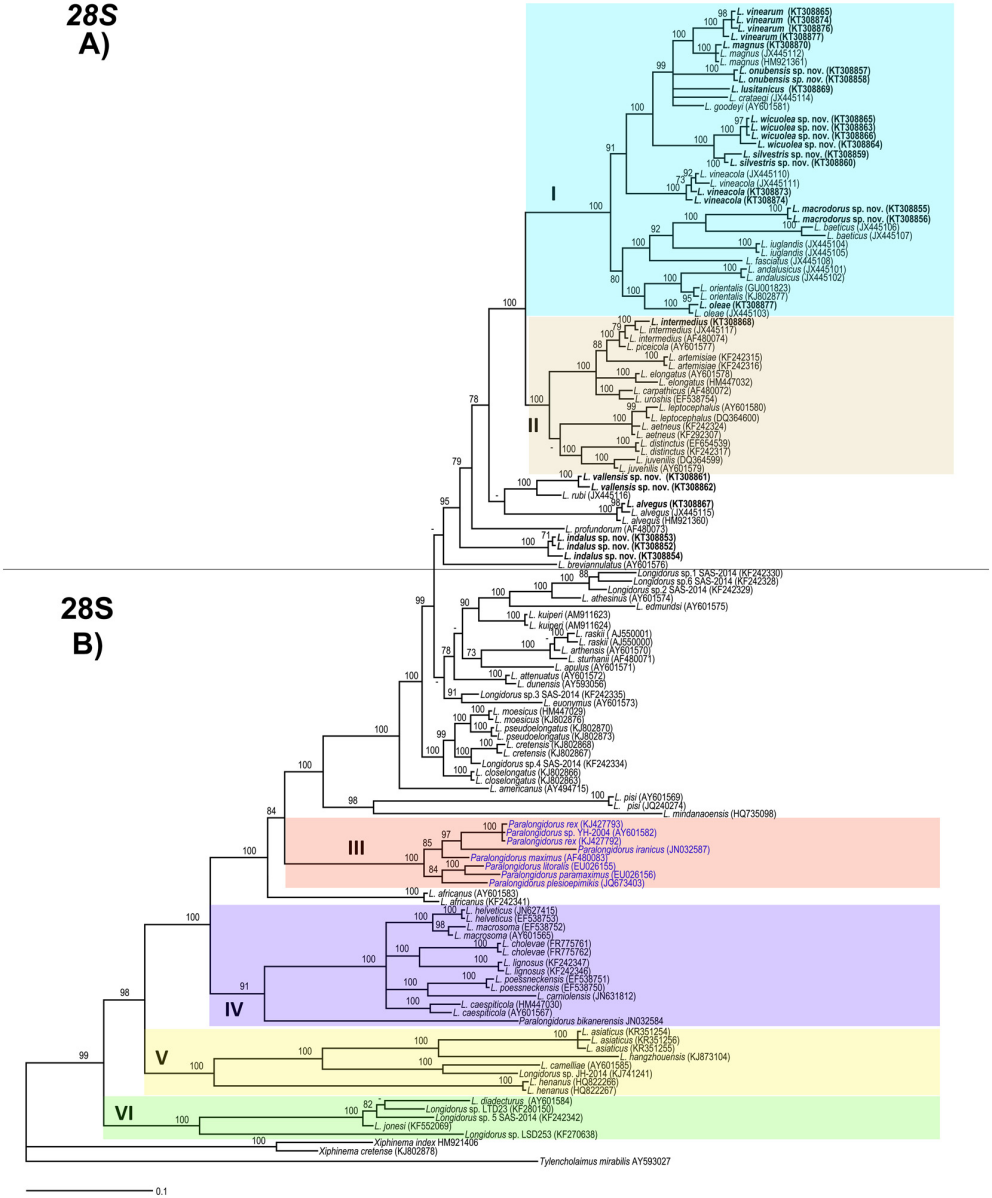
The 50% majority rule consensus tree from Bayesian inference analysis generated from the D2–D3 of 28S rRNA gene dataset of *Longidorus* spp. with the SYM+I+G model. Posterior probabilities more than 70% are given for appropriate clades. Newly obtained sequences are in bold letters. Scale bar = expected changes per site. **A)**. Clades I & II. **B)**. Clades III-VI.

**Fig 16:**
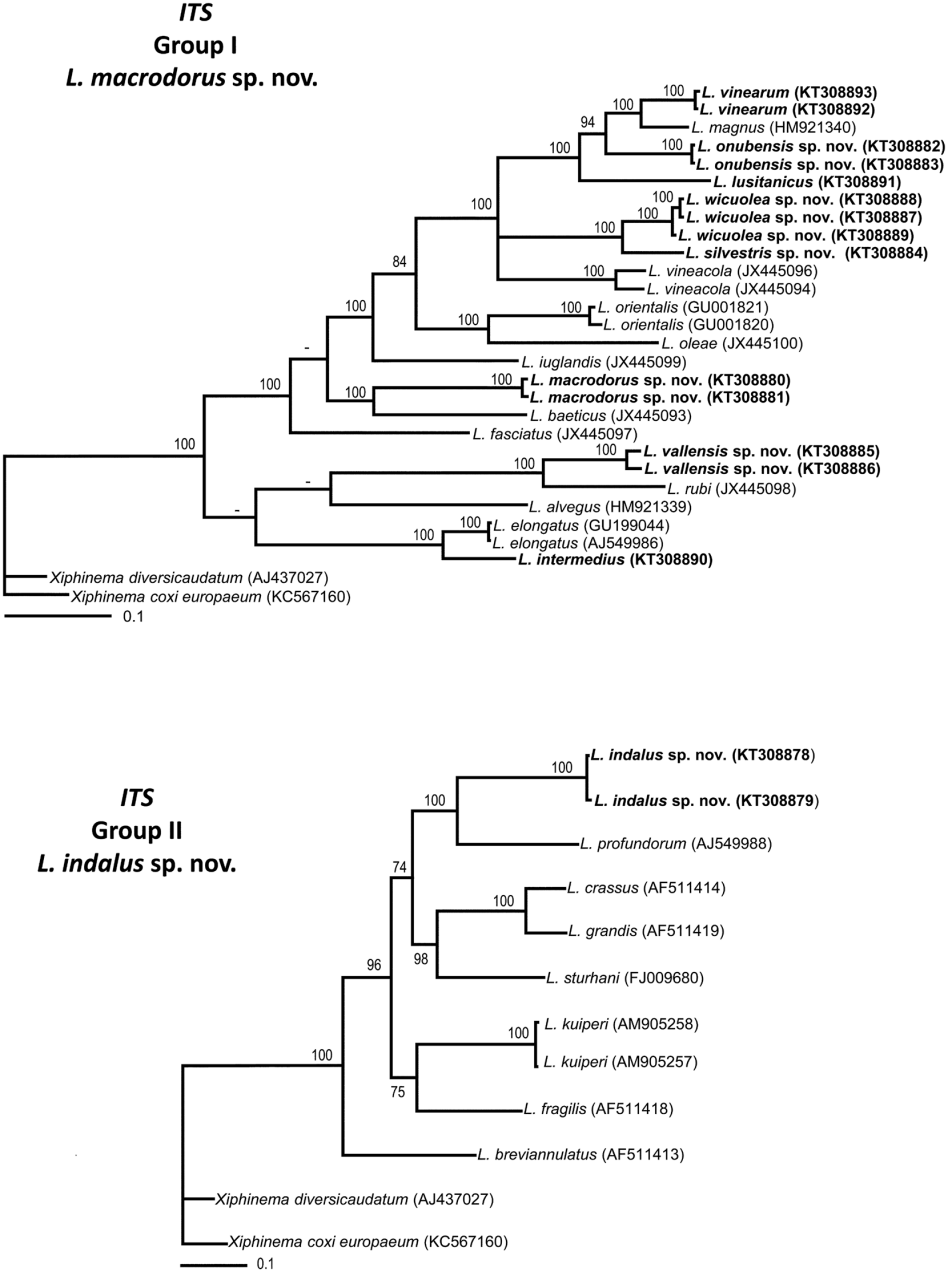
The 50% majority rule consensus trees from Bayesian inference analysis generated from the ITS rRNA gene dataset of *Longidorus macrodorus* sp. nov. group and *L*. *indalus* sp. nov. group with the TVM+I+G and TIM3+I+G models, respectively. Posterior probabilities more than 70% are given for appropriate clades. Newly obtained sequences are in bold letters. Scale bar = expected changes per site.

**Fig 17:**
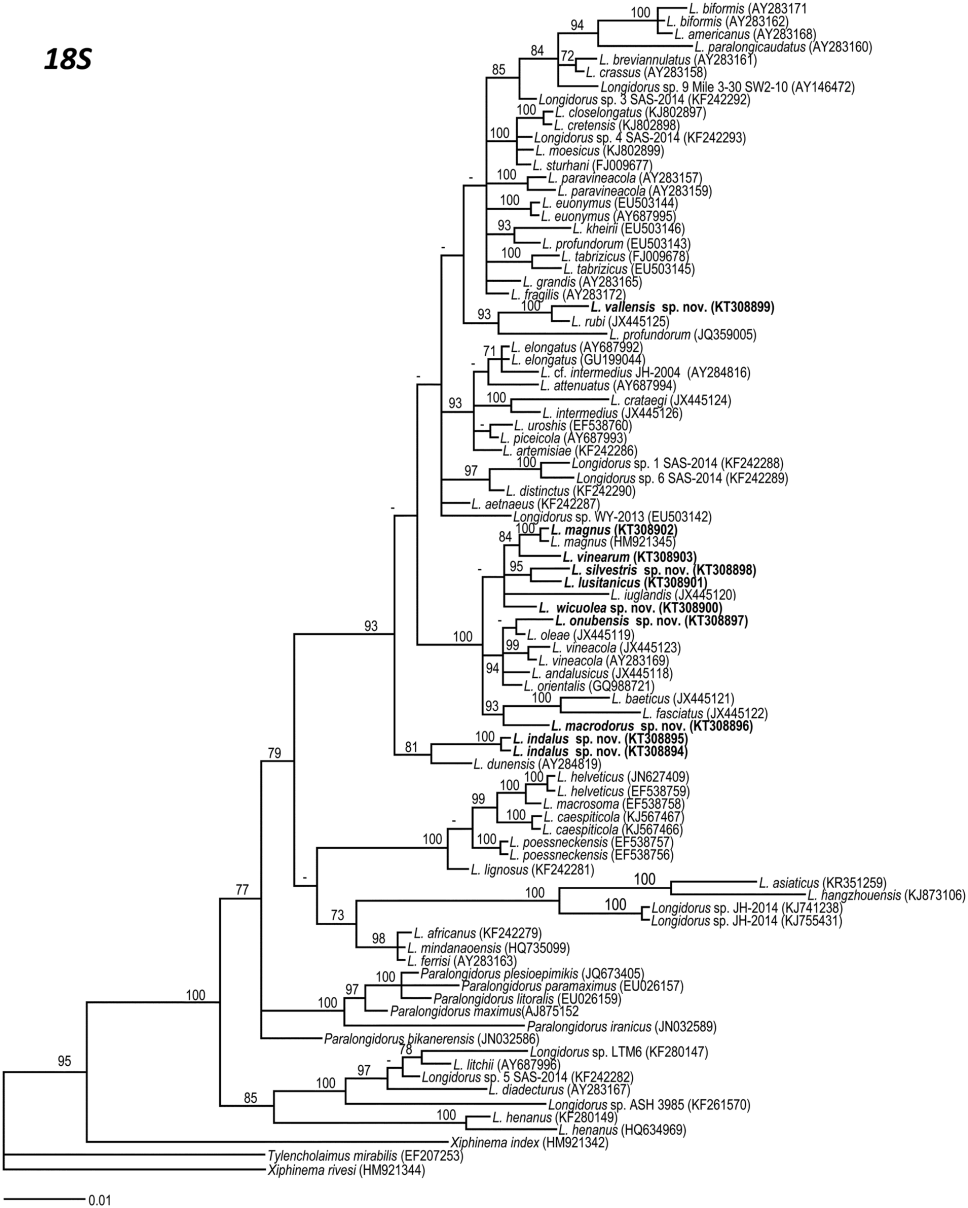
The 50% majority rule consensus trees from Bayesian inference analysis generated from the partial 18S rRNA gene dataset of *Longidorus* spp. with the TVMef+I+G model. Posterior probabilities more than 70% are given for appropriate clades. Newly obtained sequences are in bold letters. Scale bar = expected changes per site.

Phylogenetic relationships among *Longidorus* species inferred from analyses of D2–D3 expansion segments of 28S, ITS1, and the partial 18S rDNA gene sequences using BI are given in Figs 15, 16 and 17, respectively. To facilitate discussion, clades that were well supported or are taxonomically well founded are labelled in roman numerals from I through VII (Fig 15). Poorly supported lineages are not explicitly labelled. The 50% majority rule consensus 28S rRNA gene BI tree of *Longidorus* and *Paralongidorus* spp. based in a multiple edited alignment including 133 sequences and 748 total characters consisted of six moderate to highly supported major clades in the genus (Fig 15). Clade I is well-supported (PP = 100%) comprising 16 species including nine reported in olives: *L*. *vinearum* (KT308874-KT308877), *L*. *onubensis* sp. nov. (KT308857-KT308858), *L*. *silvestris* sp. nov. (KT308857-KT308860), *L*. *lusitanicus* (KT308869), *L*. *wicuolea* sp. nov. (KT308863-KT308866) and *L*. *macrodorus* sp. nov. (KT308855-KT308856), *L*. *magnus* (JX445112, HM921361, KT308870), *L*. *oleae* (JX445103, KT308871), *L*. *vineacola* (JX445110-JX445111, KT308873-KT308874) and other *Longidorus* spp. from the Mediterranean Basin such as *L*. *andalusicus* (JX445101-JX445102), *L*. *fasciatus* (JX445108), *L*. *iuglandis* (JX445104- JX445105), *L*. *crataegi* (JX445114), *L*. *baeticus* (JX445106- JX445107), *L*. *orientalis* (GU001823, KJ802877), and *L*. *goodeyi* (AY601581) from UK. All these species shared a hemispherical, convex-conoid and short tail. Clade II is well-supported (PP = 100%) comprising ten species and including *L*. *intermedius* (AY601577, JX445117, KT308868). *Longidorus vallensis* sp. nov. (KT308861-KT308862), was phylogenetically related to *L*. *rubi* (JX445116) forming a well-supported clade (PP = 100%), and with *L*. *alvegus* (JX445115, HM921360, KT308867) which formed a sister-clade, however the BI values for this sister-clade is low. Finally, *L*. *indalus* sp. nov. (KT308852-KT308854) did not form supported clades with any of *Longidorus* species. Clade III is also well-supported (PP = 100%) and comprised all *Paralongidorus* species, except *P*. *bikanerensis* (JN032584), which clustered in the moderately supported (PP = 81%) clade IV with other species from different geographical origin. Clade V and VI are well-supported (PP = 100%) and comprised five species of Asiatic origin, and a basal well-supported (PP = 100%) clade VI with four species from different geographical origin (Fig 15).

Difficulties were experienced with alignment of the ITS1 sequences due to scarce homology, thus, related sequences were divided into two different groups in our study (Fig 16). The first group included 752 characters and 29 sequences comprising several *Longidorus* species also from the Mediterranean Basin and with hemispherical, convex-conoid and short tail, *L*. *lusitanicus* (KT308891), *L*. *macrodorus* sp. nov. (KT308880-KT308881), *L*. *onubensis* sp. nov. (KT308882-KT308883), *L*. *silvestris* sp. nov. (KT308857-KT308860), *L*. *wicuolea* sp. nov. (KT308884), *L*. *vinearum* (KT308892-KT308893), *L*. *vallensis* sp. nov. (KT308885-KT308886), and *L*. *intermedius* (KT308890) with a short body length (Fig 16). These results agree with those obtained for D2–D3 segments. This phylogenetic tree resolved two major well supported (PP = 100%) clades, *L*. *vinearum*, *L*. *lusitanicus*, *L*. *onubensis* sp. nov., *L*. *silvestris* sp. nov. and *L*. *wicuolea* sp. nov. were placed within the first major clade. *Longidorus vinearum*, *L*. *lusitanicus* and *L*. *onubensis* sp. nov. formed a high supported subclade (PP = 100%) with *L*. *magnus* (HM921340). *Longidorus wicuolea* sp. nov. was placed within another well supported subclade (PP = 100%) with *L*. *silvestris* sp. nov. and finally *L*. *macrodorus* sp. nov. was phylogenetically related to *L*. *baeticus* (JX445093). Second major clade was low-supported and was formed by five *Longidorus* species, *L*. *vallensis* sp. nov. was placed with *L*. *rubi* (JX445098) in a high-supported subclade (PP = 100%) and it was related to *L*. *alvegus* (HM921339) which formed another low-supported subclade. Finally, *L*. *intermedius* and *L*. *elongatus* (GU199044) formed a high-supported subclade (PP = 100%), occupying a basal position in the tree (Fig 16).

The second group of the ITS1 sequences included 1126 characters and 12 sequences comprising ten *Longidorus* species characterized by a medium to short body length, including *L*. *indalus* sp. nov. (KT308878-KT308879), *L*. *profundorum* (AJ549988), *L*. *sturhani* (FJ009680), *L*. *crassus* (AF511414), *L*. *kuiperi* (AM905257-AM905258), *L*. *fragilis* (AF511418) and *L*. *breviannulatus* (AF511413). *Longidorus indalus* sp. nov. clustered with *L*. *profundorum* in a high supported clade (PP = 100%) (Fig 16).

The 50% majority rule BI tree of a multiple alignment including 90 18S sequences and 1687 bp and as well as in the D2–D3 and ITS1 tree, *L*. *lusitanicus* (KT308901), *L*. *macrodorus* sp. nov. (KT308896), *L*. *onubensis* sp. nov. (KT308897), *L*. *silvestris* sp. nov. (KT308898), *L*. *vinearum* (KT308903) and *L*. *wicuolea* sp. nov. (KT308900) clustered within the same well-supported (PP = 100%) clade with *Longidorus* species from Mediterranean Basin and sharing a convex-conoid female tail shape such as *L*. *andalusicus* (JX445118), *L*. *oleae* (JX445119), *L*. *vineacola* (JX445123, AY283169), *L*. *magnus* (HM921345-KT308902), *L*. *baeticus* (JX445121), *L*. *fasciatus* (JX445122) and *L*. *iuglandis* (JX445120). Phylogenetic inferences based on 18S also suggest that *L*. *vallensis* sp. nov. and *L*. *rubi* are close-related species (PP = 100%). Finally, *L*. *indalus* sp. nov. (KT308894-KT308895) clustered in this case with *L*. *dunensis* (AY284819) with a low support (PP = 81%).

## Discussion

The primary objective of this study was to unravel the biodiversity, distribution and molecular phylogeny of needle nematodes of the genus *Longidorus* associated with wild and cultivated olives in southern Spain. This was conducted in an extensive and systematic nematological survey that included 159 locations and 449 sampling sites. We found 40 Spanish populations of *Longidorus* spp. infesting olive soils. Our results demonstrate that the use of morphological studies together with rDNA molecular markers may decipher the specific biodiversity in this complex group of plant-parasitic nematodes. We described here six new *Longidorus* species, based on integrative taxonomy and the phylogenetic relationships of the genus *Longidorus* based on nuclear rDNA markers.

The comparative morphological and morphometrical study of the 40 Spanish populations of *Longidorus* spp. confirmed that diagnosis and identification of these species based solely on diagnostic morphometric features is quite complex since there is almost a continuous range of character measurements within populations as well as among species [8, 19]. The present results (including new and known species) enlarge the biodiversity of *Longidorus* in the Iberian Peninsula and agree with previous data obtained for the phylogeny and biogeography of the genus *Longidorus* in the Euro-Mediterranean region [19, 28, 82, 83], in which a dispersalist model was one of the primary explanations for the large groups of *Longidorus* species found in this region.

Considering the species richness of PPN associated with olive in different studies, the genus *Longidorus* is one of the most biodiverse with nine species (*viz*. *L*. *africanus*, *L*. *belloi*, *L*. *closelongatus*, *L*. *cretensis*, *L*. *elongatus*, *L*. *macrosoma*, *L*. *oleae*, *L*. *pseudoelongatus*, *L*. *siddiqii*, and *L*. *vinearum*) reported in several countries of the Mediterranean Basin such as Egypt, Greece, Jordan, Portugal and Spain [19, 35, 36, 73, 77, 84, 85, 86, 87, 88, 89]. Although all *Longidorus* spp. are obligate soil plant ecto-parasites of a wide range of wild and cultivated plants causing enlarged swellings of root tips, it is unlikely that these species could be detected in other wild and cultivated plants in the next future. The present results double the previous biodiversity of *Longidorus* species detected in olive worldwide, including six new species and two new records for wild and cultivated olives (*L*. *alvegus* and *L*. *vineacola*), as well as two additional new records for wild olives (*L*. *intermedius* and *L*. *lusitanicus*). The most recent major geological event having important effects for nematode biodiversity and distribution in Europe was the Quaternary glaciation which happened *ca*. 40,000 years ago. In Europe has been hypothesized that reduced species numbers in northern Europe is attributed to Quaternary glaciations, being the highly diverse nematofauna of the Mediterranean basin related to Miocene plate tectonics in that area [90]. Our study showed a great diversity in Southern Spain. However, because of no sampling North-South has been developed; more intensive studies are needed in northern areas in order to corroborate this hypothesis. The distribution of the 40 *Longidorus* populations collected in Andalusia showed that some of them revealed a certain geographic associations to western areas (*viz*. *L*. *alvegus*, *L*. *intermedius*, *L*. *lusitanicus*, *L*. *onubensis* sp. nov., *L*. *vineacola*, *L*. *vinearum*, *L*. *wicuolea* sp. nov.) and eastern regions (*viz*. *L*. *indalus* sp. nov.), while only *L*. *magnus* was detected in both areas (Fig 1). The present findings showed certain coincidences with the quantitative analysis of *Longidorus* spp. distribution carried out by Navas et al. [82], who recognized two main groups of species, the European-Atlantic and the Mediterranean. The widespread distribution of *L*. *magnus* may suggest a high ecological flexibility e.g. adaptability to a range of soil types, and reproduction sustained over a broad temperature range [91]. While other species seems to be better adapted to drier areas as it is the case for *L*. *indalus* sp. nov. in Eastern regions with markedly lower precipitation. Species showing a restricted distribution may be the result of isolation of populations in diverse biotopes which would result in reproductive isolation and hence the establishment of new species [91]. Also, although agricultural activities may result in the widespread dissemination of *Longidorus* species [91], the geographical distribution of *Longidorus* species in wild and cultivated olives in southern Spain suggest an established pattern related to ecological factors, on a geological timescale. These nematodes could have a lower dissemination by human activities than other plant-parasitic nematodes (i.e. cyst- or root-lesion nematodes) because of their sensitivity to fast desiccation, large body size, and the absence of survival-resistance forms. Unfortunately, little is known about the ecological requirements of *Longidorus* nematodes and elucidation of speciation and species biodiversity has currently to be approached on the groupings of morphometric characters [91]. Consequently, further research is needed in order to determine the influence of physico-chemical soil factors on the incidence and distribution of these nematodes in southern Spain and other wider areas.

Sequences of nuclear ribosomal RNA genes, particularly D2–D3 and ITS1, have proven to be a powerful tool for providing accurate species identification of Longidoridae [22, 25, 92]. However D2–D3 expansion region was more useful for establishing phylogenetic relationships among *Longidorus* species than ITS1 or 18S. The great diversity detected in the ITS1 suggests that a variety of poorly understood factors are involved in the fast evolution of this region in nematodes. Thus, ITS1 appears better suited for differentiation of species than for phylogenetic relationships within *Longidorus*. Our findings also confirm that partial 18S sequence does not have enough resolution to distinguish species, because different species showed a low nucleotide differences amongst them. Phylogenetic analyses based on D2–D3, ITS1, and partial 18S using BI resulted in a congruent position for the newly sequenced species of *Longidorus* spp. from Spain, which grouped in a separate clade, except for *L*. *vallensis* sp. nov. (KT308861-KT308862) and *L*. *indalus* sp. nov. (KT308852-KT308854) in the D2–D3, partial 18S, and ITS1 trees, which grouped separately (Figs 15, 16 and 17). *Longidorus vallensis* sp. nov. clustered in all ribosomal markers with *L*. *rubi*. However these species showed several morphological differences that made it difficult to establish a correspondence between morphological characters and the phylogenetic trees inferred from the molecular data. The majority of the species showed congruence in the phylogenetic relationships within these ribosomal markers using the DNA from the same individual. However *L*. *indalus* sp. nov. phylogenetic position was not congruent amongst the different ribosomal markers used here. This could be a result of different mutation rates within the different ribosomal markers, or difficulties in sequence alignment in ITS1 sequences. The phylogenetic relationships inferred in this study based on the D2–D3 and ITS1 sequences mostly agree with the lineages obtained by other authors [19, 25, 28, 93, 94]. Most of the newly and known described species in this research (*viz*. *L*. *lusitanicus*, *L*. *macrodorus* sp. nov., *L*. *magnus*, *L*. *oleae*, *L*. *onubensis*, *L*. *silvestris* sp. nov., *L*. *vineacola*, *L*. *vinearum*, *L*. *wicuolea* sp. nov.) grouped genetically in the same clade. These species shared a long body and odontostyle and can be considered as the most evolved species in the genus [14]. These traits could be related to the feeding habits of these nematodes, since longer stylets are better adapted to penetrate major woody plants roots persisting during the hot-dry summer conditions prevalent in Southern Spain and with long body sizes to move quickly deeper in the soil to avoid dry conditions in summer.

To confirm the correlation of the results obtained by conventional morphological approaches and new molecular methods is important for the proper understanding of the evolution of the genus *Longidorus*. The close relationship of the morpho-species groups detected in this and previous studies in Spain was also supported by molecular data (most of the species described were in the same clade), an observation that points to the Iberian Peninsula as a possible center of recent speciation [19], as it was suggested for other genera such as *Xiphinema* [5, 19, 22, 28], *Trichodorus* [95] or *Rotylenchus* species [11].

## Conclusions

In summary, the present study establishes the importance of using integrative taxonomic identification highlighting the difficulty of a correct identification at species level within the genus *Longidorus*. This study also provides molecular markers for precise and unequivocal diagnosis of some species of *Longidorus* in order to differentiate virus vector or quarantine species. This and previous studies demonstrate that the genus *Longidorus* is clearly a complex group and much work remains to be done to elucidate species boundaries in this economically important group of PPN. Furthermore, similar intensive and extensive integrative studies on *Longidorus* species in several wider areas may help to elucidate if *Longidorus* species have originated in Southeast Africa and India, when these two areas were still united, and a later spread to Laurasia was followed by a main speciation of *Longidorus* in the Holarctic region, especially Europe, as hypothesised by Coomans [14]. This hypothesis is reinforced with the basal position of Asian species in D2–D3 region and partial 18S phylogenetic trees.

## Supporting Information

S1 Fig Light micrographs of *Longidorus carpetanensis* Arias *et al*., 1986 (A-F) and *Longidorus unedoi* Arias *et al*., 1986 (G-L) paratypes from the Nematode Collection of the Spanish National Museum of Natural Sciences-CSIC, Madrid, Spain. A-B, G-H) Female anterior regions. C-D, I-J) Female tails. E-F, K-L) Male tails. Abbreviations: a = anus; gr = guiding-ring; spl = ventromedian supplements. Scale bars A-L = 20 μm.
(TIF)

S2 Fig Light micrographs of *Longidorus lusitanicus* Macara, 1985 from wild olive at Sanlúcar de Barrameda (Cádiz province) (A-F), and paratypes from the Nematode Collection at the Istituto per la Protezione Sostenibile delle Piante (IPSP), Consiglio Nazionale delle Ricerche (CNR), Bari, Italy (G-L). A-C, G-I) Female lip regions. J) Vulval region. D-E, K, L) Female tails. F) Male tail. Abbreviations: a = anus; af = amphidial fovea. (Scale bars = 20 μm).
(TIF)

S3 Fig Light micrographs of *Longidorus vinearum* Bravo & Roca, 1995 from wild olive at Santa Mª de Trassierra (Córdoba province) (A-G), and paratypes from the nematode collection at the Istituto per la Protezione Sostenibile delle Piante (IPSP), Consiglio Nazionale delle Ricerche (CNR), Bari, Italy (H-L). A-B, H-J) Female lip regions. C) Vulval region. D, K, L) Female tails. E-G) Male tails and detail of spicules. Abbreviations: a = anus; af = amphidial fovea. (Scale bars = 20 μm).
(TIF)

S1 Table Morphometrics of *Longidorus lusitanicus* Macara, 1985 and *Longidorus oleae* Gutiérrez-Gutiérrez *et al*., 2013 studied from southern Spain. (DOC)

S2 Table Morphometrics of *Longidorus vinearum* Bravo & Roca, 1995 populations studied from southern Spain. (DOC)
