# Supplementary figures and images for: Unravelling the Biodiversity and Molecular Phylogeny of Needle Nematodes of the Genus Longidorus (Nematoda: Longidoridae) in Olive and a Description of Six New Species
Source: PLoS One. 2016 Jan 25;11(1):e0147689. doi: 10.1371/journal.pone.0147689 (PMC4726821; doi:10.1371/journal.pone.0147689)

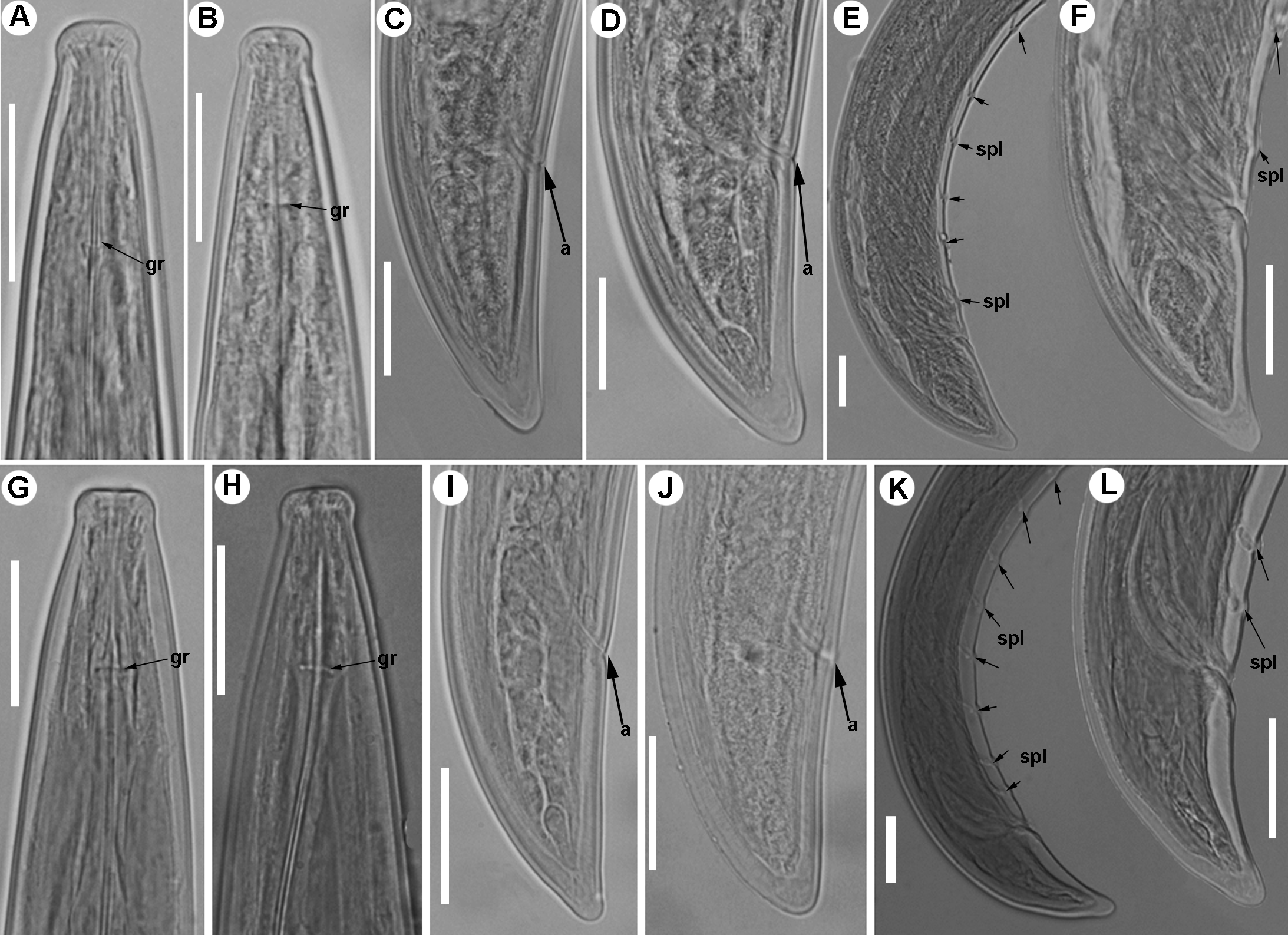

Supplement: S1 Fig — C-D, I-J) Female tails. E-F, K-L) Male tails. Abbreviations: a = anus; gr = guiding-ring; spl = ventromedian supplements. Scale bars A-L = 20 μm. (TIF) [file pone.0147689.s001.tif]

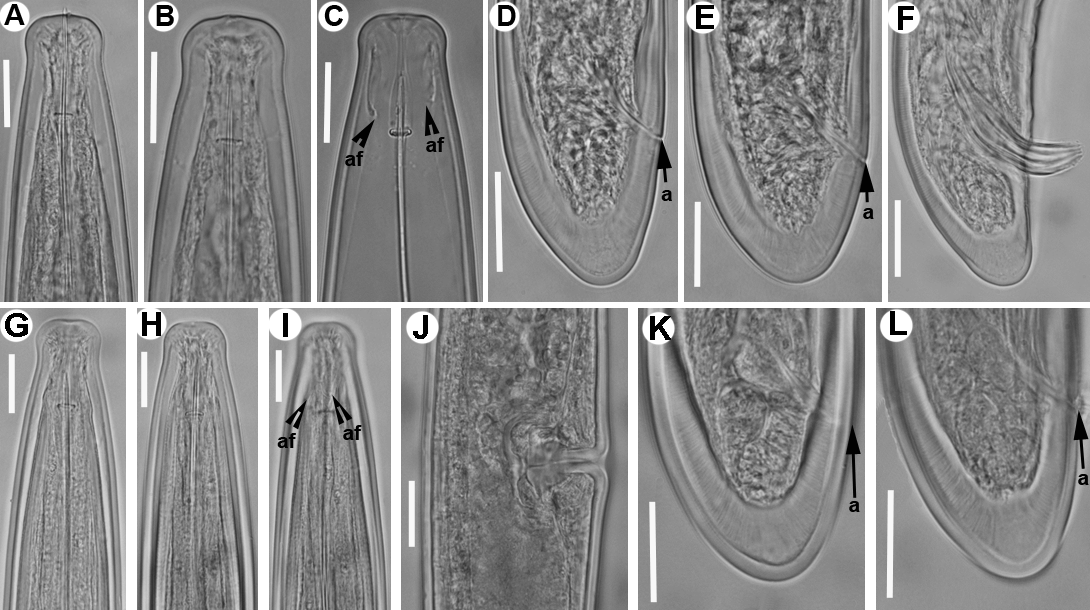

Supplement: S2 Fig — J) Vulval region. D-E, K, L) Female tails. F) Male tail. Abbreviations: a = anus; af = amphidial fovea. (Scale bars = 20 μm). (TIF) [file pone.0147689.s002.tif]

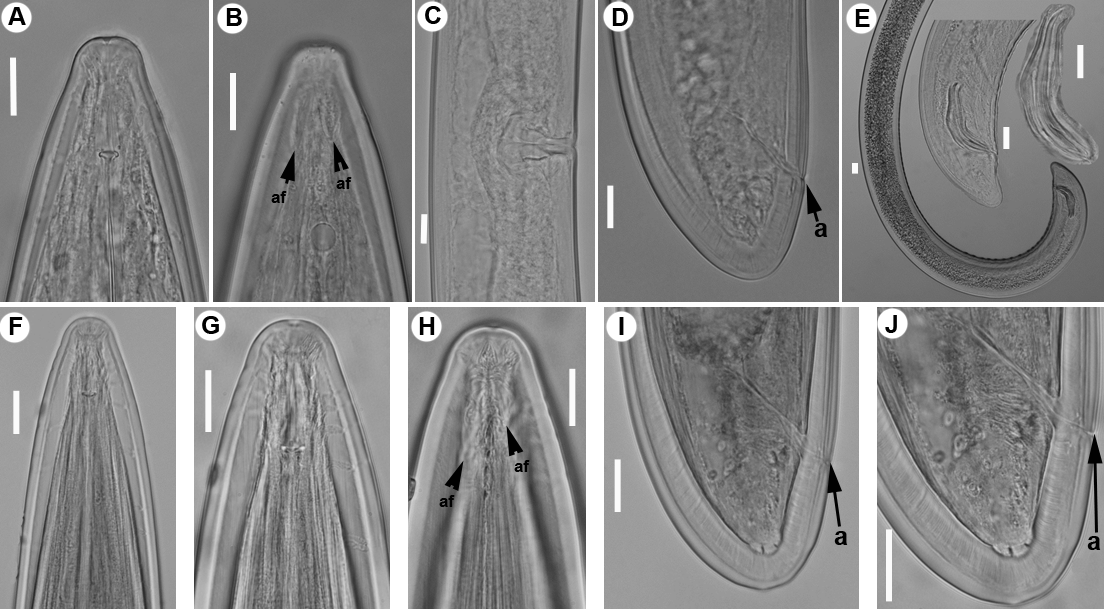

Supplement: S3 Fig — C) Vulval region. D, K, L) Female tails. E-G) Male tails and detail of spicules. Abbreviations: a = anus; af = amphidial fovea. (Scale bars = 20 μm). (TIF) [file pone.0147689.s003.tif]
