# Supplementary material for: Unravelling the Biodiversity and Molecular Phylogeny of Needle Nematodes of the Genus Longidorus (Nematoda: Longidoridae) in Olive and a Description of Six New Species
Source: PLoS One. 2016 Jan 25;11(1):e0147689. doi: 10.1371/journal.pone.0147689 (PMC4726821; doi:10.1371/journal.pone.0147689)
Supplement: S1 Table — (DOC) [file pone.0147689.s004.doc]

**S1 Table.** Morphometrics of *Longidorus* *lusitanicus* Macara, 1986 and *L. oleae* Gutiérrez-Gutiérrez *et al*., 2013 studied from southern Spain^a^.

|  | ***Longidorus lusitanicus*** **Macara, 1986** | |  | ***Longidorus oleae*** **Gutiérrez-Gutiérrez et al., 2013** | |
| --- | --- | --- | --- | --- | --- |
| Locality/host-plant | Bolonia (Cádiz, Spain), wild olive | |  | Córdoba (Córdoba, Spain)  wild olive | |
| Sample code | J212B | |  | AR112 | |
| Characters/ratios ^b^ | Females | Males |  | Females | Male |
| n | 8 | 6 |  | 3 | 1 |
| L (mm) | 5.1 ± 0.52  (4.23-5.75) | 5.1 ± 0.20  (4.71-5.30) |  | 7.8 ± 0.18  (7.5-7.9) | 7.5 |
| a | 95.9 ± 6.1  (89.2-102.9) | 104.2 ± 6.6  (95.3-112.7) |  | 83.4 ± 4.0  (80.5-87.7) | 83.3 |
| b | 15.2 ± 2.0  (12.2-17.2) | 17.4 ± 2.5  (13.9-21.1) |  | 13.8 ± 0.3  (13.6-14.2) | 21.8 |
| c | 166.2 ± 22.6  (141.1-198.3) | 148.4 ± 11.3  (130.7-165.0) |  | 190.3 ± 9.8  (180.2-199.8) | 194.8 |
| c´ | 0.8 ± 0.1  (0.7-0.9) | 1.0 ± 0.1  (0.9-1.1) |  | 0.7 ± 0.1  (0.6-0.7) | 0.7 |
| V | 50.4 ± 1.9  (47.0-54.0) | - |  | 52.2 ± 0.8  (51.5-53.0) | - |
| Odontostyle | 84.8 ± 4.3  (80.0-92.0) | 84.1 ± 3.5  (80.0-88.5) |  | 120.0 ± 13.5  (107.0-134.0) | 118.5 |
| Odontophore | 50.6 ± 1.8  (49.0-54.0) | 50.7 ± 2.7  (48.0-54.0) |  | 51.2 ± 6.0  (46.5-58.0) | 73.0 |
| Lip region diam. | 21.0 ± 0.8  (19.5-22.0) | 21.2 ± 0.9  (20.0-22.5) |  | 14.8 ± 0.3  (14.5-15.0) | 16.0 |
| Oral aperture-guiding ring | 26.9 ± 0.8  (26.0-28.0) | 27.4 ± 2.1  (24.0-30.0) |  | 36.5 ± 1.5  (36.0-38.0) | 37.0 |
| Tail length | 30.9 ± 3.1  (28.0-37.0) | 34.3 ± 1.7  (31.0-36.0) |  | 40.5 ± 1.7  (39.5-42.5) | 38.5 |
| Spicules | - | 51.8 ± 3.2  (48.0-55.0) |  | - | 98.0 |
| Lateral accessory piece | - | 14.8 ± 0.8  (14.0-16.0) |  | - | 25.0 |

^a^ Measurements are in µm (except for L) and in the form: mean ± standard deviation (range).

^b^ Abbreviations as defined in Jairajpuri & Ahmad (1992). a, body length/maximum body width; b, body length/pharyngeal length; c, body length/tail length; c', tail length/body width at anus; V (distance from anterior end to vulva/body length) x 100; T (distance from cloacal aperture to anterior end of testis/body length) x 100; J (hyaline tail region length).
