# Supplementary material for: Unravelling the Biodiversity and Molecular Phylogeny of Needle Nematodes of the Genus Longidorus (Nematoda: Longidoridae) in Olive and a Description of Six New Species
Source: PLoS One. 2016 Jan 25;11(1):e0147689. doi: 10.1371/journal.pone.0147689 (PMC4726821; doi:10.1371/journal.pone.0147689)
Supplement: S2 Table — (DOC) [file pone.0147689.s005.doc]

**S2 Table.** Morphometrics of *Longidorus* *vinearum* Bravo & Roca, 1995 populations studied from southern Spain^a^.

| Locality/host-plant | Santa María de Trassierra (Córdoba, Spain), wild olive | | | | | | | | | | |
| --- | --- | --- | --- | --- | --- | --- | --- | --- | --- | --- | --- |
| Sample code | AR059 | | |  | AR097 | |  | AR066 |  | AR111 | |
| Characters/ratios ^b^ | Females | Male | J1 |  | Females | Male |  | Female |  | Females | Male |
| n | 3 | 1 | 3 |  | 2 | 1 |  | 1 |  | 2 | 1 |
| L (mm) | 7.8 ± 0.38  (7.4-8.1) | 8.1 | 1.7 ± 0.32  (1.4-2.0) |  | 8.4 ± 0.13  (7.5-9.3) | 8.3 |  | 7.1 |  | 7.8 ± 0.11  (6.7-8.9) | 7.3 |
| a | 74.7 ± 3.0  (71.2-76.7) | 70.7 | 46.5 ± 6.6  (39.0-51.6) |  | 75.4 ± 11.0  (67.7-83.2) | 80.8 |  | 70.3 |  | 61.6 ± 2.7  (58.8-64.2) | 64.5 |
| b | 15.4 ± 3.0  (12.5-18.0) | 15.3 | 8.0 ± 0.4  (7.6-8.3) |  | 15.0 ± 1.6  (13.9-16.1) | 15.3 |  | 13.8 |  | 14.4 ± 2.5  (11.6-16.5) | 12.6 |
| c | 177.4 ± 11.8  (167.5-190.5) | 147.9 | 33.1 ± 1.9  (31.1-34.8) |  | 164.6 ± 18.2  (151.7-177.5) | 189.0 |  | 133.4 |  | 160.4 ± 25.3  (141.0-189.1) | 135.5 |
| c´ | 0.6 ± 0.1  (0.6-0.7) | 0.8 | 2.0 ± 0.26  (1.8-2.3) |  | 0.8 ± 0.0  (0.8-0.8) | 0.6 |  | 0.8 |  | 0.7 ± 0.1  (0.6-0.7) | 0.9 |
| V | 46.2 ± 0.8  (45.5-47.0) | - | **-** |  | 47.8 ± 0.4  (47.5-48.0) | - |  | 49.0 |  | 48.2 ± 1.0  (47.0-49.0) | - |
| Odontostyle | 111.0 ± 2.0  (109.0-113.0) | 98.0 | 67.2 ± 1.4  (65.5-68.0) |  | 105.0 ± 0.0  (105.0-105.0) | 109.0 |  | 110.5 |  | 105.2 ± 4.0  (100.5-107.5) | 116.5 |
| Replacement odontostyle | - | - | 71.5 ± 1.8  (70.0-73.5) |  | **-** | - |  | - |  | - | - |
| Odontophore | 54.5 ± 2.3  (52.0-56.5) | 62.5 | 34.5 ± 8.5  (28.5-40.5) |  | 64.0 ± 1.4  (63.0-65.0) | 68.0 |  | 72.0 |  | 75.0 ± 4.4  (72.0-80.0) | 63.0 |
| Lip region diam. | 21.5 ± 0.9  (20.5-22.0) | 24.0 | 9.8 ± 0.8  (9.0-10.5) |  | 21.3 ± 0.8  (20.0-22.5) | 23.5 |  | 21.5 |  | 20.3 ± 1.0  (19.5-21.5) | 20.5 |
| Oral aperture-guiding ring | 35.7 ± 0.8  (35.0-36.5) | 38.0 | 21.5 ± 0.0  (21.5-21.5) |  | 33.8 ± 0.4  (33.5-34.0) | 38.5 |  | 38.0 |  | 34.8 ± 3.2  (32.5-38.5) | 36.0 |
| Tail length | 44.3 ± 3.6  (42.0-48.5) | 55.0 | 50.3 ± 6.8  (44.0-57.5) |  | 51.0 ± 2.1  (49.5-52.5) | 44.0 |  | 53.5 |  | 49.2 ± 6.0  (44.5-56.0) | 54.0 |
| Spicules | - | 100.0 | - |  | - | 105.0 |  | - |  | - | 109.0 |
| Lateral accessory piece | - | 20.0 | - |  | - | 21.5 |  | - |  | - | 26.5 |

^a^ Measurements are in µm (except for L) and in the form: mean ± standard deviation (range).

^b^ Abbreviations as defined in Jairajpuri & Ahmad (1992). a, body length/maximum body width; b, body length/pharyngeal length; c, body length/tail length; c', tail length/body width at anus; V (distance from anterior end to vulva/body length) x 100; T (distance from cloacal aperture to anterior end of testis/body length) x 100; J (hyaline tail region length).
